# Impact of a Multicomponent Intervention to Build Capacity of Public Health Workers to Make Algorithmic Diagnosis and Management of High-Risk Pregnancies in Uttar Pradesh, India: Protocol for a Matched-Control, Before-After, Quasi-Experimental Study With a Mixed Methods Design

**DOI:** 10.2196/74993

**Published:** 2025-12-09

**Authors:** Hanimi Reddy Modugu, Chetan Purad, Venkatesh Irugulapati, Anil Kumar Shukla, Sandhya N V S Dittakavi, Siri Chandana, Anuja Jayaraman, Karishma Thariani, Aparna Hegde

**Affiliations:** 1 Research Department, ARMMAN, 4th floor, Amogh Plaza, Teachers Colony, Begumpet, Hyderabad, 500 016, India, 91 9911822445; 2 Integrated High-Risk Pregnancy Tracking and Management (IHRPTM) Program, ARMMAN, Hyderabad, India; 3 ARMMAN, Lucknow, India; 4 ARMMAN, Mumbai, India; 5 Fellowship Urogynecology and Pelvic Reconstructive Surgery (AIIMS), New Delhi, India

**Keywords:** antenatal care, before-after, capacity building of health workers, diagnosis and management, high-risk pregnancies, impact of program, intervention control arm, quasi experimental

## Abstract

**Background:**

In India, 20%‐30% of pregnancies are high-risk, contributing to 75% of perinatal mortality and morbidity. An effective approach to reduce this mortality and morbidity is early identification, effective management, and timely referral of high-risk pregnancies (HRPs). The Integrated High-Risk Pregnancy Tracking and Management program aims to enhance the capacity of auxiliary nurse midwives (ANMs), medical officers (MOs), and specialist gynecologists by (1) providing algorithmic, color-coded, detailed yet simple HRP protocols, customized for each role; (2) offering live training; (3) delivering digital training and handholding; and (4) tracking management details for HRPs. Equipping health workers (HWs) with these interventions supports early identification, effective management, and timely referral of HRPs, ultimately improving primary care and enhancing satisfaction of mothers with HRPs. These interventions are implemented in the intervention arm for over 18 months. During this time, HWs of intervention and control arms will continue to receive routine training through state and national programs, and pregnant women of both arms have access to standard maternity services.

**Objective:**

At the system level, the program evaluates the impact on the knowledge and skills of HWs in diagnosing and managing HRPs. At the community level, it assesses translation of this knowledge into practice in terms of early diagnosis and protocol-based management of women with HRPs.

**Methods:**

The program will be implemented in 2 intervention (Sambhal and Shravasti) and 2 matched control districts (Budaun and Gonda) of Uttar Pradesh, on 6 HRPs. The study uses a “quasi-experimental, before-and-after design” with intervention and control arms. However, the impact of the program will be assessed only on 3 HRPs: moderate or severe anemia, pregnancy-induced hypertension, and antepartum hemorrhage. System-level impacts will be assessed through qualitative data from district officials, specialist gynecologists, MOs, and ANMs at baseline and end line. The community-level impacts will be assessed by comparing quantitative data from recently delivered women at baseline and end line. Community-level impact assessed using the difference-in-difference (DiD) technique. The study received ethical approval.

**Results:**

By November 2024, all the ANMs, MOs, specialist gynecologists, staff nurses, and community health officers in 2 intervention districts were trained on 6 HRP protocols, after the protocols were vetted by the Government of Uttar Pradesh. A digital learning tool and WhatsApp-based support system were introduced to facilitate continued learning and handholding of ANMs in managing HRPs and to address queries. Baseline data were collected from 2 arms during June-October 2024.

**Conclusions:**

This trial will provide valuable insights into the feasibility and effectiveness of the program at system and community levels in an emerging state like Uttar Pradesh. These insights can feed into capacitating HWs across all the districts and support expansion of the program to include additional HRPs, with significant potential for improving maternal and neonatal outcomes.

## Introduction

### Overview

Most maternal health complications that lead to maternal or newborn death develop during pregnancy, and with quality antenatal care (ANC), most of these complications can be prevented or treated [1]. Although the goal is to ensure a nonproblematic pregnancy, all pregnancies carry some risk. Pregnancy risks are categorized as low, moderate, and high risk [2]. A high-risk pregnancy (HRP) is defined as one that is complicated by factors that adversely affect the pregnancy outcome (maternal, perinatal, or both). An HRP may result from conditions that exist either before pregnancy (eg, diabetes and hypertension) or complications from a previous pregnancy, or conditions that develop during pregnancy or delivery [3].

Globally, around 20 million pregnancies are classified as high-risk, leading to approximately 800 maternal deaths per day [4], and over 70% of maternal deaths are because of complications of pregnancy and childbirth, such as hemorrhage, hypertensive disorders, sepsis, and abortion [5]. Excluding moderate anemia, 30%‐37% pregnancies globally can be classified as high-risk, accounting for 70%‐80% of perinatal mortality and morbidity [6]. In India, 20%‐30% pregnancies are high-risk and are responsible for 75% of perinatal morbidity and mortality [7]. According to a recent (2019‐2020) analysis in India, prevalence of HRPs was 49.4%, with 33% having single high-risk condition and 16.4% having multiple high-risk conditions [8]. Prevalence of HRPs across different states ranges from 18% to 59% [9-12], perhaps due to differences in estimation methods. According to the National Family Health Survey (NFHS)-5 (2019‐2021) estimate, 59% of Indian women were anemic. Despite high levels of anemia, only 44% of pregnant women consumed the recommended dose of iron-folic acid (IFA) for 100 days [13]. The only effective approach to reducing maternal and neonatal mortality and morbidity resulting from HRPs is early identification, timely referral, and efficient management throughout pregnancy before conditions progress to a critical stage.

In India, particularly in rural areas, routine ANC, including HRP identification, management, and referral, is provided at 3 primary care facilities: (1) health subcenters (HSCs) managed by auxiliary nurse midwives (ANMs), (2) primary health centers (PHCs) by medical officers (MOs), (3) and community health centers (CHCs) by specialist gynecologists, while women with HRPs are mostly referred to advanced centers in cities. Textbox 1 lists the responsibilities of ANC providers. The HSCs primarily provide ANC and birthing services; PHCs provide either basic emergency obstetric and newborn care (BEmONC) or only birthing services; and CHCs, although, are supposed to provide comprehensive emergency obstetric and newborn care (CEmONC), mostly provide BEmONC or even less. These facilities primarily focus on child birthing services and fail to address management of complications in early pregnancy [14]. Also, there are gaps in knowledge of emergency treatment for obstetric complications during pregnancy and prereferral first aid. Staff generally lack confidence and do not have adequate resources to manage obstetric complications [15]. Staff nurses, posted at PHCs and CHCs, have a limited role in decision-making when it comes to managing these complications. However, all the staff desire skill building, mentoring, moral support, and motivation [15]. Studies have identified deficits in maternal and newborn-related skills among ANMs, highlighting the need to strengthen their skills, especially through in-service training [16]. In addition to these gaps, there is often a confusion regarding the individual roles of the ANM, MO, and specialist gynecologist in managing HRP conditions, resulting in many pregnant women falling through the gaps in the system after being diagnosed with an HRP condition. With limited or no resources to manage HRPs, staff in these 3 settings should be highly competent in prevention, risk assessment, stabilization of complicated HRPs, and arranging transfer and care at functional higher referral levels [17].

Textbox 1. **Responsibilities of antenatal care (ANC) providers.**

**Auxiliary nurse midwives (ANM)**

- Placed at health subcenter (1 per 5000 population; 3000 in tribal areas).
- Provides ANC, vaccinations, iron supplements, safe deliveries, postnatal care, newborn immunization, and family planning.
- Identifies high-risk pregnancies, facilitates referrals, and maintains health records.

**Medical officers**

- Placed at primary health center (1 per 30,000 population).
- Supervises ANMs and nurses, conducts ANC, manages pregnancy complications, performs normal deliveries, and handles obstetric emergencies.
- Initiates referrals, administers life-saving treatments, and supports family planning services.

**Specialist gynecologists**

- Placed at community health centers (1 per 120,000 population) and district hospitals.
- Manages high-risk pregnancies, performs cesarean sections, and handles obstetric emergencies.
- Provides specialized ANC, supervises labor, trains staff, and ensures quality maternal care.

**Accredited social health activist**

- Placed at the village level (1 per 1000 population).
- Mobilizes pregnant women for ANC, institutional deliveries, and immunization. Educates on nutrition, danger signs, and family planning.
- Assists in postnatal care, escorts women to facilities, and facilitates Janani Suraksha Yojana benefits.

**Anganwadi worker**

- Placed at anganwadi center (1 per 1000 population; 700 in tribal areas).
- Supports maternal and child health under the Integrated Child Development Services.
- Provides supplementary nutrition, monitors growth, promotes ANC, immunization, breastfeeding, and family planning.
- Identifies malnutrition, educates mothers, and coordinates with accredited social health activists and ANMs for health care referrals.

The following literature provides quantitative evidence on the extent of gaps in correct management and referrals to HRPs at different levels of the public health care system in low- and middle-income countries, including India. A systematic review in India reported that due to the inability of PHCs to provide basic HRP care, the proportion of unjustified referrals to higher-level facilities ranged between 25%‐52% [18]. A study from Himachal Pradesh and Andhra Pradesh (AP) in India highlighted systemic gaps among health workers (HWs) across 3 tiers of the public health care system, as only 31% (47/147) of HWs from 3 tiers of the public health care system reported screening only for 10 out of 16 most common HRP conditions. Even among the 10 HRPs screened, only 35% (17/49) of HWs at PHCs and 51% (18/35) at CHCs reported managing identified HRPs at their facilities, while the remainder referred such cases. To specifically assess HRP management competencies, scenario-based assessments were conducted; “a second-trimester moderate anemia case scenario was given to HWs at 3 tiers of health care system in 2 states.” While nearly all HWs correctly diagnosed the condition and over 75% prescribed oral iron, only 5%‐45% prescribed iron injection, negligible prescribed tablet Mebendazole, and <10% provided nutritional counseling. Around 50% of HWs across tiers referred the case, although PHCs and CHCs are supposed to manage moderate anemia cases. Similarly, when a third-trimester pregnancy-induced hypertension (PIH) case scenario was given, all HWs made correct diagnosis. However, appropriate treatment varied, with 42% of PHCs and 71% of CHCs prescribing antihypertensive drug (MgSO_4_). Additionally, 94% at HSCs, 85% at PHCs, and 45% at CHCs reported referring such cases, although CHCs are supposed to manage PIH and preeclampsia cases. Thus, this study concluded that most referrals were very early and unnecessary, and few delayed referrals were coupled with poor prereferral management [15]. On preeclampsia and eclampsia diagnosis and management in 6 African countries, knowledge of diagnosis was excellent (>80%) in HWs, while knowledge on correct management varied (33%‐77%) and management in the event of convulsions was ≤51% [19]. A situational assessment study conducted in Telangana, India (2021‐2022) found that ANMs mainly manage anemia, and 94% of them refer all high-risk cases to specialists, thereby bypassing the established referral chain. The MOs and specialists also felt the need for training support in managing HRPs, as 25% of MOs lack clarity and confidence in handling HRPs, and only 60%‐65% of specialists possessed the requisite knowledge [20]. An important reason for suboptimal knowledge, lack of confidence, and confusion in role clarity in HRP management and referral across the 3 tiers of the health care system is the absence of standardized management and referral protocols for HRPs tailored according to these 3 tiers of health care system in India and other low- and middle-income countries.

For early identification, management, referral, and follow-up of HRPs, all pregnant mothers need to undergo screening for HRPs, and those identified should be managed and followed up regularly [21]. The World Health Organization’s (WHO’s) 2015 guidelines on HRPs recommend early assessment, as they serve as a guide for clinical decisions and are primarily intended for primary care at the facility and community level [22]. Although the revised guidelines describe the management of obstetric complications at the district level according to the period of gestation [23], their application at different facilities and levels of referral system in low-resource settings is not well understood [15]. In India, the skilled birth attendant guidelines on HRP management and referral provide guidance only on clinical management of low-risk and common HRPs and complications during pregnancy, childbirth, and postpartum care, and suggest referral, if the facility is incapable of managing a case [24]. Although in India, programs like Pradhan Mantri Surakshit Matritva Abhiyan, Labor Room Quality Improvement Initiative, Surakshit Matritva Aashwasan, Live Saving Anaesthesia Skills, etc, were initiated to address HRPs [25], their focus is primarily on strengthening BEmONC and CEmONC services, with limited or no focus on building the capacity of ANMs at HSCs or MOs at PHCs, who are the first point of contact for mothers with HRP conditions.

### Problem Statement and Research Gap

Thus, in India, despite programs like Pradhan Mantri Surakshit Matritva Abhiyan and Surakshit Matritva Aashwasan, the provision of continuum of care for HRPs remains inadequate. This includes gaps in early identification, understanding causes and complications, appropriate treatment and referral, follow-up care, including need-based laboratory investigations and follow-up after discharge or until safe delivery [26]. This may be due to (1) nonavailability of evidence-based, condition-specific, and role-specific guidelines for identification and management of HRPs, (2) confusion regarding specific roles of HWs in managing the HRPs, (3) and poor knowledge, skills, and lack of confidence, particularly among ANMs and MOs. As a result of all these issues, there is an increase in irrational or unnecessary referral of women with HRPs to higher facilities, thereby compromising on the quality of ANC. Thus, we strongly believe that, through effective management of HRPs and by preventing pregnancy complications in non-HRP women, the quality of ANC services can be ensured, leading to a significant impact on reduction in maternal deaths, stillbirths, and early neonatal deaths—a triple return on investment [27].

We also identified the following research gap that, although several programs under the National Health Mission seek to strengthen frontline HWs’ knowledge and skills related to maternal and child health outcomes, few studies in India have rigorously examined providers’ perspectives on HRP screening and management [28]. Evidence on the effects of capacitating ANMs and MOs in HRPs management and referral is even more limited, particularly with respect to system-level outcomes (eg, improvements in clinical knowledge, diagnostic capacity, and confidence in managing HRPs) [15], as well as community-level outcomes (eg, improvement in quality of ANC services and the appropriate diagnosis and management of HRPs as reported by pregnant women). To the best of our knowledge, we have not come across studies that have assessed the impact of capacitating ANMs, MOs, and specialists on strengthening HRP management skills and ensuring appropriate care for women with HRPs in Uttar Pradesh (UP), India’s most populous state. Our study addresses this critical gap in a context characterized by a high maternal mortality ratio of 141 per 100,000 live births in 2020‐2022 [29].

### How Our Interventions Will Address the Problem

The Integrated High-Risk Pregnancy Tracking and Management (IHRPTM) program by ARMMAN (Advancing Reduction in Mortality and Morbidity of Mothers, Children, and Neonates)*,* a nonprofit organization, has been improving health-seeking behavior of pregnant women by building capacities of HWs to ensure efficient antenatal and child care, and timely diagnosis, management, and referral of HRPs. The program is designed to address gaps in HRP management through an integrated, comprehensive, multistep, systemic approach for improved identification, tracking, and end-to-end management of HRPs. Its aim is to reduce delayed, complicated, and irrational referrals to tertiary facilities, allowing these facilities to focus on truly critical cases, leading to satisfaction of women and families with HRP conditions. By capacitating, motivating, and handholding HWs, we anticipate the prevention of pregnancy complications among non-HRP women. Integration of different interventions also leads to improvement in quality of ANC services, thereby lowering overall maternal and neonatal mortality and morbidity in the intervention area.

### Key Interventions of the Program

The program includes several key interventions to strengthen the management of HRPs. Color-coded protocols are developed as algorithmic tools that are simple yet comprehensive, designed for the end-to-end management, referral, and follow-up of 35 HRPs. These protocols are tailored for ANMs, MOs, and specialist gynecologists. They remain concise and uncluttered, ensuring absolute clarity at every level while adhering to local practicalities.

Live training sessions are conducted for HWs (ANMs, MOs, and specialists) to enhance their skills in identifying and managing HRPs within the facilities where they work. The training refreshes their knowledge and skills on the early diagnosis of HRPs and provides them with tailored decision-support tools in the form of protocols. These tools guide case management based on the resources available at the facility and offer algorithm-based instructions for referring cases that cannot be managed locally to higher-level facilities.

To encourage continuous, self-paced learning, the program also introduces a digital learning app designed specifically for ANMs. The app contains learning materials, such as videos, Microsoft PowerPoint presentations, practice questions, and quizzes, all organized according to HRP protocols. A new protocol is released every 2-3 weeks. As a self-paced learning tool, the app enables ANMs to upgrade their skills at their own convenience.

In addition, a WhatsApp-based support helpline combines mobile technology with personalized guidance, enabling doubt resolution and handholding for ANMs as they engage with the learning content and apply it in real-life field situations. Queries raised by ANMs are answered within 24 hours by state-appointed MOs, trainers-of-trainers, or dedicated ARMMAN staff. Together, the learning app and the WhatsApp helpline create an almost real-time platform for addressing doubts related to the management and referral of HRP cases.

Finally, HRP-specific data are linked with the state’s monitoring system to improve tracking and decision-making across different levels of the public health care system. This digital tracking system functions as a real-time data collection and decision-support tool integrated with the state’s HRP monitoring platform (Reproductive and Child Health [RCH] portal). It captures the management and referral journey of women with HRP conditions from anywhere in the state. The tracking app also guides HWs in recording relevant signs, symptoms, and diagnostic interventions, thereby ensuring that appropriate care is provided at every stage of management and referral for women with HRP conditions.

Our interventions are human-centric and user-focused, incorporating facility-specific actions tailored to the needs and responsibilities of each cadre within the health care system. The vision of the IHRPTM program is to improve the functioning of the public primary health care system and achieve a sustained reduction in irrational, delayed, and complicated referrals to higher-level facilities. This will ensure HSCs and PHCs manage mild to moderate HRPs, while only serious cases are referred to higher facilities. In turn, this reduces the burden on secondary and tertiary facilities, thereby improving the functioning of the 3 tiers of public health care system.

Thus, our innovative “tech plus touch model” leverages the existing public health care system to strengthen the capacity of HWs to manage HRPs, thereby facilitating scalability across districts or states. This approach includes an inbuilt sustainability model, as the IHRPTM program is integrated within the existing public health care system, without creating a parallel structure that would be resource intensive in terms of human and financial capital. Ultimately, a capacitated health care system and its stakeholders will create demand among mothers with HRPs for timely and appropriate care.

### Interventions and Anticipated Theory of Change

**Figure 1.**
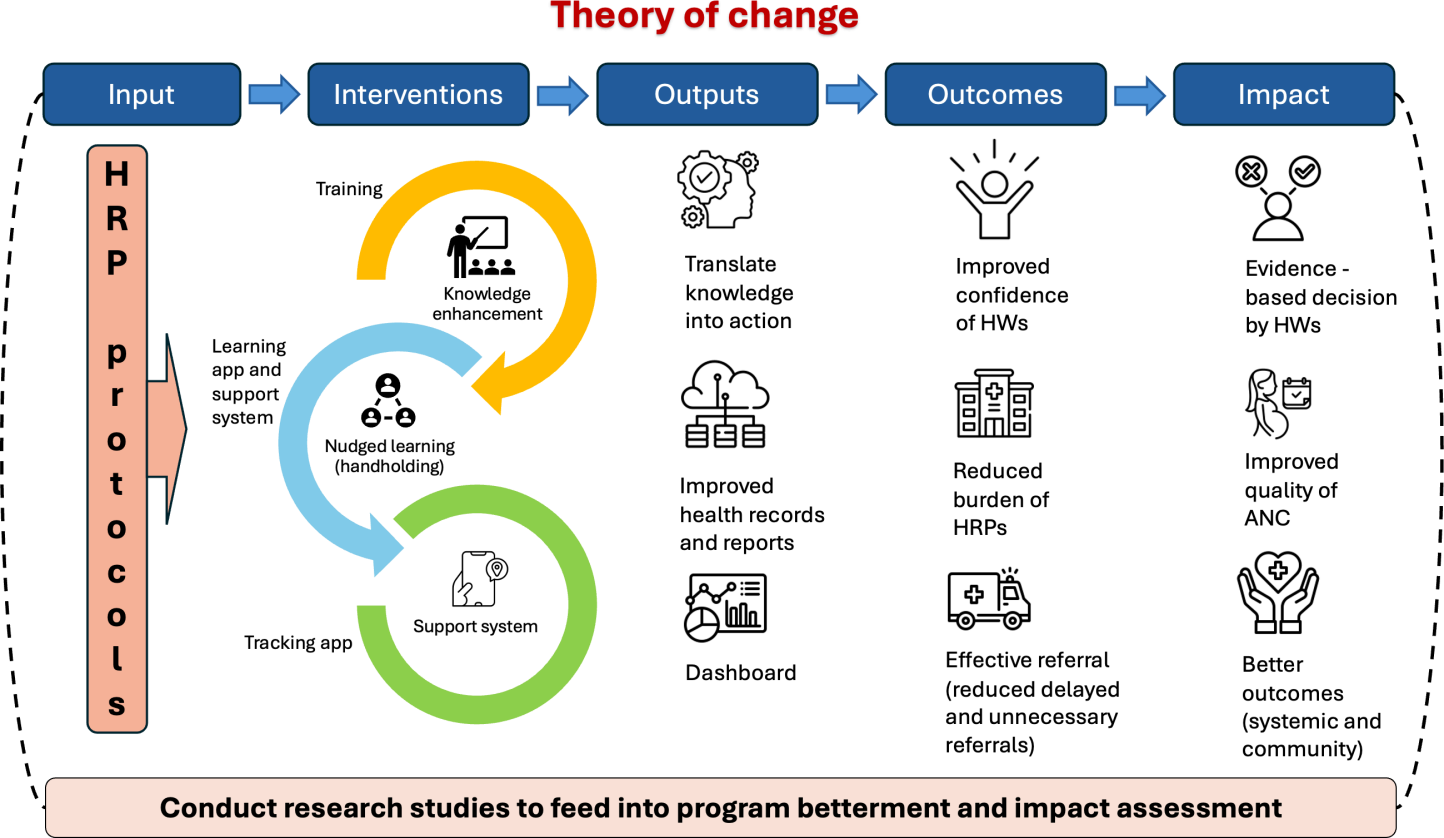
Logical framework conceptualizing the theory of change of program to improve knowledge and skills of health workers (HWs) to diagnose and manage high-risk pregnancies (HRPs). ANC: antenatal care.

The program’s theory of change is shown in Figure 1, with inputs and interventions leading to short-term outputs, which in turn lead to medium-term outcomes, and finally long-term impacts. The HRP condition–specific, algorithm-driven, color-coded protocols tailored for 3 cadres of HWs serve as readily accessible reference documents for managing HRP cases. Since these protocols have been vetted and formally endorsed by the UP health department, and since ARMMAN is implementing the program in partnership with health departments of 2 intervention districts, we anticipate cooperation from district health systems throughout the 18 months of the implementation period. This cooperation is expected to include nomination of ANMs, MOs, and specialists for training and other supportive supervision initiatives. Classroom training on HRP protocols is designed to refresh and strengthen HW’s knowledge and skills to manage HRPs. It is expected to provide them with around 10% immediate gain in theoretical knowledge and skills to manage or refer HRPs (based on evidence from phase-1 protocols in Telangana, which showed 8%‐17% gain in knowledge among ANMs) [30]. Training supplemented with digital interventions provides HWs, particularly ANMs, with real-time responses to queries they get during management or referral of HRP cases in actual field conditions. These processes translate knowledge into practice, resulting in early diagnosis and appropriate management or referral of the HRP cases according to type of health facility (short-term output).

When training and digital interventions are supplemented with the tracking app, comprehensive details on the management and referral pathways adopted for each HRP case are generated. These cumulative data lead to the creation of dashboards on the IHRPTM program at different levels of health care system (short-term output). With continued handholding of interventions over 9‐12 months, the ANMs and MOs are expected to gain confidence to diagnose and manage HRPs as per the protocols, and there will be a reduction in the number of delayed or unnecessary referrals of HRP cases to higher facilities, and thus ultimately leading to a reduction in the burden of HRPs (medium-term outcomes).

Finally, when all the ANMs and MOs in the intervention districts consistently manage or refer HRPs using standardized protocols over a 12‐18-month period, we anticipate impact on both system- and community-level outcomes. These include evidence-based management of HRPs by all 3 cadres of HWs, timely relief of women diagnosed with HRPs prior to delivery, and ultimately, improvement in overall quality of ANC (long-term impacts).

### Anticipated Challenges to Program Implementation

Our program, implemented in partnership with the district and state health department, is both a source of strength and a potential challenge. In the intervention arm, the program must navigate several systemic issues, including the double burden of noncommunicable and communicable diseases, reproductive health problems, underfunding, weak procurement and logistics systems (eg, supply of essential medicines and vaccines), an uneven distribution of HWs, weak regulatory mechanisms, and issues such as inappropriate prescription and use of medicines [31]. Through our handholding interventions and continuous engagement with stakeholders across the 3 tiers of the public health care system, we aim to mitigate some of these challenges.

Almost all the ANMs in UP have an internet-enabled smartphone or tablet. However, despite this universal access, a feasibility assessment and user feedback study conducted in Telangana with 2790 ANMs identified several digital challenges. These included permission restrictions for installing digital apps, poor internet speed, login difficulties, overheating or slow device performance, delays in receiving new content in digital apps, data inaccuracies, and issues with switching languages in the learning management system app. We expect similar digital challenges in the 2 intervention districts. The program addresses these issues through continuous handholding and support.

As our program does not work with private or unregulated health care sector, there is a lack of data on the capacity of these players in HRP management. However, in UP, around 30% of the ANC services were exclusively provided by private or NGO (nongovernmental organization) sector and an additional 13.5% by a combination of public or private or NGO sector providers [13,30]. This implies that our program interventions will not influence or will only partially influence 30%‐44% of pregnant women in the state. Also, women seeking ANC or HRP services from private or NGO providers are going to influence the quality of ANC indicators at community level.

### Rationale for the Study

#### Overview

The IHRPTM program in UP was developed with a comparative advantage, as it was informed by learnings from the implementation of 6 phase-1 protocols in Telangana and AP. In UP, the program was designed for a shorter duration (18 months vs 5 years in Telangana and AP), on a smaller scale (6 protocols vs 21 in Telangana and 20 in AP), and in a more limited geography (2 districts in UP vs 33 in Telangana and 26 in AP) as IHRPTM program was initiated as part of UP Government’s Ending Preventable Maternal Mortality (EPMM) initiative launched in mid-2024. As the impact evaluation of the IHRPTM program in Telangana and AP is going to take more time (2027 or beyond), the government of UP, before implementing the program at scale across all the 75 districts with around 20 HRP conditions, decided to first implement the program on a pilot basis. This pilot phase is of shorter duration (18 months), limited to 6 HRP conditions, and covers only 2 intervention districts. The 6 HRPs prioritized by the government of UP are quality ANC (QANC), anemia (moderate and severe), PIH, antepartum hemorrhage (APH; including placenta previa and abruptio placenta), heart disease or shortness of breath, and fever. Hence, the UP initiative was conceptualized as a pilot project, with the explicit aim of informing future scale-up across all 75 districts, expanding to additional HRP protocols, and generating early evidence of the program’s impact at both system and community levels.

While designing the impact evaluation of the UP pilot, the following contextual variations in the scale of direct beneficiaries were considered. The program engaged ANMs (>400 in UP vs >9000 in Telangana and >15,000 in AP), MOs (>100 in UP vs >1000 in Telangana and >1800 in AP), and specialists (>10 in UP vs >400 in Telangana and >500 in AP). Indirect beneficiaries also varied substantially, with approximately 60,000 pregnancies per year in UP compared with 700,000 in Telangana and 900,000 in AP, translating to roughly 18,000 HRPs per year in UP versus 200,000 in Telangana and 300,000 in AP. Training timelines also differed, while training all ANMs on 6 phase-1 protocols required approximately 1 year in Telangana and 9 months in AP, >400 ANMs in UP were trained in just a few days. Moreover, digital interventions were introduced within 2-3 months of program initiation in UP, compared with more than 3 years in Telangana and 1 year in AP, as these digital interventions were still evolving when implementation began in Telangana.

Thus, considering UP’s comparative advantage over Telangana and AP, sufficient number of direct and indirect beneficiaries, the rapid introduction of all interventions in quick succession, and additional scope for keeping a control arm, the UP impact evaluation design is expected to provide generalizable evidence on the program’s impact, even with its shorter duration (18 months) and smaller scale (2 intervention districts and 3 HRP protocols). In UP, the impact evaluation will be confined to the following 3 HRP conditions: anemia (moderate and severe), PIH, and APH.

#### Reasons for Not Conducting Impact Evaluation on the Below HRPs

Quality ANC is excluded from the impact evaluation as it is not an HRP condition but rather a set of well-established, evidence-based processes that health functionaries are expected to follow across the 3 levels of public health care system. ARMMAN has included QANC as one of the protocols as it sensitizes ANMs and MOs to the importance of a comprehensive history taking and conducting trimester-specific ANC examinations and tests, thereby contributing to overall quality of the ANC services. The program’s effect on QANC will be assessed using the following proxy indicators: proportion of women who received 3 essential tests and examinations (weight, hemoglobin, and blood pressure) in all the ANC visits, proportion of women who received a complete ANC package (at least 4 ANC visits, at least 1 tetanus toxoid injection, and consumed at least 100 IFA tablets or consumed IFA for a minimum of 100 days), and proportion of women whose third trimester ANC visit took place at CHC or higher-level facility.

Heart disease or shortness of breath is excluded from impact evaluation due to its low prevalence (0.3%‐3.5%) [32]. Evaluating changes for a condition with such a low prevalence over an intervention period of 18 months would require an unfeasibly large sample size of recently delivered women (RDW) with the condition. Also, severe cases with this condition require care at medical college hospitals or specialized facilities with 24×7 availability of cardiologists, surgeons, anesthetists, etc [33], are beyond the scope of this program.

Fever in pregnancy is excluded from impact evaluation, as it is generally a comorbidity associated with non-HRP conditions (eg, dengue, hepatitis E, urinary tract infection, and vector- or water-borne diseases) [34].

#### Hypothesis and Outcomes

The program’s impact evaluation will test the following research hypotheses: (1) Does the program, implemented over 18 months, enhance knowledge, skills, and confidence of the ANMs and the MOs to make early diagnosis and manage the 3 HRPs in the intervention arm, compared to usual care arm? (2) Can the capacitated ANMs and MOs translate their knowledge and skills into practice, particularly for early diagnosis and end-to-end management of the 3 HRP conditions in the intervention arm, compared to the control arm? (3) Does the program improve the quality of ANC services in the intervention arm, compared to the usual care arm?

At the system level, the primary outcomes focus on changes in the knowledge and skills of HWs. The first outcome is the net change in correct knowledge and skills of ANMs to manage (treat, counsel, refer, and follow-up) women with any of the 3 HRP conditions (anemia, PIH, and APH) from baseline to end line, comparing intervention and control arms. The second outcome is the net change in correct knowledge and skills of MOs to manage (treat, counsel, refer, and follow-up) women with any of the 3 HRP conditions (anemia, PIH, and APH) from baseline to end line, comparing intervention and control arms. The percentage of correct knowledge and skills for ANMs and MOs will be calculated based on the number of correct responses provided out of a total of 45 questions on the 3 HRPs, administered at baseline and end line surveys.

The system-level secondary outcomes include the net change in the ability of ANMs to correctly identify HRP-related signs and symptoms from baseline to end line, in intervention versus control arm and net change in the ability of MOs to do the same. Other outcomes include changes in the confidence level of ANMs and MOs to manage HRPs between baseline and end line across 2 arms and to document qualitatively whether any systematic barriers reported by district officials at baseline survey were overcome by the end line, by comparing intervention and control arms.

At the community level, the primary outcome includes net change in the proportion of RDW with any of the 3 HRP conditions (anemia, PIH, and APH) whose problem was resolved prior to delivery and who reported satisfaction with the management of the condition, from baseline to end line, in intervention versus control arm. Satisfaction level of RDW is measured using a 5-point Likert scale on the topics of “attitude and behavior of staff,” “cleanliness and hygiene,” “comfort and care,” and “overall satisfaction with management of HRP condition at the facility.”

The community-level secondary outcomes include net change in proportion of RDW who received the complete ANC package during their last pregnancy*,* from baseline to end line, in intervention versus control arm. Also includes the net change in proportion of RDW who were sensitized about HRPs by ANM or MO during their last pregnancy*,* during baseline and end line, in intervention versus control arm. Other secondary outcomes include net change in the mean gestational month of first diagnosis of 3 HRPs by an ANM*,* from baseline to end line, in intervention versus control arm, and net change in the proportion of RDW diagnosed with any of the 3 HRPs who received protocol-based management or referral from an ANM*,* from baseline to end line, in intervention versus control arm. We also measure the net change in proportion of RDW diagnosed with any of the 3 HRPs, received protocol-based management or referral from MO*,* from baseline to end line in intervention versus control arm.

## Methods

### Study Design

The study adopts a “matched, before-after quasi-experimental design with a control (usual care) arm,” as a randomized control study design is not feasible for this kind of system and community-based implementation study. Both qualitative and quantitative data will be collected at the system and community levels, respectively, during baseline (prior to implementation of interventions) and end line (after 18 months of interventions)*,* in the intervention and control arms. The program’s effectiveness at the system level will be evaluated by assessing the capacity of ANMs and MOs to diagnose and manage the 3 HRPs and at the community level by measuring the proportion of RDW with any of the HRP conditions, whose HRP problem got resolved prior to delivery and who were satisfied with the care they have received for the HRP condition*.* Effectiveness of the program through quantitative indicators will be analyzed using the difference-in-difference (DiD) technique.

However, keeping in view the quasi-experimental design of this study, to control for potential baseline differences between intervention and control arms due to nonrandom allocation, we will lock the analysis plan by prespecifying primary outcomes and covariates of the quantitative data. Prior to advanced analyses, we will implement near-matching of intervention and control data on key covariates, such as age, parity, caste, and literacy. In addition, during the main analysis stage, we will apply DiD models with covariate adjustment to further control for any residual imbalance and to strengthen causal inference.

### Duration of Interventions and Justification for Creating a Usual Care Arm

In 2 intervention districts, the IHRPTM program will be implemented district-wide for 18 months, starting June-July 2024 and ending February-March 2026. The 18-month duration is necessary, as the introduction of different interventions in both the intervention districts requires 3‐6-month time. Also, for converting system level changes into outcomes is a time-consuming process [35]. Given the relatively short implementation period, we are not proposing a midline evaluation. However, the uptake, quality, and coverage of interventions will be continuously monitored through a robust monitoring system. Dipstick studies will be conducted depending upon the need. Any mid-course corrections will be based on findings from the baseline, monitoring data, and dipstick studies.

In the usual care arm, HWs will receive routine capacity-building sessions conducted by the state and the national governments. Pregnant women in both arms will have access to antepartum, intrapartum, and postpartum care programs provided by the state and the national governments. It implies that while general system strengthening interventions are provided in both the arms, the IHRPTM interventions are the only additional interventions confined to the intervention arm. At the community level, the IHRPTM program does not directly work with pregnant women in either arm. The control arm was included to isolate the effect of the IHRPTM interventions from the general system strengthening activities and to assess whether IHRPTM interventions had any incremental effect or value addition beyond what general system strengthening interventions alone can achieve. Thus, this study design ensures ethical equity while also allowing us to isolate the incremental effect of the IHRPTM program interventions by comparing changes in outcomes that go beyond improvements expected from broader system strengthening efforts.

### Study Geography

**Figure 2.**
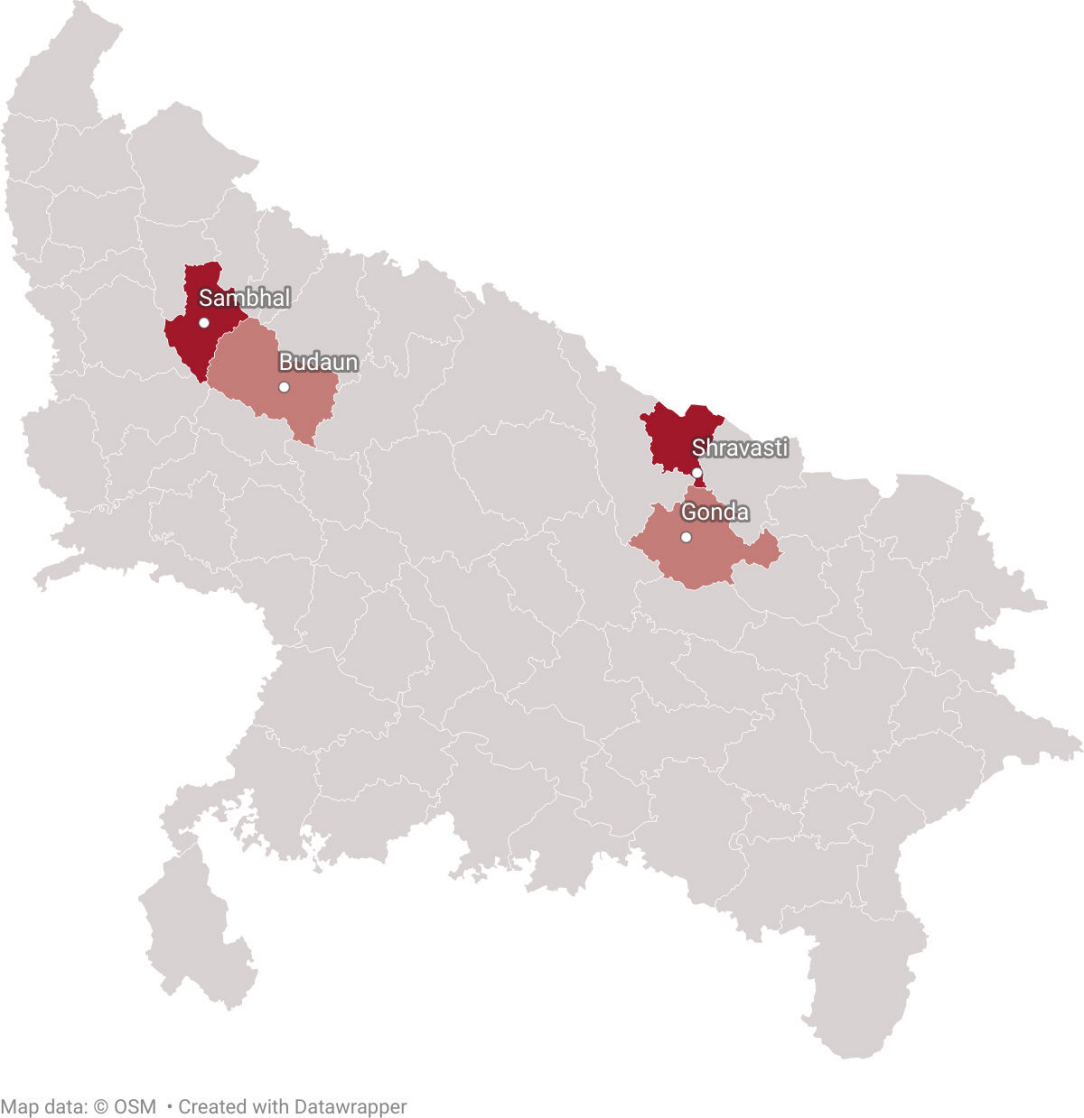
Intervention and control districts of Uttar Pradesh (adapted from OpenStreetMap data [36] using DataWrapper software [37], and published under Creative Commons Attribution-ShareAlike 2.0 Generic License [38]).

For this study, the government of UP has identified Shravasti and Sambhal as the intervention districts to implement the program. Keeping in view the 2-arm nature of the study, the study team has identified 2 usual care arm districts that closely match with the intervention districts using latest socio-demographic, antenatal, and immediate postpartum care indicators for the district (NFHS-5, 2019‐2021) [13]. Identified Gonda as the matching usual care arm district for Shravasti. The distance between these 2 districts is less than 50 km. Identified Budaun as matching usual care arm district to Sambhal. The distance between these 2 districts is less than 100 km. Table 1 provides sociodemographic and ANC and delivery practices in 4 study districts, and Figure 2 depicts the 4 study districts. The matched control districts were approved by the government of UP.

**Table 1.** Sociodemographic and health care–seeking profile of 4 study districts.

	Intervention arm districts		Usual care arm districts	
	Shravasti	Sambhal	Gonda	Budaun
Sociodemographic profile^a^				
Population (2011), n	1,117,361	2,199,774	3,433,919	3,681,896
Sex ratio of population, (females per 1000 males)	881	891	921	871
Urban population, %^b^	4	3	7	22
Tahsils, n	3	3	4	6
Villages, n	541	993	1817	2061
Literacy, %^b^	47	55	59	53
Scheduled caste population, %^b^	16.9	16.6	15.5	17.7
ANC^c^, delivery, and immediate postpartum care indicators (2019‐2021)^d^, %^e^				
Received at least 4 ANC visits	42.3	32.9	41.7	40.6
Mothers protected against tetanus toxoid	94.4	90.3	90.4	90.1
Mothers who consumed 100 iron-folic acid	16.6	21.3	17.2	15.9
Institutional births	80.4	74.2	81.8	72.3
Mothers who received postnatal care in 2 days	68.3	67.5	68.6	62.4
Mothers received MCP^f^ card	95.9	93.5	97.1	94.4

a Master data: Census 2011-state and district wise primary census abstract.

b These are census percentages.

c ANC: antenatal care.

d International Institute for Population Sciences (IIPS) and ICF. 2021. National Family Health Survey-5, India, 2019‐21: Uttar Pradesh. Mumbai: IIPS.

e Weighted percentages.

f MCP: Mother and Child Protection.

### Study Tools

Baseline and end line surveys will use qualitative methods for collecting data from the system stakeholders and quantitative methods for collecting data from the community, specifically the RDW. The data collection tools were pretested in Telangana in 2022-2023 and subsequently adapted to align with the programs and policies of UP. Table 2 provides details of respondents interviewed using qualitative data collection technique.

**Table 2.** Respondents, data collection method, sample size, and information areas of qualitative data collection tools.

Type of respondent and tool	Data collection method	Sample size	Who collects data?	Recruitment strategy	Key information areas of tool
Chief medical officer (Multimedia Appendix 1)	In-depth interview	1 per district	Qualitative researchers	Purposive	District policies and programs on maternal and newborn health Facilitators and barriers to implement IHRPTM^a^ program in district
Associate chief medical officer (Multimedia Appendix 2)	Semistructured interview	1 per district	Qualitative researchers	Purposive	Most prevalent HRPs^b^ in district Vulnerable areas or populations Issues with collection of RCH^c^, HMIS^d^, or HRP data Human resources availability
Specialist gynecologists (district hospital; Multimedia Appendix 3)	In-depth interview	1 per district	Qualitative researchers	Purposive	ANC^e^ case load at facility HRP case load at facility Volume of high-risk cases referred to facility Volume of late and unnecessary referrals Strategies for coping with referrals
Medical officers working in PHC^f^ and CHC^g^ (Multimedia Appendix 4)	Semistructured interview	4‐5 per district	Qualitative researchers	From 4 randomly selected PHCs of 2 blocks in a district	Support systems available for ensuring quality ANC Case load of ANC and HRPs Knowledge about 3 specific HRPs Management skills to care for women with 3 HRPs Knowledge about referral practices to woman with 3 HRPs
ANMs^h^ working in HSCs^i^ (Multimedia Appendix 5)	Semistructured interview	8‐10 per district	Qualitative researchers	2 ANMs per PHC, from above 4 randomly selected PHCs of 2 blocks in a district	Support systems available for ensuring quality ANC Case load of ANC and HRPs Knowledge about 3 specific HRPs Management skills to care woman with 3 HRPs Knowledge about referral practices to woman with 3 HRPs

a IHRPTM: Integrated High-Risk Pregnancy Tracking and Management.

b HRP: high-risk pregnancy.

c RCH: reproductive and child health.

d HMIS: health management information system.

e ANC: antenatal care.

f PHC: primary health center.

g CHC: community health center.

h ANM: auxiliary nurse midwives.

i HSC: health subcenter.

### Community-Level Data Collection

RDW who gave birth within the past year, regardless of HRP status, will be surveyed in all the 4 study districts, at baseline and at end line. Some unique features of the RDW tool are documentation of the previous pregnancy, including details of HRP condition (if applicable), as a story in mother’s own words; a picture of the Mother and Child Protection card to supplement with the question-specific data collected from the RDW through interview; ANC details; and HRP-specific detailed questions, where applicable (Table 3; Multimedia Appendix 6).

**Table 3.** Key data collected in the recently delivered women tool.

Data point collected	Details
Pregnancy and delivery details	Age, delivery date, type and place of delivery, outcome of delivery, child’s age, birth weight, term and preterm status, MCP^a^ card availability, and HRP^b^ status
Reproductive history	Children ever born and surviving, and pregnancy terminations
MCP card photo	With details of each ANC^c^ visit, in picture format
Story of recent pregnancy in mother’s own words	Investigators ask the mother to describe about her most recent pregnancy in her own words, from conception to delivery, including details of any HRP, if applicable. The story told by mother is documented here in a text format
ANC characteristics	Number of ANC visits, details of first ANC visit, experience of risk factors, TT^d^ and IFA^e^ usage, supplementary nutrition, and high-risk conditions experienced, including duration of problem and referrals made for the HRP
Delivery and newborn characteristics	Breech presentation, prolonged labor, excessive bleeding, facility stay after delivery, congenital anomalies, and reasons for cesarean section
Details about severe or moderate anemia, PIH^f^, APH^g^, and other HRP conditions experienced by mother in most recent pregnancy (if applicable)	Timing of first diagnosis of HRP and who diagnosed, risk factors associated with the HRP, and management, referral, counseling, and follow-up details related to the HRP condition
Satisfaction	Level of satisfaction with care received if HRP was present
Background characteristics	Literacy, schooling, religion, caste or tribe, occupation, and owning of own mobile phone by the mother

a MCP: Mother and Child Protection.

b HRP: high-risk pregnancy.

c ANC: antenatal care.

d TT: tetanus toxoid.

e IFA: iron-folic acid.

f PIH: pregnancy-induced hypertension.

g APH: antepartum hemorrhage.

### Sample Size of the Quantitative Surveys

The sample size of RDW to be included in the baseline and end line surveys was determined to generate indicators for each of the 3 HRP conditions (moderate or severe anemia, PIH, and APH) separately for the intervention and usual care arms. To achieve this, the sample size was estimated based on the HRP condition, with the lowest prevalence among the 3 conditions. This approach ensures that the sample size estimated for an HRP condition with the lowest prevalence will suffice for the HRP conditions with higher prevalence as well, using the most recent prevalence data for the HRP conditions among the pregnant women in India or North India.

From recent available literature in India, the prevalence of moderate or severe anemia among pregnant women in the north zone was 25% [39], PIH in India ranged from 5%‐17% [40], and prevalence of only APH is 2%‐5%, and placenta previa is 4%‐5% [41]. It implies, among the 3 HRP conditions, PIH has the lowest prevalence. Assuming the prevalence of PIH among RDW in the intervention arm and in the control arm, at baseline and end line surveys is 5%, the estimated sample size for PIH is not expected to differ by more or less than 1% (ie, 4%‐6% is the margin of error), with a 95% confidence level, the sample size of RDW required in both the intervention and in usual care arms, at baseline and at end line surveys is 1825 (rounded to 2000) [42]. It means we need to interview 2000 RDW in each arm, both at baseline and end line surveys to generate required sample size for each HRP condition. Assuming the above prevalence of HRPs holds true in the 2 study arms, by interviewing 2000 RDW cross-sectionally in each arm, we anticipate at least 400‐500 RDW with moderate or severe anemia, 120‐200 RDW with APH, placenta previa, or abruptio placenta, and 100‐340 women with PIH. Some of these RDW may have more than 1 of the 3 HRP conditions. Approximately 900‐1000 RDW in each arm will be without any of the 3 HRP conditions.

Given the low prevalence of PIH and APH (around 5%), with 1% margin of error, our study (2000 RDW per arm) is adequately powered at 95% (a conservative estimate). With a higher margin of error (say 1.5%), although the required sample size per arm will reduce to 821, the power of the study will fall below 95%, and the anticipated number of RDW with PIH and APH would be too small to compare (approximately 70‐130 per arm). This would result in high CIs, making HRP-specific impact evaluation a challenging task.

As different respondents will be surveyed at baseline and end line, there is no risk of data attrition. However, since HRP conditions data in this study is relying on self-reports by RDW and expected low prevalence of PIH and APH conditions (around 5%) in India, nonparticipation by RDW with these conditions could compromise the statistical power of the study. To overcome this problem, we have set a narrow margin of error (1%, ie, prevalence could range between 4%‐6%) for baseline and end line estimates in each arm. Our sample size calculation also accounts for a 10% nonresponse rate per arm at both baseline and end line. To increase the effective prevalence of the APH condition, the inclusion criteria for this condition encompass APH, placenta previa, and abruptio placenta. With recent estimates in India showing an increase in prevalence of PIH to 11% [43], while moderate or severe anemia was stagnant at around 26% [39], we are confident of including sufficient RDW with these 2 conditions. Nevertheless, if the prevalence of any of the 3 HRPs falls below 4% at baseline or end line in either arm, the statistical power of the study for that HRP condition will be compromised. We acknowledge this as a limitation of the study.

### Survey Design

A multistage sampling approach will be used to select RDW from each arm. As census data at the village and ward level is old (2011), the accredited social health activist (ASHA) jurisdiction will be used to serve as a primary sampling unit (PSU). A total of 50 PSUs per district (100 per arm) will be selected using a 2-stage sampling approach. During baseline, the 2023 list of RDW in an ASHA’s jurisdiction served as the basis for the selection of a PSU. Assuming an average PSU will have 20 RDW, we expect to cover 1000 RDW per district (2000 per am) at baseline and end line surveys.

As part of the first stage of sampling, 50 PSUs in each district were allocated according to the proportion of the block population of the district. As part of the second stage of sampling approach, the allocated number of PSUs in a block was selected using the probability proportionate to size sampling technique.

During the baseline, in a selected PSU, the list of women who were delivered in the past 1 year, regardless of delivery outcome, and the delivery data maintained by ASHA and anganwadi workers (AWWs) served as the basis for identifying the eligible respondents for survey. This list by ASHA and AWWs was cross-verified with the list available with the ANM of the respective PSU, and a comprehensive list of RDW to be approached for interview was prepared. By adopting this strategy, we eliminated the need for doing households listing exercise to identify eligible RDW in the selected PSUs.

In a sampled PSU, all the households with eligible respondents (RDW) were approached by female investigator or supervisor for consent or assent for a face-to-face interview (Multimedia Appendix 7). All consented RDW participants were interviewed by female investigators, mostly in RDW’s home. To ensure 100% enumeration of eligible respondents in a PSU, a “snowballing” technique was also used with interviewed RDW to check if any peer RDW in her neighborhood was missed by the ASHA, AWW, or ANM list. If any new RDW is found through the snowballing technique, she was interviewed, provided she belongs to the selected PSU. Table 4 provides the inclusion and exclusion criteria used for selecting the respondents for the baseline and end line surveys in the 4 study districts of UP.

**Table 4.** Inclusion exclusion criteria for respondents of baseline and end line surveys.

Respondent	Data collection method	Inclusion criteria	Exclusion criteria
RDW^a^	Personal interview	Woman aged 15‐49 years, have had delivery during the past 1 year from the date of survey Details of such woman are available with ASHA^b^, AWW^c^, and ANM^d^ in the sampled PSU^e^ or woman was identified through snowballing technique from an interviewed RDW RDW who is resident of nonsampled PSU but had delivery in sampled PSU in past 1 year and is willing to participate in survey	All those who do not give consent to participate in the survey RDW listed by ASHA, AWW, and ANM, but not available for interview on days of survey Had delivery just within 3 days of survey Maternal deaths and RDWs with severe illness and unable to participate in interview
District officials and Specialist gynecologists	IDI	Currently serving in the stated position in the survey district	With less than 1 year of experience in the current position, in the survey district Who do not give consent to participate in the survey
Medical officer and ANM	SSI	Currently serving in the stated position in the sampled PHC^f^ and HSC^g^	With less than 1 year of experience in the current position in sampled PHC and HSC Who do not give consent to participate

a RDW: recently delivered women.

b ASHA: accredited social health activist.

c AWW: anganwadi worker.

d ANM: auxiliary nurse midwife.

e PSU: primary sampling unit.

f PHC: primary health center.

g HSC: health subcenter.

Using the above-stated survey design, tools, and inclusion and exclusion criteria, data for the baseline survey component of the impact evaluation of the IHRPTM program were collected during June-October 2024. Baseline qualitative data from system stakeholders were collected by ARMMAN researchers from 4 districts during June-August 2024. Baseline quantitative data were collected from >4000 RDW by around 75 female investigators and supervisors, during September-October 2024, from 200 PSUs, spread across 4 districts and 2 arms.

### Ethical Considerations

#### Overview

Qualitative data at baseline and end line surveys will be collected by ARMMAN researchers using computer-assisted personal interview (CAPI) tools, while quantitative data will be outsourced to a professional data collection agency, with prior experience in collecting maternal and child health–related data in UP. At baseline, a 2-day meeting of the qualitative researchers was held to discuss specific nuances of each question of the qualitative tool, how to ensure uniformity while asking questions, distribution of qualitative data collection workload, etc. For the baseline RDW tool, the investigators and supervisors of the shortlisted agency were trained for 5 days by ARMMAN program’s research team on the following topics: IHRPTM program and its objectives, ethical practices to be adhered to while collecting data from RDW, how to and when to approach potential gatekeepers, which RDW are eligible for consent or assent, how to obtain consent or assent, how to collect data using CAPI technique; importance of privacy during data collection, confidentiality of data, data quality, and potential risks and mitigation measures. Baseline RDW data were collected by female investigators or supervisors using the CAPI tool. All the tools were translated into Hindi and back translated into English, to ensure accuracy and retain the meaning of questions and most of the questions in the background, ANC, and delivery care sections were tried and tested in other large-scale surveys like NFHS.

All listed RDW in a PSU were approached by a female investigator or supervisor for participation in the survey using the RDW tool (Multimedia Appendix 6). Only those RDWs who expressed interest to participate in the survey were interviewed after obtaining informed consent. Respondents who were literate and comfortable in reading the informed consent form were given the option to read the form themselves or the investigator read it aloud to them. Once informed consent is received, the survey began. If the respondent felt the time was not appropriate to participate in the survey, a convenient time was sought, and the investigator returned at the time specified by the respondent to conduct the interview. Prior to approaching RDW, the gatekeepers of RDW (village leaders, teachers, religious heads, household heads, etc) were sensitized about the study and the safety of the RDW participating in the study by supervisors. For minors, informed consent was attained from the adult guardian, with assent from the minor. While interviewing RDW with severe morbidities, the procedure involved the presence of a guardian during the interview. The informed consent was obtained from the participant in the presence of the guardian. If she is unable to answer or give a consent, the Mother and Child Protection card was copied with the consent of the guardian. Including severe HRP cases in the survey would provide us with specific signs and symptoms associated with severity of the HRP condition, including gaps in diagnosis and management of these cases. Excluding severe HRPs from survey preview may result in collecting data only from the mild or moderately severe cases, and such data are not representative of the actual situation on the ground; hence, we have instructed investigators to collect data from severe HRPs by adhering to stated ethical norms.

A few women with severe HRPs may get distressed during the interview. To handle distress, investigators were trained using real field examples in trauma-informed and empathetic interviewing techniques. This included training on how to create a conducive environment (private, quiet, and comfortable place) for interview. During moments of distress, investigators responded empathetically by saying things like, “I can understand the pain,” offering water or tissues or keeping silent for a while. Keeping in view the distress level (tearfulness, prolonged silence, nervousness, or withdrawal), the investigator stopped the interview until the RDW’s distress had fully subsided. The interview was resumed only when she indicated she was ready to continue.

#### Privacy

While collecting qualitative or quantitative data from the field, there will be situations when interviews cannot be conducted in complete privacy, as participants may be surrounded by other adult members or coworkers. During the training of data collection teams, the issue of privacy was discussed in detail, specifically focusing on two subtopics: (1) ways to minimize situations where the investigator is surrounded by other adults (starting the interview with generic issues like crops, yield, etc, till other adults move and the supervisor tries to move other adults to a separate location, etc) and (2) strategies (such as preinterview briefings about privacy, choosing alternate locations, and scheduling flexible times when fewer people are around) that can be adopted during data collection to move other people away from the interview site.

#### Confidentiality

During data collection, the respondent’s name will not be recorded whenever possible. In cases where names are collected during qualitative interviews, these will be converted into numeric codes (eg, ANM-1 and ANM-2) while presenting the results. At the time of in-depth interviews (IDIs) with district officials, although we record the name of the respondent on the hard copy of the tool, this is done solely to ensure we are interviewing the correct person. Identification details of these respondents will be anonymized once the transcription and translation of the IDIs are complete. IDIs were not audio recorded and were conducted in the presence of a notetaker. Identification details of IDIs were anonymized once the notes were converted into digital data.

During baseline, after completing the RDW interview, the interviewer saved and closed the file before leaving the interview site. Each interviewer was provided a password for sharing and uploading the completed files through email. A unique ID was assigned to each RDW to protect their identity. The uploaded datasets were downloaded centrally at the ARMMAN Hyderabad office on a password-protected computer. All electronic data were stored securely on a password-protected computer at ARMMAN until the completion of the end line survey. Access to identifiable information is restricted only to the research team. The data files, containing identifiers, will be stored separately from other data files and will be password-protected, with access restricted to the research team. To ensure confidentiality and secure data transfer or sharing, files containing identification details will be password-protected and encrypted. All hard copy records will be destroyed at the end of the project.

#### Data Storage and Sharing Policy

In accordance with the principles of transparency and reproducibility, all deidentified datasets (both qualitative and quantitative) will be securely stored on password-protected servers at ARMMAN for a minimum of 5 years following project completion. Thereafter, data will either be archived for secondary use or permanently destroyed. Only deidentified, aggregate-level data may be shared upon reasonable request from accredited researchers or institutions, contingent on prior approval from ARMMAN’s scientific review board and Sigma’s institutional review board (IRB), and in compliance with national data protection regulations.

#### Approvals

The study got approvals from ARMMAN’s scientific review board (017/2024) and Sigma, New Delhi’s IRB (10009/IRB/24‐25).

### Data Analysis

The qualitative data will be analyzed using Braun and Clarke’s [44] inductive thematic analysis framework. Interviews with system stakeholders (eg, chief MOs, associate chief MOs, MOs, and ANMs) conducted in Hindi and transcribed verbatim will be translated into English and back-translated to ensure both linguistic and conceptual accuracy. The 2 researchers will independently code the transcripts using ATLAS.ti (ATLAS.ti Scientific Software Development GmbH) or NVivo (Lumivero) software. The analysis will follow a structured process: (1) initial data familiarization, (2) development and iterative refinement of an inductive coding framework, (3) systematic application of codes, (4) and identification of emergent codes where necessary. Themes will be generated through synthesis and organization of the coded data. Anticipated themes include training and handholding of ANMs and MOs on HRPs, motivation and job satisfaction of ANMs and MOs, quality ANC, facilitators and barriers to HRP management, facility and community support to ANMs and MOs, workforce shortages, sociocultural barriers to ANC and HRP care, and demand for services. Interrater reliability will be ensured by having 2 researchers independently code 20% of the transcripts. Any discrepancies will be resolved through consensus. Thematic findings will be validated against existing literature and substantiated with verbatim participant quotations. This rigorous, transparent, and software-supported approach will ensure analytical robustness of the findings.

Using data validation checks, the quality of the quantitative data will be assessed for coverage, consistency, and completeness by ARMMAN researchers. The analysis of these data will be done using SPSS (version 21.0; IBM) or STATA (version 12.0; Stata Corp, LLC) software. Preliminary analysis includes frequency tables and cross-tabulations, while as part of advanced analysis, multivariate analysis techniques will be used. The cleaned data will be analyzed according to background characteristics; coverage and quality of the ANC, delivery, postnatal care; the outcome and output indicators; satisfaction with the care; etc. Advanced analysis will be carried out to quantify associations of the background characteristics with output and outcome indicators. As far as possible, quantitative results will be reported with effect estimates, 95% CIs, and significance levels.

We will use the DiD technique to estimate the causal effect of program interventions on outputs (eg, RDW sensitized and HRPs diagnosed early) and outcomes (eg, protocol-based management and referral of HRPs). This approach compares changes in outputs and outcomes over the intervention period (18 months) between the intervention and the usual care arms. For each quantitative output and outcome indicator summary, descriptive DiD or net change will provide causal effect of intervention on the indicator. The DiD-specific regression models will then be used to test whether interventions were significantly associated with changes in the outcomes and to assess the extent of changes relative to the control group.

To address the issue of missing data in the RDW dataset, before applying DiD, we will assess the extent and patterns of missingness—whether missing data are at baseline, end line, or both—and examine whether missingness is random or systematically related to intervention status or outcomes by comparing covariates and outcomes between complete and missing cases. Given the robust data quality assurance measures implemented during both surveys, we anticipate minimal missingness, and any missing data are expected to be missing at random. If the proportion of missing at random for any outcome or covariate exceeds 10%, we will apply appropriate imputation methods prior to fitting the DiD model. The DiD model will then include intervention and control status, baseline and end line survey indicators, and interaction terms between outcomes and covariates.

During the granular analysis of testing the association between background characteristics and output and outcome indicators, we will draw upon the WHO’s Social Determinants of Health framework [45]. Intersectionality between the background and confounding variables will be explored using subgroup analysis. In the multivariate regression model, predictor variables will only be included after testing for multicollinearity using variance inflation factor to ensure stability and reliability of the model.

### Triangulation of Mixed Methods Data

As part of the impact evaluation, one of the hypotheses being tested is “‘whether capacitated ANMs and MOs are able to translate their knowledge and skills to manage HRPs into practice.” This hypothesis will mainly be tested by triangulating both the datasets on the listed themes and indicators, particularly, during the end line (Table 5).

**Table 5.** How qualitative and quantitative datasets will be triangulated and integrated?

Theme or indicator	System level data (qualitative)	Community level data (quantitative)
Training and handholding of ANMs^a^ and MOs^b^ on HRPs^c^	Early diagnosis of HRPs reported by ANMs or MOs Improved knowledge and skills to manage HRPs by ANMs and MOs Improved confidence to manage HRPs	Proportion of HRPs diagnosed in the first trimester Protocol-based management of HRPs Protocol-based referral of HRPs Proportion of unnecessary or irrational referrals
Motivation and satisfaction of ANMs and MOs	Digital tools facilitating improved counseling and follow-up of women with HRPs by ANMs and MOs	Proportion of mothers with HRPs counseled and followed up by ANMs and MOs Proportion of mothers with HRPs highly satisfied with management and referral practices of ANMs and MOs
Quality ANC^d^	Improvement in quality of ANC services reported by ANMs and MOs	Proportion of mothers whose weight, hemoglobin, and blood pressure were collected at least 4 times in pregnancy Proportion of mothers who received complete ANC package of services
Facilitators and barriers to HRP management	Specific nuances of facilitators and barriers to HRP management reported by system stakeholders	Proportion of mothers with HRPs using public health facilities for management Proportion of mothers using public health facilities for ANC, delivery, and PNC^e^ care

a ANM: auxiliary nurse midwife.

b MO: medical officer.

c HRP: high-risk pregnancy.

d ANC: antenatal care.

e PNC: postnatal care.

To operationalize triangulation as a methodological practice, the study will use structured integration matrices, defined timelines, and clearly delineated roles. The process will take place during the end line analysis phase (March-June 2026), once both qualitative and quantitative datasets have been collected and validated.

An integration matrix (Table 5) has been developed to align qualitative themes (eg, training, handholding, barriers, and motivation) with corresponding quantitative indicators (eg, timely diagnosis, referral patterns, and satisfaction with care). Systematic side-by-side mapping of these themes will facilitate the comparison of convergence, complementarity, and divergence between system-level perspectives of HWs and community-level experiences of pregnant women.

The qualitative team will conduct thematic analysis, while the quantitative team will undertake statistical analyses, including regression and DiD. A joint synthesis committee comprising senior researchers, program investigators, and biostatisticians will meet regularly during the end line analysis phase to review the matrices, address discrepancies, and ensure methodological rigor.

The integrated findings will be presented as narratives and summary tables, clearly highlighting areas where the 2 data streams converge or diverge. This structured approach enhances transparency, strengthens the validity of results, and ensures that triangulation moves beyond the conceptual intent to systematically document the facilitators and barriers to translating the knowledge gained from the program into practice on the ground by the HWs.

### Use of Study Findings

Baseline study and impact evaluation findings will initially be shared with the government of UP, through Microsoft PowerPoint presentations and respective reports. The reports will also be shared with district officials of the 4 program districts for their review and consideration. Using data from baseline and end line surveys, ARMMAN will also develop research briefs on specific themes and look for opportunities to share these findings at various forums. If possible, the findings will also be disseminated through publication in scientific and peer-reviewed journals as well as through presentations at professional meetings and conferences. Once impact evaluation findings are available, ARMMAN plans to host meetings with state government officials and academicians to share relevant research findings, which could have implications for program modification when planning scale-up in other districts or states.

## Results

The impact evaluation protocol and the protocols for 6 HRP conditions were vetted by the government of UP in May-June 2024. In the 2 intervention districts of Sambhal and Shravasti, in June-July 2024, implementation of listed interventions started in a phased manner, and these interventions will continue for 18 months (until March 2026), in collaboration with district health officials. By November 2024, in the 2 intervention districts, the following interventions had been introduced: (1) protocols on 6 HRP conditions; (2) training of ANMs, MOs, specialist gynecologists, staff nurses, and community health officers on these protocols; and (3) a digital learning tool and a WhatsApp-based support system for ANMs. Baseline data from system stakeholders and community data from recently delivered women were collected during June-October 2024. From November 2024 to October 2025, the listed interventions will continue in the 2 intervention districts, and baseline data from both arms are being analyzed. By November 2024, all the ANMs, MOs, specialist gynecologists, staff nurses, and community health officers of 2 intervention districts were trained on 6 HRP protocols, the learning app for ANMs on the 6 HRPs, and the WhatsApp support system for real-time clarification of doubts by the ANMs (tech plus touch approach) was also introduced. The tracking and data integration system for improved tracking of the HRPs by health facilities at different levels of public health care system is yet to be introduced.

The proposed interventions, designed, developed, implemented, and evaluated using a tech plus touch approach, will capacitate HWs at different levels to make early diagnosis of the HRPs and provide protocol-based management of the diagnosed condition. The findings of the study will identify issues that require need-based planning to ensure quality ANC, delivery, and HRP care services by public health care system, and particularly the ANMs and MOs, who work at the grassroots. At the policy level, findings of the study will provide cues on facilitators and barriers to pregnancy care services at the block and district level, for necessary corrections. The study intends to make an important contribution by evaluating the usefulness and effectiveness of the proposed program and suggesting further improvements or modifications when scaled up across the entire state of UP, a region that is larger than many European countries.

## Discussion

### Overview

One of the major strengths of this study is that this study is being implemented in partnership with state and district health departments. Through our multicomponent interventions, the capacity of public health care system stakeholders (ANMs, MOs, and specialists) is being built to diagnose and manage HRP conditions at their respective facilities. If ANMs and MOs manage HRPs at their level, there will be a reduction in unnecessary or late referrals to tertiary or higher-level facilities, allowing these facilities to concentrate mainly on the most critical cases. The study will establish a robust system for not only managing HRPs but for ensuring quality ANC services and continuum of care in geographies with poor maternal outcomes (Table 1). If the program is successful in these areas, it will pave the way for future implementation efforts in other states.

The proposed study anticipates a few limitations, such as delays in getting permissions to collect data from ANM, MOs, specialists, and RDW from the 2 control districts. In intervention districts, many MOs have recently joined the service, and their focus is on preparing for higher studies. Therefore, they may not be very keen to participate in this program. Also, if they get admission in higher studies, they will leave the service, and refilling the position may take time. We are also concerned about the support for the program from system stakeholders who are nearing retirement and working on a contractual basis. The end line qualitative results, particularly from the 2 intervention districts, might be influenced by social desirability bias, as by then, the majority of the system stakeholders will be aware of the purpose of the program and its objectives. To minimize the social desirability bias, we will do data collection methods triangulation and information triangulation (collecting same data from multiple question formats). To minimize the bias, we will also triangulate both qualitative and quantitative data, wherever feasible. Frequent transfers of district and state officials may also create hurdles in the timely implementation of interventions and getting approvals for implementing interventions on the ground.

### Conclusions

The study will test the hypothesis of whether an integrated, comprehensive, system-driven intervention for improved identification, tracking, and end-to-end management of HRPs is feasible in geographies with poor maternal outcomes or not. At the community level, the study will provide insights on the role of sociodemographic, as well as ANC and delivery factors, that influence HRP management and referral practices. This trial will provide valuable insights into the feasibility and effectiveness of the program, at system and community levels, in low-resource settings like UP. If successful, these insights can feed into capacitating HWs, at scale, in all the districts on diagnosis and management of HRPs, with significant potential for improving maternal and neonatal outcomes of the state. We will also explore the options for scaling the program to include conditions beyond the 6 HRPs in other districts of UP.

For this study, the Transparent Reporting of Evaluations with Nonrandomized Designs (TREND) statement was followed [46] to ensure transparent and comprehensive reporting (Multimedia Appendix 8). Our protocol adheres to around 20 of the 23 items in the TREND checklist.

## Supplementary material

10.2196/74993 Multimedia Appendix 1 Chief Medical Officer Questionnaire.

10.2196/74993 Multimedia Appendix 2 Semistructured interview with Associate Chief medical officer

10.2196/74993 Multimedia Appendix 3 In-depth interview with specialist.

10.2196/74993 Multimedia Appendix 4 Medical officer.

10.2196/74993 Multimedia Appendix 5 Auxiliary nurse midwife tool.

10.2196/74993 Multimedia Appendix 6 Recently delivered women tool.

10.2196/74993 Multimedia Appendix 7 For recently delivered women - Consent or Assent form.

10.2196/74993 Multimedia Appendix 8 TREND checklist for evaluating Integrated High-Risk Pregnancy Tracking and Management program's impact on outcomes.

## Abbreviations

ANC: antenatal care
ANM: auxiliary nurse midwife
AP: Andhra Pradesh
APH: antepartum hemorrhage
ARMMAN: Advancing Reduction in Mortality and Morbidity of Mothers, Children, and Neonates
ASHA: accredited social health activist
AWW: anganwadi worker
BEmONC: basic emergency obstetric and newborn care
CAPI: computer-assisted personal interview
CEmONC: comprehensive emergency obstetric and newborn care
CHC: community health center
DiD: difference-in-difference
EPMM: Ending Preventable Maternal Mortality
HRP: high-risk pregnancy
HSC: health subcenter
HW: health worker
IDI: in-depth interview
IFA: iron-folic acid
IHRPTM: Integrated High-Risk Pregnancy Tracking and Management
IRB: institutional review board
MO: medical officer
NFHS: National Family Health Survey
NGO: nongovernmental organization
PHC: primary health center
PIH: pregnancy-induced hypertension
PSU: primary sampling unit
QANC: quality antenatal care
RCH: Reproductive and Child Health
RDW: recently delivered women
TREND: Transparent Reporting of Evaluations with Nonrandomized Designs
UP: Uttar Pradesh
WHO: World Health Organization
