# Supplementary material for: Impact of a Multicomponent Intervention to Build Capacity of Public Health Workers to Make Algorithmic Diagnosis and Management of High-Risk Pregnancies in Uttar Pradesh, India: Protocol for a Matched-Control, Before-After, Quasi-Experimental Study With a Mixed Methods Design
Source: JMIR Res Protoc. 2025 Dec 9;14:e74993. doi: 10.2196/74993 (PMC12690279; doi:10.2196/74993)
Supplement: Multimedia Appendix 1 [file resprot-v14-e74993-s001.docx]

**Annexure 1: Informed consent forms and tools (IDIs) used for the situational assessment**

**अनुलग्नक 1: सिचवेशनल असेसमेंट के लिए इस्‍तेमाल किया जाने वाला सूचित सहमति फॉर्म और टूल्‍स (आईडीआई)**

**Informed Consent Form– 1**

**सूचित सहमति फॉर्म–1**

[For senior officials at the district level

[ जिला स्‍तर पर सीनियर ऑफिसर

Dear Sir/Madam

डियर सर/मैडम

**Introduction:** Greetings. I am _______________ (name), from ARMMAN. ARMMAN is an India based not-for-profit organization that leverages technology to create scalable solutions empowering mothers and enabling healthy children. ARMMAN is committed to improving the well-being of pregnant women, mothers and children in the first 5 (five) years of their life.

परिचय: नमस्‍ते। मैं............(नाम) अरमान से हूँ, अरमान एक भारत आधारित गैर-लाभकारी संगठन है जो माताओं को सशक्‍त बनाने और स्‍वस्‍थ्‍य बच्‍चे पैदा करने में स्‍केलेब समाधान बनाने के लिए तकनीकी का इस्‍तेमाल करता है। अरमान गर्भवती महिलाओं, उनके जीवन के पहले 5 सालों में माताओं और बच्‍चों के कल्‍याण में सुधार करने के लिए प्रतिबद्ध है।

I will provide detailed information about the study in just a few minutes. But before I do that, I want to give you a short summary to help you decide if you want to take part in this study. You need to know:

हम बस कुछ मिनटों में मैं स्‍टडी के बारे में विस्‍तृत जानकारी प्रदान करूंगा। लेकिन इससे पहले कि मैं ऐसा करूं, मैं यह तय करने में आपकी मदद के लिए एक छोटा सारांश देना चाहूंगा कि क्‍या आप इस स्‍टडी में भाग लेना चाहेंगे। आपको पता होना चाहिए:

1. This research is being done to understand systems and its stakeholder’s readiness to diagnose & manage high-risk pregnancies in the district.

यह रिसर्च को जिलो में उच्‍च जोखिम वाली गर्भावस्‍था का पता लगाने और प्रबंधित करने की सिस्‍टम्‍ और हितधारकों की तैयार को समझने के लिए किया जा रहा है।

1. Whether you take part is your decision.

क्‍या आप भाग लेने है यह आपका फैसला है।

1. You do not have to take part;

आपको भाग नहीं लेना है;

1. You can change your mind at any time;

आप किसी भी समय अपना मन बदल सकते हैं;

1. Your decision will not be held against you by ARMMAN or anyone else;

आपका फैसला अरमान या किसी अन्य द्वारा आपके खिलाफ नहीं माना जाएगा;

1. If you take part, you will be asked to participate in a survey which will take around 45 to 60 minutes.

यदि आप भाग लेते हैं, तो आपसे सर्वे में भाग लेने के लिए कहा जाएगा जिसमें 45 से 60 मिनट का समय लगेगा।

1. You will not benefit directly from taking part, but we hope to be able to help others in the future.

आपको भाग लेने से सीधे तौर पर कोई लाभ नहीं मिलेगा, लेकिन हम भविष्‍य में दूसरों की मदद करने में सक्षम होने की उम्‍मीद करते हैं।

1. The primary risk to you if you take part is that others may find out the information you’ve shared, but we will try not to let this happen.

यदि आप इसमें भाग लेती हैं तो आपके लिए प्रमुख जोखिम यह है कि अन्य लोगों को आपके द्वारा शेयर की गई जानकारी मिल सकती है, लेकिन हम ऐसा न होने देने की कोशिश करेंगे।

**Purpose:** ARMMAN in partnership with the Health and Family Welfare Department, the Government of Uttar Pradesh is implementing the High-Risk Pregnancy Tracking & Management (IHRPTM) program in two intervention and two control districts of UP. We are initiating the online capacity building program designed based on the High-risk Pregnancy Management guidelines developed for the ANMs, MOs, SNs and specialists for six high-risk pregnancy (HRP) conditions based on the prevailing conditions and suggestions from experts in Uttar Pradesh. We are preparing to implement the technological platform to strengthen the health system pertinent to maternal and child health care by developing an app for additional support to ANMs and MOs apart from training and an app developed to track high-risk pregnancies and integrated with the RCH portal. This will enable the availability of women’s pregnancy-related information accessible at levels of health care providers providing services to plan, prepare and provide quality services to antenatal women.

**उद्देश्‍य:** उत्तर प्रदेश सरकार के स्वास्थ्य एवं परिवार कल्याण विभाग, उत्तर प्रदेश के साथ साझेदारी में अरमान, प्रदेश के दो हस्तक्षेप और दो कंट्रोल डिस्ट्रिक्‍ट में उच्च जोखिम वाली गर्भावस्था की ट्रैकिंग और प्रबंधन (आईएचआरपीटीएम) कार्यक्रम को लागू कर रहा है। हम उत्तर प्रदेश में मौजूदा परिस्थितियों और विशेषज्ञों के सुझावों के आधार पर छह उच्च जोखिम वाली गर्भावस्था (एचआरपी) स्थितियों के लिए एएनएम, एमओ, एसएन और विशेषज्ञों के लिए विकसित उच्च जोखिम वाली गर्भावस्था के प्रबंधन के दिशानिर्देशों के आधार पर ऑनलाइन क्षमता निर्माण कार्यक्रम शुरू कर रहे हैं। हम प्रशिक्षण के अलावा, उच्च जोखिम वाली गर्भावस्थाओं को ट्रैक करने और आरसीएच पोर्टल के साथ एकीकृत करने के लिए एक ऐप बनाने के अलावा एएनएम और एमओ को अतिरिक्त सहायता प्रदान करने के लिए एक ऐप तैयार करके मातृ एवं शिशु स्वास्थ्य देखभाल से संबंधित स्वास्थ्य प्रणाली को मजबूत करने के लिए तकनीकी प्लेटफॉर्म को लागू करने की तैयारी कर रहे हैं। इससे स्वास्थ्य देखभाल प्रदाताओं के स्तर पर महिलाओं की गर्भावस्था से संबंधित जानकारी उपलब्ध हो सकेगी, जिससे वे प्रसवपूर्व महिलाओं के लिए योजना तैयार कर सकेंगे, तैयारी कर सकेंगे और गुणवत्तापूर्ण सेवाएं प्रदान कर सकेंगे।

**Risks & Benefits:** A possible risk to taking part in this study is that people outside the research team may find out your answers to the questions. We try and make it so this will not happen, and you will not be identified by your name or designation. Your shared insights will help in the design, conceptualisation of the indicators for monitoring and evaluation, and implementation of the IHRPTM program in UP.

**जोखिम और फायदे:** इस स्‍टडी में भाग लेने का एक संभावित जोखिम यह है कि रिसर्च टीम के बाहर के लोग आपके सवालों के जवाब जान सकते हैं। हम कोशिश करते हैं कि ऐसा न हो और आपके नाम या पद से आपकी पहचान ना की जा सके पहचाना जाए। आपके द्वारा शेयर की गई जानकारी यूपी में आईएचआरपीअीएम प्रोग्राम की मॉनिटरिंग और मूल्‍यांकन और कार्यान्‍वयन के लिए संकेतकों को तैयार करने और अवधारणा बनाने में मदद करेगी।

**Confidentiality:** We will do everything we can to keep the information you share is secret. All the information you share will not be identified with your name or designation. This interview shall take place in private and it shall take 45-60 minutes.

**गोपनीयता:** हम वह सभी चीजें करेंगे जो हम आपके द्वारा शेयर की गई जानकारी को गोपनीय रखने के लिए कर सकते हैं। आपके द्वारा शेयर की गई सभी जानकारी को आपके नाम या पद के साथ पहचाना नहीं जा सकेगा। यह इंटरव्‍यू प्राइवेट स्‍थान पर किया जाता है और इस इंटरव्‍यू में 45-60 मिनट का समय लगेगा।

**Recording:** I would also like to record this interview so that any information provided by you is not missed out.

**रिकॉर्डिंग:** मैं इस इंटरव्‍यू को रिकॉर्ड भी करना चाहूंगा ताकि आपके द्वारा प्रदान की गई कोई भी जानकारी छूट ना जाए।

**Oral consent and recording:** I have read the consent form. I understand that I am being asked to take part in the IHRPTM research study. I understand I can keep a copy of this form if I want so that I can review later, contact someone about the study, or keep for my records.

**मौखिक सहमति और रिकॉर्डिंग:** मैंने सहमति फॉर्म को पढ़ लिया है। मैं समझता हूँ कि मुझसे आईएचआरपीटीएम रिसर्च स्‍टडी में भाग लेने के लिए कहा जाता रहा है। मैं समझता हूँ कि मैं अगर मैं चाहूं तो इस फॉर्म की एक कॉपी रख सकता हूँ जिसे मैं बाद में देख सकता हूँ, स्‍टडी के बारे में किसी से संपर्क कर सकता हूँ, या अपने रिकॉर्ड के लिए रख सकता हूँ।

I consent to take part in this study and provide permission to record my interview. I understand that if I want to stop taking part I may do so at any time.

मैं इस स्‍टडी में भाग लेने के लिए सहमति देता हूँ और मेरे इंटरव्‍यू को रिकॉर्ड करने की अनुमति देता हूँ।

Do you consent to participate in this study? YES NO

क्‍या आप इस स्‍टडी में भाग लेने के लिए सहमति देते हैं? हां नहीं

Do you consent to record the interview? YES NO

क्‍या आप इंटरव्‍यू को रिकॉर्ड करने के लिए सहमति देते हैं? हां नहीं

Do you agree for note taking of your interview by a separate note taker? YES NO

Please let me know if you would like to keep a copy of this form so that you can review the information at a later date, contact someone about the study, or keep it for your records.

**कृपया मुझे बताएं कि क्‍या आप इस फॉर्म की कॉपी रखना चाहेंगे ताकि आप बाद की तारीख में जानकारी को देख सकें, स्‍टडी के बारे में किसी से संपर्क कर सकें, और इसे आपके रिकॉर्ड के लिए रख सकें।**

Name of the Interviewer: ____________________

इंटरव्‍यूअर का नाम:

Date: __________________

तारीख:

**Contact** If you have any questions or concerns regarding this interview, please connect with: ARMMAN contact: Dr. Hanimi Reddy Modugu , Email: hanimi@armman.org, Mobile: +91 99118 22445

**संपर्क** यदि आपके पास इस इंटरव्‍यू के संबंध में कोई प्रश्‍न या चिंताएं हैं, तो कृपया निम्‍न से संपर्क करें: अरमान का कॉन्‍टेक्‍ट: डॉ. हनिमी रेड्डी मोडुगु, ईमेल: hanimi@armman.org, मोबाइल: +91 99118 22445

If you have any concerns regarding your rights as a participant, please contact Sigma Research and Consulting (IRB) at [irb.sigma@sigma-india.in](mailto:irb.sigma@sigma-india.in), Phone: 011- 41063450

Note: Please let us know if we can quote your participation/attribution according to your designation as we would you like to quote your participation?____________________________________________

**Interview guide with District officials: DMHO, DEO, DPHNO, Superintendents, etc.**

*[Record details of other in-charge positions held, Division representing, education qualification, years of service, Years of service in current position]*

1. How severe is the problem of high-risk pregnancy during the antenatal period and its outcomes in your district?

आपके जिले में प्रसवपूर्व अवधि के दौरान उच्च जोखिम वाली गर्भावस्था की समस्या कितनी गंभीर है और इसके क्‍या परिणाम होते हैं?

2. In your opinion, what is the cause of these high-risk pregnancies?

आपकी राय में, इन उच्च जोखिम वाली गर्भधारणाओं का कारण क्या है?

3. What are the current factors affecting the tackling of high-risk pregnancies during the antenatal period? *(Health infrastructure, human resources, cultural factors, quality of services, knowledge on high-risk management at health care staff level, referral practices, socio-cultural practices, environmental conditions, geography, other pre-existing health conditions)*

प्रसवपूर्व अवधि के दौरान उच्च जोखिम वाली गर्भावस्थाओं से निपटने को प्रभावित करने वाले मौजूदा कारक कौन से हैं? (हेल्‍थ इन्‍फ्रास्‍ट्रक्‍चर, मानव संसाधन, सांस्कृतिक कारक, सेवाओं की क्वालिटी, हेल्‍थ केयर स्टाफ स्तर पर उच्च जोखिम के प्रबंधन पर ज्ञान, रेफरल प्रथाएं, सामाजिक-सांस्कृतिक प्रथाएं, पर्यावरणीय स्थितियां, भूगोल, अन्य पहले से मौजूद स्‍वास्‍थ्‍य समस्‍याएं)

4. Are there any other initiatives being taken to address high-risk pregnancy management during the antenatal period within the district?

क्या जिले में प्रसवपूर्व अवधि के दौरान उच्च जोखिम वाली गर्भावस्था प्रबंधन के लिए कोई अन्य पहल की जा रही है?

5. What do you think about the online capacity building based on the new High risk pregnancy guideline for specialists, ANMs, and MOs? Do you think it will help in tackling the current situation more effectively? How?

आप विशेषज्ञों, एएनएम और एमओ के लिए नए उच्च जोखिम गर्भावस्था दिशा-निर्देश के आधार पर ऑनलाइन क्षमता निर्माण के बारे में क्या सोचते हैं? क्या आपको लगता है कि यह मौजूदा स्थिति को अधिक प्रभावी ढंग से संभालने में मदद करेगा? कैसे?

6. What is your view on developing a sound supporting technology for managing and tracking high pregnancies, providing support through apps for the ANMs to advise pregnant women where to go and what to do based on individual patient history?

उच्च जोखिम वाली गर्भावस्‍था के प्रबंधन और ट्रैकिंग करने, तथा एएनएम द्वारा गर्भवती महिलाओं को सलाह देने के लिए ऐप्स के माध्यम से सहायता प्रदान करने, अलग-अलग मरीज के इतिहास के आधार पर बताने कि कहां जाना है क्‍या करना है, एक साउंड सपोर्ट टेक्‍नोलॉजी तैयार करने के बारे में आपके क्‍या विचार करें।

7. What are the current indicators within the MCH being reviewed every month? What MCH indicators are a cause of concern currently?

एचसीएच के अंदर मौजूदा संकेतक कौन से हैं जिनकी हम महीने समीक्षा की जाती है? कौन के एमसीएच संकेत इस समय चिंता का कारण हैं?

8. Are there other new initiatives and areas of growth available to improve MCH program performance? (Scope of technology in improving data collection, management and tracking pregnancies, referrals)

क्या एमसीएच प्रोग्राम के प्रदर्शन को बेहतर बनाने के लिए अन्य नई पहलें और विकास के क्षेत्र उपलब्ध हैं? (डेटा कलेक्‍शन, मैनेजमेंट और गर्भावस्था पर नज़र रखने, रेफरल में सुधार करने में टेक्‍नोलॉजी का दायरा)

1. What changes do you expect (in terms of indicators) post capacity building of health workers during the antenatal period?

प्रसवपूर्व अवधि के दौरान हेल्‍थ वर्कर्स की क्षमता निर्माण के बाद आप (संकेतकों के संदर्भ में) क्या परिवर्तन की उम्‍मीद करते हैं?

1. According to you how important is it for the district to address the issue of high-risk pregnancies? What impact does it have on the overall health system?

आपके अनुसार उच्‍च जोखिम वाली गर्भावस्‍था की समस्‍या का समाधान करने के लिए जिले के लिए क्‍या महत्‍वपूर्ण है? कुलमिलाकर हेल्‍थ सिस्‍टम पर इसका क्‍या प्रभाव पड़ता है?
