# Supplementary material for: Impact of a Multicomponent Intervention to Build Capacity of Public Health Workers to Make Algorithmic Diagnosis and Management of High-Risk Pregnancies in Uttar Pradesh, India: Protocol for a Matched-Control, Before-After, Quasi-Experimental Study With a Mixed Methods Design
Source: JMIR Res Protoc. 2025 Dec 9;14:e74993. doi: 10.2196/74993 (PMC12690279; doi:10.2196/74993)
Supplement: Multimedia Appendix 2 [file resprot-v14-e74993-s002.docx]

**Annexure 2**

**Informed consent– For Semi structured Interview with PO-MCH/DEO/Others**

*[For senior officials at the district level – stakeholder analysis]*

*सूचित सहमति***– पीओ-एमसीएच/डीईओ/अन्‍य के साथ स्‍ट्रक्‍चर्ड इंटरव्‍यू के लिए**

[ जिला स्‍तर पर सीनियर ऑफिसर-हितधारक का विश्‍लेषण*]*

Dear Sir/Madam

डियर सर/मैडम

Greetings.

नमस्‍ते

**Introduction:** Greetings. I am _______________ (name), from ARMMAN. ARMMAN is an India based not-for-profit organization that leverages technology to create scalable solutions empowering mothers and enabling healthy children. ARMMAN is committed to improving the well-being of pregnant women, mothers and children in the first 5 (five) years of their life.
परिचय: नमस्‍ते। मैं............(नाम) अरमान से हूँ, अरमान एक भारत आधारित गैर-लाभकारी संगठन है जो माताओं को सशक्‍त बनाने और स्‍वस्‍थ्‍य बच्‍चे पैदा करने में स्‍केलेब समाधान बनाने के लिए तकनीकी का इस्‍तेमाल करता है। अरमान गर्भवती महिलाओं, उनके जीवन के पहले 5 सालों में माताओं और बच्‍चों के कल्‍याण में सुधार करने के लिए प्रतिबद्ध है।

I will provide detailed information about the study in just a few minutes. But before I do that, I want to give you a short summary to help you decide if you want to take part in this study. You need to know:

हम बस कुछ मिनटों में मैं स्‍टडी के बारे में विस्‍तृत जानकारी प्रदान करूंगा। लेकिन इससे पहले कि मैं ऐसा करूं, मैं यह तय करने में आपकी मदद के लिए एक छोटा सारांश देना चाहूंगा कि क्‍या आप इस स्‍टडी में भाग लेना चाहेंगे। आपको पता होना चाहिए:

1. This research is being done to understand systems and its stakeholder’s readiness to diagnose & manage high-risk pregnancies in the district.

यह रिसर्च को जिलो में उच्‍च जोखिम वाली गर्भावस्‍था का पता लगाने और प्रबंधित करने की सिस्‍टम्‍ और हितधारकों की तैयार को समझने के लिए किया जा रहा है।

1. Whether you take part is your decision.

क्‍या आप भाग लेने है यह आपका फैसला है।

1. You do not have to take part;

आपको भाग नहीं लेना है;

1. You can change your mind at any time;

आप किसी भी समय अपना मन बदल सकते हैं;

1. Your decision will not be held against you by ARMMAN or anyone else;

आपका फैसला अरमान या किसी अन्य द्वारा आपके खिलाफ नहीं माना जाएगा;

1. If you take part, you will be asked to participate in a survey which will take around 45 to 60 minutes.

यदि आप भाग लेते हैं, तो आपसे सर्वे में भाग लेने के लिए कहा जाएगा जिसमें 45 से 60 मिनट का समय लगेगा।

1. You will not benefit directly from taking part, but we hope to be able to help others in the future.

आपको भाग लेने से सीधे तौर पर कोई लाभ नहीं मिलेगा, लेकिन हम भविष्‍य में दूसरों की मदद करने में सक्षम होने की उम्‍मीद करते हैं।

1. The primary risk to you if you take part is that others may find out the information you’ve shared, but we will try not to let this happen.

यदि आप इसमें भाग लेती हैं तो आपके लिए प्रमुख जोखिम यह है कि अन्य लोगों को आपके द्वारा शेयर की गई जानकारी मिल सकती है, लेकिन हम ऐसा न होने देने की कोशिश करेंगे।

**Purpose:** ARMMAN in partnership with the Health and Family Welfare Department, the Government of Uttar Pradesh is implementing the High-Risk Pregnancy Tracking & Management (IHRPTM) program in two intervention and two control districts of UP. We are initiating the online capacity building program designed based on the High-risk Pregnancy Management guidelines developed for the ANMs, MOs, SNs and specialists for six high-risk pregnancy (HRP) conditions based on the prevailing conditions and suggestions from experts in Uttar Pradesh. We are preparing to implement the technological platform to strengthen the health system pertinent to maternal and child health care by developing an app for additional support to ANMs and MOs apart from training and an app developed to track high-risk pregnancies and integrated with the RCH portal. This will enable the availability of women’s pregnancy-related information accessible at levels of health care providers providing services to plan, prepare and provide quality services to antenatal women.

**उद्देश्‍य:** उत्तर प्रदेश सरकार के स्वास्थ्य एवं परिवार कल्याण विभाग, उत्तर प्रदेश के साथ साझेदारी में अरमान, प्रदेश के दो हस्तक्षेप और दो कंट्रोल डिस्ट्रिक्‍ट में उच्च जोखिम वाली गर्भावस्था की ट्रैकिंग और प्रबंधन (आईएचआरपीटीएम) कार्यक्रम को लागू कर रहा है। हम उत्तर प्रदेश में मौजूदा परिस्थितियों और विशेषज्ञों के सुझावों के आधार पर छह उच्च जोखिम वाली गर्भावस्था (एचआरपी) स्थितियों के लिए एएनएम, एमओ, एसएन और विशेषज्ञों के लिए विकसित उच्च जोखिम वाली गर्भावस्था के प्रबंधन के दिशानिर्देशों के आधार पर ऑनलाइन क्षमता निर्माण कार्यक्रम शुरू कर रहे हैं। हम प्रशिक्षण के अलावा, उच्च जोखिम वाली गर्भावस्थाओं को ट्रैक करने और आरसीएच पोर्टल के साथ एकीकृत करने के लिए एक ऐप बनाने के अलावा एएनएम और एमओ को अतिरिक्त सहायता प्रदान करने के लिए एक ऐप तैयार करके मातृ एवं शिशु स्वास्थ्य देखभाल से संबंधित स्वास्थ्य प्रणाली को मजबूत करने के लिए तकनीकी प्लेटफॉर्म को लागू करने की तैयारी कर रहे हैं। इससे स्वास्थ्य देखभाल प्रदाताओं के स्तर पर महिलाओं की गर्भावस्था से संबंधित जानकारी उपलब्ध हो सकेगी, जिससे वे प्रसवपूर्व महिलाओं के लिए योजना तैयार कर सकेंगे, तैयारी कर सकेंगे और गुणवत्तापूर्ण सेवाएं प्रदान कर सकेंगे।

**Risks & Benefits:** A possible risk to taking part in this study is that people outside the research team may find out your answers to the questions. We try and make it so this will not happen, and you will not be identified by your name or designation. Your shared insights will help in the design, conceptualisation of the indicators for monitoring and evaluation, and implementation of the IHRPTM program in UP.

**जोखिम और फायदे:** इस स्‍टडी में भाग लेने का एक संभावित जोखिम यह है कि रिसर्च टीम के बाहर के लोग आपके सवालों के जवाब जान सकते हैं। हम कोशिश करते हैं कि ऐसा न हो और आपके नाम या पद से आपकी पहचान ना की जा सके पहचाना जाए। आपके द्वारा शेयर की गई जानकारी यूपी में आईएचआरपीअीएम प्रोग्राम की मॉनिटरिंग और मूल्‍यांकन और कार्यान्‍वयन के लिए संकेतकों की तैयार करने और अवधारणा बनाने में मदद करेगी।

**Confidentiality:** We will do everything we can to keep the information you share is secret. All the information you share will not be identified with your name or designation. This interview shall take place in private and it shall take around an hour.

**गोपनीयता:** हम वह सभी चीजें करेंगे जो हम आपके द्वारा शेयर की गई जानकारी को गोपनीय रखने के लिए कर सकते हैं। आपके द्वारा शेयर की गई सभी जानकारी को आपके नाम या पद के साथ पहचाना नहीं जा सकेगा। यह इंटरव्‍यू प्राइवेट स्‍थान पर किया जाता है और इस इंटरव्‍यू में 1 घंटे का समय लगेगा।

**Oral consent:** I have read the consent form. I understand that I am being asked to take part in the IHRPTM research study. I understand I can keep a copy of this form if I want so that I can review later, contact someone about the study, or keep for my records.

**मौखिक सहमति:** मैंने सहमति फॉर्म को पढ़ लिया है। मैं समझता हूँ कि मुझसे आईएचआरपीटीएम रिसर्च स्‍टडी में भाग लेने के लिए कहा जाता रहा है। मैं समझता हूँ कि मैं अगर मैं चाहूं तो इस फॉर्म की एक कॉपी रख सकता हूँ जिसे मैं बाद में देख सकता हूँ, स्‍टडी के बारे में किसी से संपर्क कर सकता हूँ, या अपने रिकॉर्ड के लिए रख सकता हूँ।

I consent to take part in this study and provide permission to record my interview. I understand that if I want to stop taking part I may do so at any time.

मैं इस स्‍टडी में भाग लेने के लिए सहमति देता हूँ और मेरे इंटरव्‍यू को रिकॉर्ड करने की अनुमति देता हूँ।

Do you consent to participate in this study? YES NO

क्‍या आप इस स्‍टडी में भाग लेने के लिए सहमति देते हैं? हां नहीं

Please let me know if you would like to keep a copy of this form so that you can review the information at a later date, contact someone about the study, or keep it for your records.

**कृपया मुझे बताएं कि क्‍या आप इस फॉर्म की कॉपी रखना चाहेंगे ताकि आप बाद की तारीख में जानकारी को देख सकें, स्‍टडी के बारे में किसी से संपर्क कर सकें, और इसे आपके रिकॉर्ड के लिए रख सकें।**

Name of the Interviewer: ____________________

इंटरव्‍यूअर का नाम:

Date: __________________

तारीख:

**Contact** If you have any questions or concerns regarding this interview, please connect with: ARMMAN contact: Dr. Hanimi Reddy Modugu , Email: hanimi@armman.org, Mobile: +91 99118 22445

If you have any concerns regarding your rights as a participant, please contact Sigma Research and Consulting (IRB) at [irb.sigma@sigma-india.in](mailto:irb.sigma@sigma-india.in), Phone: 011- 41063450

**संपर्क** यदि आपके पास इस इंटरव्‍यू के संबंध में कोई प्रश्‍न या चिंताएं हैं, तो कृपया निम्‍न से संपर्क करें: अरमान का कॉन्‍टेक्‍ट: डॉ. हनिमी रेड्डी मोडुगु, ईमेल: hanimi@armman.org, मोबाइल: +91 99118 22445

**Semi-structured questionnaire for district-level officials**

**जिला स्‍तर के अधिकारियो के लिए सेमी-स्‍ट्रक्‍चर्ड क्‍वेश्‍चनेयर**

**Background information**

**बैकग्राउंड की जानकारी**

Educational qualification:

शौक्षिक योग्‍यता:

Designation:

पद:

Choose the District currently serving:

उस जिलो को चुनें जिसे आप इस समय सेवाएं प्रदान कर रहे हैं:

Choose the Block currently serving: NA/If applicable mention ____________________

उस ब्‍लॉक को चुनें जिसे आप सेवाएं प्रदान करते हैं: लागू नहीं/ यदि लागू होता है तो बताएं

Does the district you are serving have tribal populations: Yes/No

क्‍या जिस जिले को आप सेवाएं प्रदान करते हैं उसमें जनजातीय आबादी है: हां/नहीं

The number of years in service:

कितने सालों से सर्विस में हैं:

Age:

आयु:

Phone number (only to get in touch with you for future clarification if any :

फोन नंबर (केवल भविष्‍य के स्‍पष्टिकरण के लिए, यदि कोई हो, आपसे संपर्क करने के लिए)

1. Are high-risk pregnancies a cause for concern in the district?

क्‍या जिले में उच्‍च जोखिम वाली गर्भावस्‍था चिंता का विषय हैं?

1. Yes, to some extent

हां, कुछ हद तक

1. Yes, to a large extent

हां, काफी हद तक

1. No

नहीं

1. What percentage of pregnant women in the district register for antenatal care in the first trimester?

जिले में कितने प्रतिशत गर्भवती महिलाएं प्रथम तिमाही में प्रसवपूर्व देखभाल के लिए पंजीकरण कराती हैं?

1. Less than 20%

20% से कम

1. Between 21-40%

21-40% के बीच में

1. Between 41-60%

41-60% के बीच में

1. Between 61-80%

61-80% के बीच में

1. Between 81 to 100%

81 -100% के बीच में

1. Are there any areas, with low early-ANC registration?

क्या कोई ऐसे मंडल हैं, जहां प्रारंभिक एएनसी पंजीकरण कम हैं?

1. Yes हां

2. No नहीं

3a. IF yes, please mention which areas and reason for low ANC registration?

यदि हां, तो कृपया बताएं कि कौन से मंडल?

_______________________________________________________________________

3b. What is the 3 rd and 4 th ANC uptake in the district?

जिले में तीसरा और चौथा एएनसी अपटेक क्या है?

3c, Are there any associated reasons (Low early ANC, 3 rd and 4 th ANC visits) for the same?

क्या इसके लिए कोई जुड़े हुए कारण (कम प्रारंभिक एएनसी, तीसरी और चौथी एएनसी विजिट्स) हैं?

___________________________________________________________________________ ___________________________________________________________________________

1. Are there areas or areas with negative or challenging pregnancy outcomes or challenges?

क्या ऐसे क्षेत्र या मंडल हैं जिनमें गर्भावस्था के नकारात्मक या चुनौतीपूर्ण परिणाम या चुनौतियां हैं?

1. Yes हां

2. No नहीं

4a. If yes, which are these areas? And how many such blocks are there in the district, of the total number of blocks?

यदि हां, तो ये मंडल कौन से हैं? और जिले में, कुल ब्‍लॉक्‍स में ऐसे ब्‍लॉक्‍स की संख्‍या कितनी है

4b. Are there specific social, economic, cultural, environmental or other factors that affect the pregnancy outcomes? *[probe: occupation and pregnancy, food practices – by caste, class, religion, causes for anaemia, violence, poverty, other disease-specific to regional conditions, perception of the need for medical care during pregnancy, perception on taking iron, folic acid supplements, seeking care at the hospital for illness etc]*

क्या कोई ऐसे विशेष सामाजिक, आर्थिक, सांस्कृतिक, पर्यावरणीय या अन्य कारक हैं जो गर्भावस्था के परिणामों को प्रभावित करते हैं? [प्रोब करें: व्यवसाय और गर्भावस्था, भोजन के तरीके - जाति, वर्ग, धर्म के अनुसार, एनीमिया के कारण, हिंसा, गरीबी, क्षेत्रीय परिस्थितियों के अनुसार अन्य रोग विशिष्‍ट, गर्भावस्था के दौरान चिकित्सा देखभाल की आवश्यकता की धारणा, आयरन, फोलिक एसिड सप्‍लीमेंट्स की खुराक लेने की धारणा, बीमारी के लिए अस्पताल में देखभाल लेना आदि]

1. Could you prioritize the high-risk conditions based on the prevalence (rank the order)

क्या आप व्यापकता के आधार पर उच्च जोखिम वाली समस्‍याओं को प्राथमिकता के क्रम में लगा सकते हैं (क्रम को रैंक करें)

| Anaemia  एनीमिया |  | Malaria  मलेरिया |  | Hypertension  हाइपरटेंशन |  |
| --- | --- | --- | --- | --- | --- |
| Thalassemia/sickle cell disease  थैलेसीमिया/सिकल सेल रोग |  | Fever  बुखार |  | Breech presentations  ब्रीच रिप्रेजेंटेशंस |  |
| Diabetes  डायबिटीज |  | Mental health  मानसिक रोग |  | Pre-term labour  समय से पहले प्रसव पीड़ा |  |
| HIV  एचआईवी |  | Heart illness  हृदय रोग |  | RTI/STI  आरटीआई/एसटीआई |  |
| Foetal growth restriction  भ्रूण विकास की सीमा |  | Thyroid  थायराइड |  | Bleeding in pregnancy  गर्भावस्‍था में रक्‍तस्राव |  |
| Syphilis  सिफलिस |  | Jaundice  पीलिया |  | Tuberculosis  टुबर्क्यलोसिस |  |
| Pre-term rupture of membranes  झिल्ली का समय से पहले टूटना |  |  |  |  |  |

6. Roughly, in a month what is the average number of pregnancies registered with the public health system (2023-24)? _______ .

मोटे तौर पर, एक महीने में पब्लिक हेलथ सिस्‍टम (2023-24) में पंजीकृत गर्भधारण की औसत संख्या कितनी है?

7. What percentage of total pregnancies in the district is handled by the public sector health institutions?

जिले में कुल गर्भधारण का कितना प्रतिशत सार्वजनिक क्षेत्र के स्वास्थ्य संस्थानों बनाम निजी संस्थानों द्वारा संभाला जाता है?

1. Up to 20%

20% तक

2. 20-40 %

3. 40-50%

4. 50-60%

5. 60 -80%

6. 80-100%

8. What percentage of births happen at home?_________

कितने प्रतिशत प्रसव घर पर होते हैं?

9. Of the total pregnancies registered within the public health system in your district, what percentage of pregnancies during the antenatal period is managed at: (irrespective of registration status)

आपके जिले में पब्लिक हेल्‍थ सिस्‍टम के अंतर्गत पंजीकृत कुल गर्भधारण में से, प्रसवपूर्व अवधि के दौरान कितने प्रतिशत गर्भधारण का प्रबंधन इनके द्वारा किया जाता है: (पंजीकरण स्थिति पर ध्यान दिए बिना)

a. PHC level ____________%

पीएचसी स्‍तर

b. Secondary institutions (CHC/AH/District hospital)____________%

माध्‍यमिक संस्‍थान (सीएचसी/एएच/जिला अस्‍पताल)

c. Tertiary institutions (teaching hospitals)____________%

टर्शरी संस्‍थान (टीचिंग हॉस्पिटल्‍स

1. Of the total pregnancies, what percentage of these pregnancies could be categorized as high-risk pregnancies requiring attention?

कुल गर्भधारणों में से कितने प्रतिशत गर्भधारणों को उच्च जोखिम वाले गर्भधारणों की कैटेगरी में रखा जा सकता है जिन पर ध्यान देने की आवश्यकता होती है?

1. Upto 20%

20% तक

2. 20-40 %

3. 40-50%

4. 50-60%

5. Above 60%

60% से अधिक

1. What percentage of pregnancies from PHCs/CHCs/AHs are currently referred to tertiary care centres due to high-risk pregnancy category? ____________%

उच्च जोखिम वाली गर्भावस्था कैटेगरी के कारण वर्तमान में पीएचसी/सीएचसी/एएच से कितने प्रतिशत गर्भधारण को टर्शर केयर सेंटर में रेफर किया जाता है?

12. List the most commonly referred high-risk conditions by trimester?
 ट्राइमेस्टर के आधार पर सबसे आम तौर पर रेफर किये जाने वाले उच्च जोखिम वाली समस्‍याओं की सूची बनाएं?

First trimester –

पहला ट्राइमेस्टर

Second trimester –

दूसरा ट्राइमेस्टर

Third trimester –

तीसरा ट्राइमेस्टर

13. What is the percentage of stillbirths, in the district to the total live births?_____

जिले में कुल जीवित जन्मों में से मृत बच्चे का जन्म का प्रतिशत कितना है?

14. Are there any reasons or causes one could attribute to these stillbirths within the district?

क्या जिले में इन मृत बच्‍चों केजन्मों के लिए कोई कारण या वजहें बताई जा सकती है?

15. What are the current measures in place to reduce the occurrence of stillbirths?

मृत बच्‍चे के जन्म की घटनाओं को कम करने के लिए इस समय क्या उपाय किए जा रहे हैं?

16. Do you think all high-risk pregnancies are currently identified in the early antenatal period?

क्या आपको लगता है कि सभी उच्च जोखिम वाली गर्भधारण की पहचान वर्तमान में प्रारंभिक प्रसवपूर्व अवधि में ही कर ली जाती है?

1. Yes हां

2. No नहीं

17. Are there reasons for not being able to identify, and monitor high-risk pregnancies in the early antenatal period and follow? [Multiple options tick all applicable answers; yes-1, No-2

क्या ऐसे कोई कारण हैं जिनकी वहज से उच्च जोखिम वाली गर्भावस्थाओं की पहचान नहीं हो पाती, और उनकी प्रसवपूर्व अवधि में निगरानी नहीं की जा पाती है तथा उनको फॉलो नहीं किया जा पाता है? [ कई विकल्‍प लागू होने वाले सभी जवाबों को टिक करें; हाँ-1, नहीं-2

1. Women register late for pregnancy

महिलाएं गर्भावस्‍था के लिए देरी से पजीकरण कराती हैं

2. ANMs/PHNs not trained for high-risk identification

एएनएम/पीएचएन उच्च जोखिम की पहचान करने के लिए ट्रेन्‍ड नहीं हैं

1. MOs not trained for high-risk identification

एमओ उच्च जोखिम की पहचान करने के लिए ट्रेन्‍ड नहीं हैं

1. The quality of ANC care provided by the Mo and the time spent on each ANC

एमओ द्वारा प्रदान की जाने वाली एएनसी देखभाल की गुणवत्ता और प्रत्येक एएनसी पर लगाया जाने वाला समय

1. There are currently no proper management guidelines for high-risk pregnancy

मौजूदा समय में उच्च जोखिम वाली गर्भावस्था के लिए कोई उचित प्रबंधन दिशानिर्देश नहीं हैं

1. There is a lack of data integration for follow-up and referral at a different institutional level

विभिन्न संस्थागत स्तर पर फॉलो-अप और रेफरल के लिए डेटा इंटीगेशन की कमी है

1. PHCs are not equipped with adequate infrastructure and equipment

पीचएस के पास पर्याप्त इन्‍फ्रास्‍ट्रक्‍चर और उपकरण नहीं हैं

18. What are the hurdles in handling high-risk pregnancies at the PHC level?

पीएचसी स्तर पर उच्च जोखिम वाली गर्भावस्थाओं को संभालने में क्या बाधाएं हैं?

1. Lack of adequate infrastructure (Building and medical equipments) at PHC level

पीएचसी स्तर पर पर्याप्त इन्‍फ्रास्‍ट्रक्‍चर (बिल्डिंग और चिकित्सा उपकरण) की कमी

1. Lack of knowledge among medical officers
2. मेडिकल ऑफिसर में ज्ञान की कमी
3. Lack of human resources

मानव संसाधन की कमी

1. Fear of handling at PHC level

पीएचसी स्‍तर पर संभालने का डर

1. Lack of proper guidelines

उचित दिशा-निर्देशों की कमी

19. Are the MOs sufficiently equipped (knowledge and guidance) to handle high-risk pregnancies at the PHC level?

क्या मेडिकल ऑफिसर पीएचसी स्तर पर उच्च जोखिम वाली गर्भावस्थाओं को संभालने के लिए पर्याप्त रूप से सुसज्जित (ज्ञान और मार्गदर्शन) हैं?

1. Yes, all of them

हां, उनमें से सभी

1. Yes, some of them

उनमें से कुछ

3. No नहीं

20. Do the medical officers have sufficient knowledge and guidance to assesses, manage, and refer for high-risk pregnancies appropriately?

क्या मेडिकल ऑफिसर्स के पास उच्च जोखिम वाली गर्भावस्थाओं का उचित मूल्यांकन, प्रबंधन और रेफर करने के लिए पर्याप्त ज्ञान और मार्गदर्शन है?

1. Yes, completely

हां, पूरी तरह से

1. To some extent

हां, कुछ हद तक

3. No नहीं

21. What do you think is the role of ANMs in handling high-risk pregnancies?

आपको क्‍या लगता है कि उच्च जोखिम वाली गर्भावस्थाओं को संभालने में एएनएम की क्‍या भूमिका है?

1. Identify high-risk pregnancies by taking proper history

उचित इतिहास को जाकर उच्च जोखिम वाली गर्भावस्थाओं की पहचान करना

1. Guide the women on appropriate pregnancy care and provide counselling to women and their family members

महिलाओं को उचित गर्भावस्था देखभाल के बारे में मार्गदर्शन देना और महिलाओं और उनके परिवार के सदस्यों को परामर्श देना

1. Refer to the appropriate health facility

उचित स्‍वास्‍थ्‍य सुविधा पर रेफर करना

4. 1,2,3

5. None of the above

इनमें से कोई नहीं

22. Do the ANMs have sufficient knowledge and guidance to identify, manage at community level, and refer high-risk pregnancies appropriately?

क्या एएनएम के पास उच्च जोखिम वाली गर्भावस्थाओं की पहचान करने, सामुदायिक स्तर पर प्रबंधन करने तथा उचित तरीके से रेफर करने के लिए पर्याप्त ज्ञान और मार्गदर्शन है?

1. Yes, completely

हां, पूरी तरह से

1. To some extent

हां, कुछ हद तक

3. No नहीं

23. Is access to care to public health hospitals affected in specific blocks, populations, communities, or geographic areas within the district?

क्या जिले के भीतर विशेष ब्लॉक, आबादी, समुदायों या भौगोलिक क्षेत्रों में सार्वजनिक स्वास्थ्य हॉस्पिटल्‍स तक देखभाल की पहुंच प्रभावित है?

1. Yes, to some extent

हां, कुछ हद तक

1. No, to a great extent

नहीं, काफी हद तक

3. No नहीं

23a. If affected for certain groups or populations please explain, and how it affects the pregnancy outcomes?

यदि कुछ समूहों या आबादी के लिए प्रभावित है तो कृपया बताएं और और समझाएं कि यह गर्भावस्था के परिणामों को कैसे प्रभावित करता है?

24. How do use the MCH data for planning, monitoring, review and actions? According to you which indicators are very important to understanding high-risk pregnancies?

योजना बनाने, निगरानी करने, ​​समीक्षा और कार्रवाई करने के लिए आप एमसीएच डेटा का इस्‍तेमाल कैसे करते हैं? आपके अनुसार उच्च जोखिम वाली गर्भावस्था को समझने के लिए कौन से संकेतक बहुत महत्वपूर्ण हैं?

25. Are there specific initiatives at the district level to improve the positive pregnancy outcomes?

क्या गर्भावस्था के अच्‍छे परिणामों को बेहतर बनाने के लिए जिला स्तर पर कोई विशेष पहल की गई है?

1. Yes हां

2. No नहीं

25a. If yes, please detail those initiatives.

यदि हां, तो कृपया उन पहलों पर विस्‍तार से बताएं

26. Do you think proper history taking by ANMs, identification, management and data integration at all levels of the health system, improve the quality of care and management at the district level?

क्या आपको लगता है कि हेल्‍थ सिस्‍टम के सभी स्तरों पर एएनएम द्वारा उचित इतिहास लेने, पहचान करने, प्रबंधन करने और डेटा इंटीग्रेशन से जिला स्तर पर देखभाल और प्रबंधन की गुणवत्ता में सुधार होगा?

1. Yes, to a great extent

हां, काफी हद तक

1. Yes, to some extent,

हां, कुछ हद तक

1. Not sure

पक्‍का नहीं

4. No नहीं

27. Do you think there is a need for a proper High-risk pregnancy management guideline for ANMs, MOs and specialists?

क्या आपको लगता है कि एएनएम, एमओ और स्‍पेशलिस्‍ट के लिए उच्च जोखिम वाली गर्भावस्था के प्रबंधन पर उचित दिशानिर्देश की आवश्यकता है?

1. Yes हां

2. No नहीं

27a. If yes, do you think there is a need to conduct trainings to the ANMs, doctors and specialists?

यदि हां, तो क्‍या आपको लगता है कि एएनएम, डॉक्‍टर्स और स्‍पेशलिस्‍ट के लिए ट्रेनिंग आयोजित करने की आवश्‍यकता है

1. Yes हां

2. No नहीं

28. Do you think by managing high-risk pregnancies effectively, we could improve the current maternal and child mortality in the state of Uttar Pradesh?

क्या आपको लगता है कि उच्च जोखिम वाली गर्भावस्थाओं का प्रभावी प्रबंधन करके, हम उत्तर प्रदेश राज्य में मौजूदा मातृ और शिशु मृत्यु दर में सुधार कर सकते हैं?

1. Yes, to some extent

हां, कुछ हद तक

1. Yes, to a great extent

हां, काफी हद तक

1. Doubtful

संदेह है

4. No नहीं

29. Do you have any suggestions to improve the high-risk pregnancy management or stillbirth in the state of Uttar Pradesh?

क्या आपके पास उत्तर प्रदेश राज्य में उच्च जोखिम वाली गर्भावस्था के प्रबंधन या मृत शिशु जन्म में सुधार करने के लिए कोई सुझाव है?
