# Supplementary material for: Impact of a Multicomponent Intervention to Build Capacity of Public Health Workers to Make Algorithmic Diagnosis and Management of High-Risk Pregnancies in Uttar Pradesh, India: Protocol for a Matched-Control, Before-After, Quasi-Experimental Study With a Mixed Methods Design
Source: JMIR Res Protoc. 2025 Dec 9;14:e74993. doi: 10.2196/74993 (PMC12690279; doi:10.2196/74993)
Supplement: Multimedia Appendix 3 [file resprot-v14-e74993-s003.docx]

**Annexure 3:**

**In-Depth Interview with Specialist**

**Informed Consent Form– 1**

Dear Sir/Madam

**Introduction:** Greetings. I am _______________ (name), from ARMMAN. ARMMAN is an India based not-for-profit organization that leverages technology to create scalable solutions empowering mothers and enabling healthy children. ARMMAN is committed to improving the well-being of pregnant women, mothers and children in the first 5 (five) years of their life.

I will provide detailed information about the study in just a few minutes. But before I do that, I want to give you a short summary to help you decide if you want to take part in this study. You need to know:

1. This research is being done to understand systems and its stakeholder’s readiness to diagnose & manage high-risk pregnancies in the district.
2. Whether you take part is your decision.
3. You do not have to take part;
4. You can change your mind at any time;
5. Your decision will not be held against you by ARMMAN or anyone else;
6. If you take part, you will be asked to participate in a survey which will take around 45 to 60 minutes.
7. You will not benefit directly from taking part, but we hope to be able to help others in the future.
8. The primary risk to you if you take part is that others may find out the information you’ve shared, but we will try not to let this happen.

**Purpose:** ARMMAN in partnership with the Health and Family Welfare Department, the Government of Uttar Pradesh is implementing the High-Risk Pregnancy Tracking & Management (IHRPTM) program in two intervention and two control districts of UP. We are initiating the online capacity building program designed based on the High-risk Pregnancy Management guidelines developed for the ANMs, MOs, SNs and specialists for six high-risk pregnancy (HRP) conditions based on the prevailing conditions and suggestions from experts in Uttar Pradesh. We are preparing to implement the technological platform to strengthen the health system pertinent to maternal and child health care by developing an app for additional support to ANMs and MOs apart from training and an app developed to track high-risk pregnancies and integrated with the RCH portal. This will enable the availability of women’s pregnancy-related information accessible at levels of health care providers providing services to plan, prepare and provide quality services to antenatal women.

**Risks & Benefits:** A possible risk to taking part in this study is that people outside the research team may find out your answers to the questions. We try and make it so this will not happen, and you will not be identified by your name or designation. Your shared insights will help in the design, conceptualisation of the indicators for monitoring and evaluation, and implementation of the IHRPTM program in UP.

**Confidentiality:** We will do everything we can to keep the information you share is secret. All the information you share will not be identified with your name or designation. This interview shall take place in private and it shall take 45-60 minutes.

**Recording:** I would also like to record this interview so that any information provided by you is not missed out.

**Oral consent and recording:** I have read the consent form. I understand that I am being asked to take part in the IHRPTM research study. I understand I can keep a copy of this form if I want so that I can review later, contact someone about the study, or keep for my records.

I consent to take part in this study and provide permission to record my interview. I understand that if I want to stop taking part I may do so at any time.

Do you consent to participate in this study? YES NO

Do you consent to record the interview? YES NO

Please let me know if you would like to keep a copy of this form so that you can review the information at a later date, contact someone about the study, or keep it for your records.

Name of the Interviewer: ____________________

Date: __________________

**Contact** If you have any questions or concerns regarding this interview, please connect with: ARMMAN contact: Dr. Hanimi Reddy Modugu , Email: hanimi@armman.org, Mobile: +91 99118 22445

**Specialists**

*[Record institutional affiliation, Block, Division, education qualification, and years of experience].*

1. What is the current per day load of antenatal check-ups from your division? How does the referral from PHCs regulated or structured – 3rd ANC visits? Can you say which month the 3rd and 4th ANC visits are scheduled?

2. What is the nature of cases currently referred for delivery due to high-risk pregnancy from the PHC? Could you explain the percentage of referrals? From which stage of the antenatal period women are referred – by conditions? Are there any current protocols?

3. Are there challenges as a tertiary level institution in managing the ANC cases? If so, how? (Referrals, history of the patient, follow-up, infrastructure, manpower)

4. Are there challenges in the maternal or child outcomes due to how the pregnant women were managed or tracked during the antenatal period?

5. How are those women who visit the tertiary centre directly without approaching the assigned ANM or supervisor or PHC medical officer tracked?

6. How do you communicate the update on the ANC follow-up care to the ANMs or MOs of the PHC woman is affiliated with?

7. Currently, as specialists do you have any targets or roles apart from providing clinical services to the patients during ANC and labour?

8. Are there instances or reasons women choose the private sector after initially coming to the public sector during ANC?

9. What are the current mechanisms to coordinate higher-level referrals from the district? For what kind of cases do such referrals take place and the extent of such referrals?

10. Are there any suggestions to improve the high-risk pregnancy management and tracking?
