# Supplementary material for: Impact of a Multicomponent Intervention to Build Capacity of Public Health Workers to Make Algorithmic Diagnosis and Management of High-Risk Pregnancies in Uttar Pradesh, India: Protocol for a Matched-Control, Before-After, Quasi-Experimental Study With a Mixed Methods Design
Source: JMIR Res Protoc. 2025 Dec 9;14:e74993. doi: 10.2196/74993 (PMC12690279; doi:10.2196/74993)
Supplement: Multimedia Appendix 4 [file resprot-v14-e74993-s004.docx]

**Annexure 4**

**Medical Officer (MO)**

**मेडिकल ऑफिसर (एमओ)**

**Informed Consent Form**

सूचित सहमति फॉर्म

[Baseline Survey to Understand the Medical Officers Knowledge, Attitude and Practices on High-risk Pregnancy Management in Telangana]

[तेलंगाना में हाई-रिस्‍क वाली गर्भावस्‍था के प्रबंधन में मेडिकल ऑफिसर की जानकारी, दृष्टिकोण और व्‍यवहारों को समझने के लिए बेसलाइन सर्वे]

Dear Doctor

डियर डॉक्‍टर

Greetings to you. ARMMAN in partnership with Government of Uttar Pradesh is going to conduct training for all MOs to improve their knowledge and skills to handle high-risk pregnancies. Very soon all MOs will have training sessions to expose you to the high-risk pregnancy diagnosis and management, using the colour coded protocols developed for six priority high risk conditions identified by the state of Uttar Pradesh. Before ARMMAN initiates the training, it is important to understand the current practices followed by MOs to diagnose, manage, refer and follow-up done to women with HRPs. Purpose of baseline survey is to understand these aspects.

आपका स्‍वागत है। उत्‍तर प्रदेश में सरकार के साथ पार्टनरशिप में अरमान सभी एमओ की ट्रेनिंग आयोजित करने जा रहा है ताकि उच्‍च-जोखिम वाली गर्भावस्‍थाओं को संभालने के लिए उनके ज्ञान और कौशल में सुधार किया जा सके। बहुत जल्द ही सभी एमओ लिए ट्रेनिंग सत्र आयोजित किये जाएंगे, जिसमें आपको उच्च जोखिम वाली गर्भावस्था का पता लगाने और उनके प्रबंधन के बारे में बताया जाएगा, जिसमें उत्तर प्रदेश राज्य द्वारा पहचानी गई छह प्राथमिकता वाली उच्च जोखिम वाली समस्‍याओं के लिए तैयार किये गए कलर कोडेड प्रोटोकॉल का किया जाएगा। इससे पहले की अरमान ट्रेनिंग शुरू करे, एचआरपी से पीड़ित महिलाओं में डायग्‍नोस, प्रबंधन, रेफर करने और फॉलो-अप के लिए एमओ द्वारा अपनाई जाने वाले मौजूदा तरीकों को समझना महत्वपूर्ण है। बेसलाइन सर्वेक्षण का उद्देश्य इन पहलुओं को समझना है।

Please read the questions and answer the questions that you feel is right. It is okay if you are not aware of some questions. You can say you do not know or skip the question. Purpose of baseline is not to evaluate you. It is only to understand your current understanding and practices pertinent to HRP management.

कृपया प्रश्‍नों को पढें और प्रश्‍नों का वह जवाब दें जो आपको सही लगता है। अगर आपको कुछ प्रश्‍नों के बारे में नहीं पता है तो कोई बात नहीं। आप कह सकते हैं कि आपको नहीं पता या प्रश्‍न को छोड़ सकते हैं। बेसलाइन का उद्देश्‍य आपका मूल्‍यांकन करन नहीं है। यह सिर्फ एचआरपी मैनेजमेंट के संबंध में आपकी मौजूदा समझ और अभ्‍यास को समझने के लिए है।

The survey responses will not be identified with your name or mobile phone number or anything specific with your identity. The responses of all the MOs will be looked at together to understand the objectives of this survey. We intend to use this information to present to the state authorities, as part of situational assessment of HRP management in the state. The findings of this survey will be disseminated with Government of Uttar Pradesh and to other appropriate audience and/or platforms. Learning from this survey will also feed into designing of training plans, implementation of HRP management program in the state. This survey shall take 30-40 minutes.

सर्वे के जवाबों को आपके नाम या मोबाइल फ़ोन नंबर या आपकी पहचान से जुड़ी किसी भी विशेष चीज़ से पहचाना नहीं जाएगा। इस सर्वे के उद्देश्यों को समझने के लिए सभी एमओ के जवाबों को एक साथ देखा जाएगा। हम इस जानकारी का इस्‍तेमाल राज्य के अधिकारियों को राज्य में एचआरपी के स्थितिजन्य मूल्यांकन के भाग के रूप में प्रस्तुत करने के लिए करना चाहते हैं। इस सर्वे के निष्कर्षों को उत्तर प्रदेश सरकार और अन्य उपयुक्त दर्शकों और/या प्लेटफार्मों के साथ साझा किया जाएगा। इस सर्वे से प्राप्त जानकारी का इस्‍तेमाल ट्रेनिंग योजनाओं को तैयार करने, राज्य में एचआरपी मैनेजमेंट प्रोग्राम के कार्यान्वयन में भी किया जाएगा। इस सर्वे में 30-40 मिनट का समय लगेगा।

. Prior to participating in this survey, please answer the following.

इस सर्वे में भाग लेने से पहले, कृपया निम्‍नलिखित का जवाब दें।

1. Do you agree to take part in this survey?

क्‍या आप इस सर्वे में भाग लेने के लिए सहमत हैं?

1. Yes हां
2. No नहीं
3. Do you understand the purpose of this survey?

क्‍या आप इस सर्वे के उद्देश्‍य को समझते हैं?

1. Yes हां
2. No नहीं

Name of the Interviewer: ____________________

इंटरव्‍यूअर का नाम:

Date: __________________

तारीख:

**Contact** If you have any questions or concerns regarding this interview, please connect with: ARMMAN contact: Dr. Hanimi Reddy Modugu , Email: hanimi@armman.org, Mobile: +91 99118 22445

**संपर्क** यदि आपके पास इस इंटरव्‍यू के संबंध में कोई प्रश्‍न या चिंताएं हैं, तो कृपया निम्‍न से संपर्क करें: अरमान का कॉन्‍टेक्‍ट: डॉ. हनिमी रेड्डी मोडुगु, ईमेल: hanimi@armman.org, मोबाइल: +91 99118 22445

Signature of MO: _______________________ Date:___________

एमओ के हस्‍ताक्षर तारीख

**Background information**

**पृष्‍ठभूमि की जानकारी**

Gender: 1. Male 2. Female 3. Other

लिंग: 1. पुरूष 2. महिला 3. अन्‍य

Age (in years):

आयु (सालों में)

Educational qualification:

शैक्षिक योग्‍यता

District currently serving:

जिस जिले में अभी सेवा प्रदान कर रहे हैं:

Name of the PHC currently servicing:

पीएचसी का नाम जिसे इसे समय सेवाएं प्रदान कर रहे हैं: 1.

Number of years in government service (Example for 8 months: 0.8):

सरकारी सेवाओं में सालों की संख्‍या (उदाहरण के लिए 8 महीने: 0.8):)

Number of years of service completed in the current PHC:

वर्तमान पीएचसी में पूरे किये गए सेवा के सालों की संख्‍या:

**HRP situation in your area**

आपके क्षेत्र में एचआरपी की स्थिति

Average number of ANCs at this PHC, per month:

इस पीएचसी में हर महीने एनएनसी की औसत संख्‍या:

Average number of pregnancies that are high-risk pregnancies out of all pregnancies in your PHC, per month:_

आपके पीएचसी में प्रति माह सभी गर्भावस्‍थाओं में से उच्च जोखिम वाली गर्भावस्‍थाओं की औसत संख्या:_

Of the total high-risk pregnancies, how many are referred to designated facilities or specialists during ANC/delivery, per month: _________

**कुल उच्च जोखिम वाली गर्भावस्थाओं में से, प्रति माह एएनसी/प्रसव के दौरान कितनी को नामित सुविधाओं या स्‍पेशलिस्‍ट के पास भेजा जाता है:**

**Scope of work**

**कार्य क्षेत्र**

Do you think that PHC/MO can play an important role for **all high risk pregnancy conditions** during the antenatal period?

क्‍या आपको लगता है कि आप प्रसवपूर्व अवधि के दौरान सभी उच्‍च जोखिम वाली गर्भावस्‍था की स्थितियों के लिए एक महत्‍वपूर्ण भूमिका निभा सकते हैं?

1. To a great extent

काफी हद तक

1. To some extent

कुछ हद तक

1. Not at all

बिल्‍कुल भी नहीं

1. Not sure

पक्‍का नहीं

Do you think it is PHC’s/MO responsibility to treat ALL high-risk conditions that could be managed at the PHC level as a medical officer?

क्या आपको लगता है कि एक मेडिकल ऑफिसर के रूप में उन सभी उच्च जोखिम वाली समस्‍याओं का इलाज करना आपकी जिम्मेदारी है, जिनका उपचार पीएचसी स्‍तर पर किया जा सकता है?

1. To a great extent

काफी हद तक

1. To some extent

कुछ हद तक

1. Not at all

बिल्‍कुल भी नहीं

1. Not sure

पक्‍का नहीं

Do you think it’s PHC/MO responsibility to see how well the pregnancies are **managed**, **monitor** their health status, **follow-up** and **referra**l status at your PHC level?

**क्या आपको लगता है कि यह देखना आपकी जिम्मेदारी है कि गर्भावस्था की देखरेख कितनी अच्छी तरह से की जा रही है, उनके स्वास्थ्य की स्थिति की निगरानी करना, अपने पीएचसी स्तर पर फॉलोअप और रेफरल की स्थिति की निगरानी करना?**

1. To a great extent

काफी हद तक

1. To some extent

कुछ हद तक

1. Not at all

बिल्‍कुल भी नहीं

1. Not sure

पक्‍का नहीं

*Choose the THREE top mLScost High risk conditions, higher in terms of number of cases (in order of higher to lower) that complicates pregnancy that you commonly see in your area:*

*अपने क्षेत्र में आमतौर पर देखी जाने वाली तीन सबसे उच्च जोखिम वाली समस्‍याओं को चुनें, जो केसेज की संख्या के मामले में अधिक हैं (उच्च से निम्न के क्रम में) जो गर्भावस्था को जटिल बनाती हैं:*

Rank 1.

रैंक 1

Rank 2.

रैंक 2

Rank 3.

रैंक 3

**According to you assign whether the below mentioned conditions is perceived by you as a high risk pregnancy condition or not? If you are not sure and can’t decide, please select not sure.**

**अपने अनुसार असाइन करें कि क्‍या नीचे दी गई समस्‍याएं आपके द्वारा उच्च जोखिम वाली गर्भावस्था संबंधी समस्‍याएं मानी जाती हैं या नहीं? यदि आपको पक्‍का नहीं पता है और निर्णय नहीं ले पा रहे हैं, तो कृपया पक्‍का नहीं चुनें।**

| **S.No** | **Condition** | 1. **Yes, HRहां, एचआर** 2. **Not HRनहीं, एचआर** 3. **Not sureपक्‍का नहीं** |
| --- | --- | --- |
| **1** | 17 yr. old primigravida, 24 weeks POG (Period of Gestation)  **17 वर्षीय प्राइमिग्रेविडा, 24 सप्ताह पीओजी (गर्भावस्था की अवधि)** |  |
| **2** | G7P6L6, 37 weeks POG with labour pains, Hb 11.5 gm/dl  G7P6L6**, प्रसव पीड़ा के साथ 37 सप्ताह का पीओजी, Hb 11.5 gm/dl** |  |
| **3** | Primigravida 32 wks. POG with BP 160/110 mmHg  **प्राइमिग्रेविडा 32 सप्ताह POG के साथ बीपी 160/110 mmHg** |  |
| **4** | G2P1L1, 10 wks. POG with TSH >6 uIU/ml  **G2P1L1, 10 सप्ताह। TSH >6 uIU/ml के साथ पीओजी** |  |
| **5** | G3P2L2 20 wks. POG with Hb 6.5 mg/dl  **G2P1L1, 10 सप्ताह।** होमोग्‍लोबिन 6.5 mg/dl **के साथ पीओजी** |  |
| **6** | G2P1L1, 38 wks. POG with H/O previous delivery by LSCS  G2P1L1, 38 **सप्ताह।** एलएससीएस द्वारा एच/ओ पिछली डिलीवरी के साथ पीओजी |  |
| **7** | Primigravida 28 weeks with painless bleeding P/V  **दर्द रहित रक्तस्राव पी/वी के साथ प्राइमिग्रेविडा 28 सप्ताह** |  |
| **8** | G7P6L6, 38 weeks POG with breech presentation  **G7P6L6, ब्रीच रिप्रेजेंटेशन के साथ 38 सप्ताह पीओजी** |  |
| **9** | G2P1L1, 24 wks. POG with watery discharge P/V  G2P1L1, 24 सप्‍ताह। पानी जैसे डिस्चार्ज पी/वी के साथ पीओजी |  |
| **10** | G3P2L2 with 24 weeks POG with palpitations and breathlessness on mild activity  G3P2L2 **24 सप्ताह के पीओजी के साथ हल्की सी गतिविधि पर घबराहट और सांस फूलना** |  |
| **11** | G7P6L6, 37 weeks POG with Hb 7 gm/dl  G7P6L6, 37 सप्‍ताह के पीओजी के साथ होमोग्‍लोबिन 7 gm/dl |  |
| **12** | Primigravida 34 weeks with twin pregnancy with Hb 12gm/dl  **एचबी 12gm/dl के साथ जुड़वां गर्भावस्था के साथ प्राइमिग्रेविडा 34 सप्ताह** |  |
| **13** | Primigravida 34 weeks with seizures and headache  **दौरे और सिरदर्द के साथ प्राइमिग्रेविडा 34 सप्ताह** |  |
| **14** | 38 yr. Primigravida 32 weeks POG with Hb 12 mg/dl  **38 वर्ष, प्राइमिग्रेविडा, 32 सप्ताह, Hb 12 mg/dl के साथ के साथ पीओजी** |  |
| **15** | G2P1L1, 34 wks. POG with previous LSCS with pain abdomen with BPV (bleeding per vagina)  **G2P1L1, 34 सप्ताह। पिछले एलएससीएस के साथ पीओजी साथ ही बीपीवी (योनि से रक्तस्राव) के साथ पेट में दर्द** |  |

**Kindly suggest where do currently treat or refer for the following conditions?**कृपया सुझाव दें कि मौजूदा समय में आप निम्नलिखित समस्‍याओं के लिए कहां उपचार किया या रेफर किया जाता है?

| **S.No** | **Condition** | 1. **Treat at PHC**   **पीएचसी पर उपचार**   1. **Refer to a higher facility/ob-gyn**   **उच्‍च सुविधा/** **प्रसूति एवं स्त्रीरोग विशेषज्ञ को रेफर करते हैं**   1. **Stabilise and refer to a higher facility**   **स्थिर करते हैं और उच्च सुविधा को रेफर करते हैं** |
| --- | --- | --- |
| **1** | Primigravida 20 weeks pregnancy with Hb < 10 gm% |  |
| **2** | Gravida 2 with one living issue, 34 weeks POG with Hb < 7 |  |
| **3** | Gravida 2 with one live issue term pregnancy with h/o previous delivery by LSCS |  |
| **4** | Primigravida 32 weeks POG with BP > 150/90 |  |
| **5** | Gravida 3, 24 weeks with OGTT 260 mg/dl |  |
| **6** | Primigravida 36weeks POG with BP > 140/90, with headache |  |
| **7** | Gravida 3, 10 weeks POG with pain lower abdomen and spotting |  |
| **8** | Gravida 3, 32 weeks POG with painless bleeding |  |
| **9** | Gravida 4, with 3 living issues with 34 weeks POG with Prev 2 LSCS with pain and bleeding |  |
| **10** | Gravida 2 with one live issue term pregnancy with h/o epilepsy |  |
| **11** | Primigravida 20 weeks POG with breathlessness and palpitations |  |
| **12** | Gravida 3, 24 weeks with jaundice |  |

**Kindly choose the medical tests you currently provide at your PHC to pregnant women?**

कृपया उन मेडिकल टेस्‍ट्स को चुनें जो आप वर्तमान में अपने पीएचसी पर गर्भवती महिलाओं को प्रदान करते हैं?

BG बीजी Yes हां No नहीं

Haemoglobin

होमोग्‍लोबिन Yes No

Urine routine & microscopyयूरीन रूटी और माइक्रोस्‍कोपिक Yes No

Hepatitis हेपेटाइटिस Yes No

HIV एचआईवी Yes No

RPR/VDRL

आरपीआर/वीडीआरएल Yes No

Blood grouping of husband पति के साथ ब्‍लड ग्रुपिंग Yes No

Glucose OGTT

ग्‍लूकोज ओजीटीटी Yes No

Urine culture

यूरीन कल्‍चर Yes No

Urine-protein/ Sugar

यूरीन-प्रोटी/शुगर Yes No

Urobilinogen-dipstic यूरोबिलिनोजेन-डिपस्टिक Yes No

RDT Rapid diagnostic test-malaria and or Microscopy-Thick and thin smear Yes No

आरडीटी रैपिड डायग्नोस्टिक टेस्ट-मलेरिया और/या माइक्रोस्कोपी-थिक और थिन स्मीयर

Test tube-based turbidity tests Yes No

टेस्‍ट ट्यूब बेस्‍ड ट्यूबीडिटी टेस्‍ट्स

Thyroid Stimulating Hormone (TSH) Yes No

थायराइड स्‍टीमुलेटिंग होर्मोन (टीएसएच)

**List the readings upon which you will immediately refer the pregnant women to a specialist?**

**वह रीडिंग बताएं जिनके आधार पर आप गर्भवती महिलाओं को तुरंत स्‍पेशलिस्‍ट के पास भेजेंगे?**

If BP reads: {>, <} _____mm of Hg

यदि बीपी है: {>, <} _____mm of Hg

If Bilrubin levels are: {>, <} _____ mg per dl

यदि बिलीरुबिन का स्तर है: {>, <} _____ mg per dl

If serum creatinine levels are: {>, <} _____ mg per dl

यदि सीरम क्रिएटिनिन का स्तर है: {>, <} _____ mg per dl

Pallor and breathlessness with RR: {>, <} _____ per minute

आरआर के साथ पीलापन और सांस फूलना: {>, <} _____ प्रति मिनट

Pallor and breathlessness with PR: {>, <} _____ per minute

पीआर के साथ पीलापन और सांस फूलना: {>, <} _____ प्रति मिनट

Hb levels in third trimester: {>, <} _____ Gms per dl

तीसरी ट्राइमेस्टर में एचबी स्तर: : {>, <} _____ Gms per dl

FBS levels: {>, <} _____ mg per dl

एबीएस का स्‍तर: {>, <} _____ mg per dl

RBS levels: {>, <} _____ mg per dl

आरबीएस का स्‍तर: {>, <} _____ mg per dl

HbA1C: {>, <} _____ %

Malaria treatment patient with Blood sugar level: {>, <} _____ mg per dl

मलेरिया उपचार रोगी ब्‍लड शुगर के स्तर के साथ: {>, <} _____ mg per dl

Malaria treatment patient with Systolic blood pressure: {>, <} _____ mm of Hg

सिस्टोलिक ब्‍लड प्रेशर वाले मलेरिया उपचार रोगी: {>, <} _____ mm of Hg

**Share your perception on the following statements.**

**निम्‍नलिखित वाक्‍यों पर अपनी राय को साझा करें।**

I have good knowledge and skills to handle **ALL** high risk conditions during the antenatal period

मेरे पास प्रसवपूर्व अवधि के दौरान सभी उच्च जोखिम वाली समस्‍याओं को संभालने के लिए अच्छा ज्ञान और कौशल है

1. Yes, Can handle well all high-risk conditions during pregnancy

हां, गर्भावस्था के दौरान सभी उच्च जोखिम वाली समस्‍याओं को अच्छी तरह से संभाल सकता हूँ

1. Somewhat could handle partly some of the high-risk conditions during pregnancy

गर्भावस्था के दौरान कुछ हद तक उच्च जोखिम वाली समस्‍याओं को संभाला जा सकता है

1. No, it’s difficult to handle any of the high-risk conditions during pregnancy

नहीं, गर्भावस्था के दौरान किसी भी उच्च जोखिम वाली समस्‍याओं को संभालना मुश्किल है

I think all women with any risk factor should be referred to specialist right away during antenatal period

मेरा मानना ​​है कि किसी भी जोखिम के कारक वाली सभी महिलाओं को प्रसवपूर्व अवधि के दौरान तुरंत स्‍पेशलिस्‍ट के पास भेजा जाना चाहिए

1. Agree

सहमत

1. Neither agree or nor disagree

ना तो सहमत और न ही असहमत

1. Disagree

असहमत

Women with high- risk-conditions during ANC period to a great extent could be managed at the PHC level before they are referred to designated facilities or specialists

एएनसी अवधि के दौरान उच्च जोखिम वाली समस्‍याओं से पीडि़त महिलाओं का नामित सुविधाओं या स्‍पेशलिस्‍ट के पास भेजे जाने से पहले, काफी हद तक पीएचसी स्तर पर इलाज किया जा सकता है

1. Agree

सहमत

1. Neither agree or nor disagree (not sure)

ना तो सहमत और न ही असहमत (पक्‍का नहीं)

1. Disagree

असहमत

There is a need to improve my knowledge and/or skill for managing high risk pregnancies at the PHC level

1. Agree

सहमत

1. Neither agree or nor disagree (not sure)

ना तो सहमत और न ही असहमत (पक्‍का नहीं)

1. Disagree

असहमत

Sometimes, I am not confident, whether pregnant woman with complications or high risk factors should be referred to AH/CHC/district hospital or higher tertiary institutions straight away.

कभी-कभी, मुझे यह समझ में नहीं आता कि जटिलताओं या उच्च जोखिम वाले कारकों से पीडि़त गर्भवती महिला को सीधे एएच/सीएचसी/जिला अस्पताल या उच्चतर टर्शरी संस्थानों में भेजा जाना चाहिए या नहीं।

1. Agree Depends upon severity

सहमत

1. Neither agree or nor disagree (not sure)

ना तो सहमत और न ही असहमत (पक्‍का नहीं)

1. Disagree

असहमत

It is NOT medical officer’s responsibility to know the status of referrals and follow-up of all women registered with my PHC.

मेरे पीएचसी में पंजीकृत सभी महिलाओं के रेफरल और फॉलो-अप की स्थिति जानना मेडिकल ऑफिसर की जिम्मेदारी नहीं है।

1. Agree

सहमत

1. Neither agree or nor disagree (not sure)

ना तो सहमत और न ही असहमत (पक्‍का नहीं)

1. Disagree

असहमत

**Current Knowledge platforms**

**मौजूदा नॉलेज प्‍लेटफॉर्म**

What do you do if you have doubts related to managing a pregnancy case?[Tick all applicable answers]

यदि गर्भावस्‍था के मामले को प्रबंधित करने के संबंध में आपको कोई संदेह है तो आप क्‍या करते हैं?[लागू होने वाले सभी जवाबो को टिक करें]

1. Reach out to peer doctors

साथी डॉक्‍टर्स से संपर्क करना

1. Reach out to the specialist
2. स्‍पेशलिस्‍ट से संपर्क करना
3. Reach out to college professors
4. कॉलेज प्रोफेसर्स से संपर्क करना
5. Browse the internet

इंटरनेट ब्राउज करना

1. Refer the guidelines material/books

गाइडलाइन मटीरियल/किताबों को देखना

**Access and use of Technology**

**टेक्‍नोलॉजी तक पहुंच और इस्‍तेमाल**

How comfortable are you to use personal mobile phone for official training programmes?

आधिकारिक ट्रेनिंग कार्यक्रमों के लिए आप अपना निजी मोबाइल फोन का इस्‍तेमाल करने में कितना सहज होते हैं?

1. Very comfortable

बहुत सहज

1. Not so comfortable

बहुत सहज नहीं

1. Uncomfortable

असहज

Which one do you use more frequently?

आप किसे ज्‍यादा अक्‍सर इस्‍तेमाल करते हैं?

1. SMS

एसएमएस

1. WhatsApp

वॉट्सऐप

1. Other Chatting apps (telegram, Signal)

अन्‍य चैटिंग ऐप्‍स (टेलीग्राम, सिग्‍नल)

1. Both SMS and WhatsApp

एसएमएस और वॉट्सऐप दोनों

How would you assess the internet connectivity when you are at the PHC?

जब आप पीएचसी में हों तो आप इंटरनेट कनेक्टिविटी का मूल्‍यांकन कैसे करेंगे?

- - 1. Yes, always good connectivity

हां, हमेशा अच्‍छी कनेक्‍टीविटी

- - 1. No, poor connectivity

नहीं, खराब कनेक्टिविटी

- - 1. Sometime good and other times poor connectivity

कभी-कभी अच्‍छी और अन्‍य समय खराब कनेक्टिविटी

Do you at the PHC level, currently screen for the below mentioned conditions and track the improvements (analyse them in your meetings with ANMS and supervisors) through Physical and lab investigation indicators for various high-risk conditions identified in any ANC woman?

क्या आप, वर्तमान में पीएचसी स्तर पर नीचे दी गई समस्‍याओं की जांच करते हैं और किसी भी एएनसी महिला में पहचानी गई विभिन्न उच्च जोखिम वाली समस्‍याओं स्थितियों के लिए शारीरिक और लैब इन्‍वेस्‍टीगेशन के संकेतकों के माध्यम से सुधारों को ट्रैक करते हैं (एएनएमएस और सुपरवाइजर के साथ अपनी मीटिंग्‍स में उनका विश्लेषण करते हैं)?

- Anaemia levels Yes N0 Sometimes
- एनीमिया के स्‍तर हां नहीं कभी-कभी
- Hypertension Yes N0 Sometimes

हाइपरटेंशन हां नहीं कभी-कभी

- Gestational diabetes Yes N0 Sometimes
- गर्भकालीन डायबिटीज हां नहीं कभी-कभी
- Antepartum Haemorhage Yes N0 Sometimes
- प्रसवपूर्व रक्तस्राव हां नहीं कभी-कभी
- Pregnancy with previous LSCS Yes N0 Sometimes
- पहले भी एलएससीएस के साथ गर्भावस्था हां नहीं कभी-कभी
- Decreased foetal movements/ IUD Yes N0 Sometimes
- भ्रूण की गतिशीलता में कमी/आईयूडी हां नहीं कभी-कभी
- Preterm labour/ PPROM Yes N0 Sometimes
- समय से पहले प्रसव पीड़ा/ पीपीआरओएम हां नहीं कभी-कभी
- Bleeding before 20 week Yes N0 Sometimes

20 सप्‍ताह पहले रक्‍तस्राव हां नहीं कभी-कभी

- Pregnancy with heart disease Yes N0 Sometimes

हृदय रोग के साथ गर्भावस्था हां नहीं कभी-कभी

- Pregnancy with IUGR Yes N0 Sometimes
- आईयूजीआर के साथ गर्भावस्था हां नहीं कभी-कभी
- Twin pregnancy Yes N0 Sometimes
- जुड़वां बच्‍चों वाली गर्भावस्था हां नहीं कभी-कभी
- Post- dated pregnancy Yes N0 Sometimes
- पोस्‍ट डेटेड गर्भावस्था हां नहीं कभी-कभी
- Fever in pregnancy Yes N0 Sometimes
- गर्भावस्‍था में बुखार हां नहीं कभी-कभी
- HIV in pregnant Yes N0 Sometimes

गर्भावस्‍था में एचआईवी हां नहीं कभी-कभी

- Epilepsy in pregnancy Yes N0 Sometimes

गर्भावस्था में मिर्गी रोग हां नहीं कभी-कभी

If all the health providers (ANM, MO, specialist) are able to access the health records of pregnant women, do you think at the PHC level management and tracking of all high risk pregnancies could be more effective ?

यदि सभी स्वास्थ्य प्रदाता (एएनएम, एमओ, स्‍पेशलिस्‍ट) की गर्भवती महिलाओं के हेल्‍थ रिकॉर्ड तक पहुंच हों, तो क्या आपको लगता है कि पीएचसी स्तर पर सभी उच्च जोखिम वाली गर्भावस्थाओं का उपचार और ट्रैकिंग अधिक प्रभावी तरीके से हो सकती है?

- - 1. Yes, very useful

हां, बहुत उपयोगी

- - 1. Not sure, can’t say

पक्‍का नहीं, कह नहीं सकते

- - 1. No, not useful

नहीं, उपयोगी नहीं

Given your current responsibilities, how much time could you spend for self-learning about high-risk pregnancies **in one week** using your mobile phone?

अपनी मौजूदा जिम्मेदारियों को देखते हुए, आप अपने मोबाइल फोन का इस्‍तेमाल करके एक सप्ताह में उच्च जोखिम वाली गर्भावस्थाओं के बारे में खुद सीखने के लिए कितना समय दे सकते हैं?

1. One hour

एक घंटा

1. Two hours

2 घंटे

1. Three hours

तीन घंटे

1. More than three hours

3 घंटे से अधिक

1. No time to spend

खर्च करने के लिए समय नहीं है

**Checklist of 15 questions for each HRP**

**प्रत्‍येक एचआरपी के लिए 15 प्रश्‍नों की चेकलिस्‍ट**

**Anemia एनीमिया**

1). Anemia in pregnancy is defined as:

गर्भावस्था में एनीमिया को इस प्रकार परिभाषित किया जाता है

Hb levels < 11gm/dl =1

एचबी स्तर < 11 gm/dl =1

Hb levels < 12gm/dl =2

एचबी लेवल < 12 gm/dl =2

Hb levels < 13 gm/dl =3

एचबी लेवल < 13 gm/dl =3

None =4

कोई नहीं

2). Correct classification of anemia in pregnancy is:

गर्भावस्था में एनीमिया का सही वर्गीकरण इस प्रकार हैः

Mild: Hb 10-10.9 g/dl, Moderate: Hb 7-9.9g/dl, Severe: <7 =1

हल्‍का: एचबी 10-10.9 g/dl, मध्‍यम: एचबी 7-9.9g/dl, गंभीर:

Mild: Hb 11-11.9 g/dl, Moderate: Hb 8- 10g/dl, Severe: <8 =2

हल्‍का: एचबी 11-11.9 g/dl, मध्‍यम: एचबी 8- 10g /dl, गंभीर:

Mild: Hb 11-12.9 g/dl, Moderate: Hb 8- 10.9g/dl, Severe: <8 =3

हल्‍का: एचबी 11-12.9 g/dl, मध्‍यम: एचबी 8- 10.9g /dl, गंभीर:

None =4

कोई नहीं

3). What are the ways of preventing anemia in pregnancy?

गर्भावस्था में एनीमिया को रोकने के क्या तरीके हैं?

Deworming =1

कृमिनाशक

Iron and Folic acid supplementation =2

आयरन और फोलिक एसिड सप्‍लीमेंटेशन

Increase intake of iron-rich food and vitamin C rich-foods =3

आयरन युक्त भोजन और विटामिन C युक्त भोजन का सेवन बढ़ाना

All of the above =4

उपरोक्‍त सभी

4). What is the recommended dose of iron and folic acid tablets in pregnant women with mild anemia?

हल्के एनीमिया से पीड़ित गर्भवती महिलाओं के लिए आयरन और फोलिक एसिड की गोलियों की सुझाई गई डोज क्या है?

Two tablets of iron and folic acid (100 mg elemental iron and 500 mcg folic acid) daily for 6 months =1

आयरन और फोलिक एसिड की दो टैबलेट्स (100 mg एलीमेंट्स आयरन और 500 mcg फोलिक एसिड) 6 महीने तक प्रतिदिन

One tablet of iron and folic acid (100 mg elemental iron and 500 mcg folic acid) daily for 6 months =2

आयरन और फोलिक एसिड की एक टैबलेट्स (100 mg एलीमेंट्स आयरन और 500 mcg फोलिक एसिड) 6 महीने तक प्रतिदिन

Only iron tablet =3

केवल आयरन की टैबलेट

None =4

कोई नहीं

5). Can anemia in pregnant woman affect the baby?

क्या गर्भवती महिला में एनीमिया बच्चे को प्रभावित कर सकता है?

Yes, it can cause preterm, low birth weight babies =1

हाँ, इससे समय से अपरिपक्व, कम वजन वाले बच्चे पैदा हो सकते हैं

No, it can only lead to symptoms in the mother =2

नहीं, इससे केवल माँ में ही लक्षण उत्पन्न हो सकते हैं

No, it can only lead to complications during delivery =3

नहीं, इससे केवल प्रसव के दौरान ही जटिलताएं उत्पन्न हो सकती हैं

None of the above =4

उपरोक्‍त सभी

6). Parenteral iron therapy is recommended in which of the following conditions:

निम्नलिखित में से किस स्थिति में पैरेंट्रल आयरन थेरेपी की सिफारिश की जाती है:

Mild anemia in first trimester =1

प्रथम ट्राइमेस्टर में हल्का एनीमिया

Moderate anemia in second trimester without a trial of oral IFA tablets =2

खाने वाली आईएफए की टैबलेट के ट्रायल के बिना दूसरे ट्राइमेस्टर में मॉडरेट एनीमिया

Severe anemia in third trimester with breathlessness =3

सांस लेने में समस्‍या के साथ तीसरे ट्राइमेस्टर में गंभीर एनीमिया

Pregnant woman with moderate anemia in second trimester not tolerating oral iron. =4

दूसरे ट्राइमेस्टर में मध्यम एनीमिया से पीड़ित गर्भवती महिला जो खाने वाले आयरन को सहन नहीं कर पाती

7). What are the common symptoms with which a patient of anemia may present?

एनीमिया के रोगी में कौन से सामान्य लक्षण दिखाई दे सकते हैं?

Fatigue, palpitations, breathlessness, giddiness =1

थकान, घबराहट, सांस फूलना, चक्कर आना

Cough, blood in sputum, fever =2

खांसी, बलगम में खून, बुखार

Nausea, vomiting, diarrhea =3

मतली, उल्टी, दस्त

Headache, loss of vision, vomiting =4

सिरदर्द, नज़र कमजोर होना, उल्टी

8). What are the signs of anemia on clinical examination?

चिकित्सीय जांच में एनीमिया के लक्षण क्या हैं?

Clubbing =1

क्लबिंग

Pallor =2

पीलापन

Pedal oedema =3

पेडल इडीम

All of the above =4

उपरोक्‍त सभी

9). Estimation of hemoglobin levels in pregnancy should be done:

गर्भावस्था में हीमोग्लोबिन के स्तर का आकलन किया जाना चाहिए:

At 12, 24 and 36 weeks =1

12, 24 और 36 सप्ताह पर

In the first and last visit =2

पहली और आखिरी विजिट में

At all ANC visits =3

सभी एएनसी विजिट में

None =4

कोई नहीं

10). The correct formula for calculating the dose of IV iron is:

IV आयरन की डोज की गणना के लिए सही सूत्र है:

Required iron dose (mg) = [2.4 X (target Hb (11) -actual Hb) X pre-pregnancy weight (kg)] +1000 mg for replenishment of stores =1

आयरन की आवश्यक डोज (mg) = [2.4 X (टारगेट एचबी (11) -वास्तविक एचबी) X गर्भावस्था पूर्व वजन (किलोग्राम)] +भंडार की पुनःपूर्ति के लिए 1000 mg

Required iron dose (mg) = [2.4 X (target Hb (11) -actual Hb) X pre-pregnancy weight (kg)] +500 mg for replenishment of stores =2

आयरन की आवश्यक डोज (mg) = [2.4 X (टारगेट एचबी (11) -वास्तविक एचबी) X गर्भावस्था पूर्व वजन (किलोग्राम)] +भंडार की पुनःपूर्ति के लिए 500 mg

No calculation is needed, we give 5 injections of 200 mg on alternate days =3

किसी गणना की आवश्यकता नहीं है, हम हर दूसरे दिन 200 mg के 5 इंजेक्शन देते हैं

None of the above =4

उपरोक्‍त में से कोई नहीं

11). 26 year old primigravida at 22 weeks POG was tested to have a Hb of 10g/dl, mark the correct statement about her management plan:

26 वर्षीय प्राइमिग्रेविडा का 22 सप्ताह के पीओजी का टेस्‍ट किया गया जिसमें Hb 10g/dl पाया गया, उसके प्रबंधन योजना के बारे में सही वाक्‍य को मार्क करें:

She is low risk so can be given usual care =1

उसे कम जोखिम है इसलिए उसे सामान्य देखभाल दी जा सकती है

She has mild anemia, needs to be managed as high-risk pregnancy =2

उसे हल्का एनीमिया है, उसका उच्च जोखिम वाली गर्भावस्था के रूप में उपचार किया जाना चाहिए

She has severe anemia, needs to be referred to tertiary care center =3

उसे गंभीर एनीमिया है, उसे टर्शर केयर सेंटर में रेफर करने की आवश्यकता है

None =4

कोई नहीं

12). Steps to manage anaphylactic reaction to I.V iron include:

IV आयरन के लिए एनाफाइलैक्टिक प्रतिक्रिया के प्रबंधन के लिए निम्नलिखित कदम उठाए जा सकते हैं:

Stop the injection immediately =1

इंजेक्शन तुरन्त बंद करें

Give Inj. Hydrocortisone 200 mg IV =2

इंजेक्शन हाइड्रोकार्टिसोन 200 mg IV दें

0.5 mg of 1:1000 adrenaline IM =3

1:1000 एड्रेनालाईन IM का 0.5 mg

All of the above =4

उपरोक्‍त सभी

13). A 25-year old Primigravida at 38 weeks POG comes with Hb of 6.5gm/dl. Her vitals are stable and there are no other complaints. How will you manage?

25 वर्षीय प्राइमिग्रेविडा का 38 सप्ताह का पीओजी एचबी 6.5gm/dl आता है। उसकी वाइटल्‍स स्थिर है और कोई अन्य शिकायत नहीं है। आप कैसे प्रबंधित करेंगे?

Refer to higher center =1

उच्‍च सेंटर पर रेफर करेंगे

Start IV iron =2

IV आयरन शुरू करेंगे

Give her oral iron =3

उसे आयरन की गोली देंगे

Prepare for delivery =4

डिलीवरी की तैयार करेंगे

14). What precautions will you ask the pregnant woman to take while taking iron tablets:

आयरन की टैबलेट्स लेते समय आप गर्भवती महिला को क्या सावधानियां बरतने के लिए कहेंगे:

Keep a gap of 2hrs between iron and calcium tablets =1

आयरन और कैल्शियम की टैबलेट्स के बीच 2 घंटे का अंतर रखें

Do not take iron tablets with milk/tea/coffee =2

आयरन की टैबलेट्स दूध/चाय/कॉफी के साथ न लें

Taking iron with lemon water may increase its absorption =3

नींबू पानी के साथ आयरन लेने से इसका अवशोषण बढ़ सकता है

All of the above

उपरोक्‍त सभी

15). Which of the following cases should be referred to higher center:

इनसे से कौन से केसेस को हायर सेंटर को भेजा जाना चाहिए

All pregnant women with Hb < 7-9.9 g/dl =1

सभी गर्भवती महिलाएं जिनका एचबी < 7-9.9 g/dl =1 है

Any pregnant woman with pallor and breathlessness (RR >24/min) and or tachycardia (PR>100 bpm) =2

कोई भी गर्भवती महिला जिसका शरीर पीला पड़ा हो और सांस फूल रही हो (RR >24/मिनट) और या तीव्र हृदयगति (पीआर>100 bpm) =2

All pregnant women who develop vomiting/severe nausea/diarrhea/severe constipation with IFA =3

सभी गर्भवती महिलाएं जिनमें आईएफबी =3 के साथ उल्टी/गंभीर मतली/दस्त/गंभीर कब्ज की समस्या होती है

All of the above =4

उपरोक्‍त सभी

**Pregnancy Induced Hypertension (PIH)**

**गर्भावस्था से प्रेरित हाइपरटेंशन (पीआईएच)**

1). Hypertension in pregnancy is defined as?

गर्भावस्था में हाइपरटेंशन को इस प्रकार परिभाषित किया जाता है?

Systolic BP >=90 mm of Hg; diastolic >=140 mm of Hg =1

सिस्‍टोलिक बीपी >=90 mm of Hg; डायस्टोलिक >=140 mm of Hg =1

Systolic BP <120 mm of Hg; diastolic <80 mm of Hg =2

सिस्‍टोलिक बीपी <120 mm of Hg; डायस्टोलिक <80 mm of Hg =2

Systolic BP >=140 mm of Hg; diastolic>=90mm of Hg =3

सिस्‍टोलिक बीपी >=140 mm of Hg; डायस्टोलिक >=90mm of Hg =3

None of the above =4

उपरोक्‍त सभी

2). Which of the following is a warning symptom of hypertension in pregnancy?

इनमें से कौन सा गर्भावस्था में हाइपरटेंशन की चेतावनी का लक्षण है?

Generalized oedema which is non-dependent =1

सामान्यीकृत एडिमा जो गैर-निर्भर है =1

Frequent urination =2

बार-बार पेशाब आना =2

Loss of appetite =3

भूख न लगना =3

All of the above =4

उपरोक्‍त सभी=4

None of the above =5

इनमें से कोई नहीं

3). Risk factors for preeclampsia are:

प्रीक्लेम्पसिया के जोखिम कारक हैं:

High B.P in previous pregnancy =1

पिछली गर्भावस्था में हाई बीपी =1

Age > 35 years =2

आयु 25 वर्ष से कम=2

First pregnancy =3

पहली गर्भावस्‍था

All of the above =4

उपरोक्‍त सभी

4). All of the following may be used in pregnancy associated hypertension except

इनमें से सभी का इस्‍तेमाल गर्भावस्था से जुड़े हाइपरटेंशन में किया जा सकता है, सिवाय

Nifedipine =1

निफेडिपिन =1

Captopril =2

कैप्टोप्रिल =2

Methyldopa =3

मेथिलडोपा =3

Hydralazine = 4

हाइड्रैलाज़िन

5). What are the signs of hypertension on clinical examination during ANC visit?

एएनसी विजिट के दौरान क्‍लीनिकल परीक्षण में हाइपरटेंशन के लक्षण क्या हैं?

Excessive weight gain =1

अत्यधिक वजन बढ़ना =1

BP more than 140/90 mm of Hg =2

बीपी 140/90 mm of Hg से अधिक होना =2

Non-dependent pedal oedema =3

नॉन-डिपेंडेंट पेडल इडीम=3

All of the above =4

उपरोक्‍त सभी

6). If a pregnant woman presents with eclampsia, what dose of Inj. Magnesium sulphate would you give her?

यदि कोई गर्भवती महिला एक्लेम्पसिया से पीड़ित है, तो आप उसे इंजेक्शन मैग्नीशियम सल्फेट की कितनी डोज देंगे?

20ml of 20% solution of magnesium sulphate slow IV and 10ml of 50% solution of magnesium sulphate in each buttock =1

प्रत्येक नितंब में मैग्नीशियम सल्फेट 20% सलूशन के स्‍लो IV का 20ml और मैग्नीशियम सल्फेट के 50% सलूशन का 10ml =1

10ml of 20% solution of magnesium sulphate slow IV and 20ml of 50% solution of magnesium sulphate in each buttock =2

प्रत्येक नितंब में मैग्नीशियम सल्फेट 20% सलूशन के स्‍लो IV का 10ml और मैग्नीशियम सल्फेट के 50% सलूशन का 20ml =1

Either a or b =3

या तो a या b

None =4

कोई नहीं

7). What all will you monitor in a patient on Inj. Magnesium Sulphate?

मैग्नीशियम सल्फेट इंजेक्शन लेने वाले रोगी में आप किन-किन बातों पर नजर रखेंगे?

Deep tendon reflexes =1

डीप टेंडन रिफ्लेक्‍सेस

Urine output =2

यूरीन आउटपुट

Respiratory rate =3

श्‍वसन दर

All of the above =4

उपरोक्‍त सभी

8). In which of the following cases will you refer a pregnant woman with preeclampsia to higher center:

इनमें से किस मामले में आप प्रीक्लेम्पसिया से पीड़ित गर्भवती महिला को हायर सेंटर पर रेफर करेंगे:

Fetal growth retardation =1

भ्रूण के वृद्धि का धीमा होना

Reduced urinary output =2

कम पेशाब होना

Proteinuria 1+ =3

प्रोटीनुरिया 1+

a and b =4

a और b

9). What are the warning symptoms a patient of preeclampsia may present with?

प्रीक्लेम्पसिया के रोगी में कौन से चेतावनी लक्षण दिखाई दे सकते हैं?

Fatigue, giddiness, pallor =1

थकान, चक्कर आना, पीलापन =1

Cough, blood in sputum, fever =2

खांसी, बलगम में खून, बुखार =2

Nausea, vomiting, diarrhea =3

मतली, उल्टी, दस्त =3

Headache, blurry vision, vomiting =4

सिरदर्द, धुंधला दिखाई देना, उल्टी

10). Blood pressure levels in pregnancy should be checked:

गर्भावस्था में ब्‍लड प्रेशर के स्तर की जाँच की जानी चाहिए:

At 12, 24 and 36 weeks =1

12, 24 और 36 सप्ताह पर =1

In the first and last visit =2

पहली और आखिरी विजिट में =2

At all ANC visits =3

सभी एएनसी विजिट में=3

None =4

कोई नहीं

11). Out of the given options, choose the correct nutritional advice that should be given to a woman with gestational hypertension:

दिए गए विकल्पों में से, सही पोषण संबंधी सलाह चुनें जो गर्भावस्‍था में हाई ब्‍लड प्रेशर वाली महिला को दी जानी चाहिए:

Low carbohydrate, high fat diet =1

कम कार्बोहाइड्रेट, उच्च फैट वाला आहार =1

Diet rich in proteins =2

प्रोटीन से भरपूर आहार =2

Low salt diet =3

कम नमक वाला आहार

None of the above =4

उपरोक्‍त में से कोई नहीं

12). 26 yr., prim gravida at 22 weeks has BP reading 166/112 mm Hg, mark the correct statement about her management plan:

26 वर्ष, 22 सप्ताह में प्राइमिग्रेविडा का बीपी 166/112 mm Hg है, उसकी प्रबंधन योजना के बारे में सही वाक्‍य को मार्क करें:

She is low risk so can be given usual care. =1

उसे कम जोखिम है इसलिए उसे सामान्य देखभाल दी जा सकती है। =1

She has hypertension, can be managed with medications alone. =2

उसे हाइपरटेंशन है, केवल दवाओं से उसका इलाज किया जा सकता है। =2

She has possibility of eclampsia, give necessary medications and refer to tertiary care center. =3

उसे एक्लेम्पसिया होने की संभावना है, आवश्यक दवाएं दें और टर्शरी मेडिकल सेंटर में रेफर करें। =3

None =4

कोई नहीं

13). What would be your plan for 29 yrs, gravida 2, with normal BP but history of hypertension during previous pregnancy?

29 वर्ष की गर्भवती महिला, जिसका बीपी सामान्य है, लेकिन पिछली गर्भावस्था के दौरान हाइपरटेंशन का इतिहास है, उसके लिए आपकी क्या योजना होगी?

Monitor BP at every ANC visit =1

प्रत्येक एएनसी विजिट पर बीपी की जांच करें =1

Counselling on warning signs of gestational hypertension =2

गर्भावधि संबंधी हाइपरटेंशन के चेतावनी संकेतों पर परामर्श दें =2

Start her on Cap. Nifedipine 10 mg or Tab. Labetalol 200 mg =3

उसे कैप्‍सूल निफेडिपिन 10 mg या टैबलेट लेबेटालोल 200 मिलीग्राम देना शुरू करें =3

Both a & b =4

a और b दोनों =4

All of the above =5

उपरोक्‍त सभी

14). A 29 yr. Primigravida at 36 wks POG with BP of 150/110 mmHg, presents with history of headache, blurring of vision and vomiting. She is not in labour. How will you manage?

29 साल की प्राइमिग्रेविडा जिसका 36 सप्ताह के पीओजी पर 150/110 mmHg का बीपी है, और जिसका सिरदर्द, नज़र का धुंधलापन और उल्टी का इतिहास है। उसे प्रसव पीड़ा नहीं हो रही है। आप कैसे प्रबंधन करेंगे?

Give the loading dose of Inj Magnesium sulphate and refer to higher center. =1

इंजेक्शन मैग्नीशियम सल्फेट की लोडिंग खुराक देंगे और हायर सेंटर को रेफर करेंगे। =1

Give the loading dose of Inj Magnesium sulphate and start her on a anti-hypertensives and transport to higher center. =2

इंजेक्शन मैग्नीशियम सल्फेट की लोडिंग खुराक देंगे और उसे एंटी-हाइपरटेंसिव देना शुरू करेंगे और हायर सेंटर ले जाएंगे। =2

Start her on antihypertensives and pain killers =3

उसे एंटीहाइपरटेंसिव और पेन किलर देना शुरू करेंगे=3

Start her on antihypertensives and monitor till BP comes down =4

उसे एंटीहाइपरटेंसिव दवाएं देना शुरू करें और बीपी कम होने तक निगरानी करेंगे

15). Which of the following cases should be referred to higher center:

इनमें से कौन से मामले को हायर सेटर को भेजा जाना चाहिए:

All pregnant women with BP >= 160/110 mm Hg =1

बीपी >= 160/110 mm Hg वाली सभी गर्भवती महिलाएं

Any pregnant woman with generalized oedema, BP >140/90mm Hg and proteinuria (2+) =2

कोई भी गर्भवती महिला जिसका सामान्यीकृत इडीम, बीपी >140/90mm Hg और प्रोटीनुरिया (2+) हो

All pregnant women who develop eclampsia-seizure/blurry vision/high BP =3

सभी गर्भवती महिलाएं जिनमें एक्लेम्पसिया-दौरा/धुंधली नज़रे/हाई बीपी है =3

All of the above =4

उपरोक्‍त सभी

**Ante Partum Haemorrhage (APH):**

**प्रसवपूर्व रक्तस्राव (एपीएच) :**

1). Antepartum hemorrhage(APH) is defined as

प्रसवपूर्व रक्तस्राव (एचपीएच) को इस प्रकार परिभाषित किया जाता है

Bleeding from or in to the genital tract, occurring after 20 weeks of pregnancy and prior to the birth of the baby =1

गर्भावस्था के 20 सप्ताह के बाद और बच्चे के जन्म से पहले जननांग मार्ग से या उसके अन्दर रक्तस्राव होना, =1

Bleeding from or in to the genital tract, occurring from 24+0 weeks of pregnancy and prior to the birth of the baby =2

गर्भावस्था के 24+0 सप्ताह के बाद और बच्चे के जन्म से पहले जननांग मार्ग से या उसके अन्दर रक्तस्राव होना, =2

Bleeding from or in to the genital tract, occurring before 20 weeks of pregnancy and prior to the birth of the baby =3

गर्भावस्था के 24+0 सप्ताह के पहले और बच्चे के जन्म से पहले जननांग मार्ग से या उसके अन्दर रक्तस्राव होना, =3

None of the above =4

उपरोक्त में से कोई नहीं

2). Which of the following is true for APH?

इनमें से कौन सा एपीएच के लिए सही है?

A little bleeding after 20 weeks is normal =1

20 सप्ताह के बाद थोड़ा रक्तस्राव होना सामान्य है =1

It is always associated with pain abdomen =2

यह हमेशा पेट दर्द से जुड़ा होता है=2

It is always painless =3

यह हमेशा दर्द रहित होता है =3

None of the above =4

उपरोक्त में से कोई नहीं

3). Causes of APH are:

एपीएच के कारण हैं:

Placenta previa =1

प्लेसेंटा प्रीविया

Abruptio placentae =2

एब्रुपियो प्लेसेंटा

Local causes =3

स्थानीय कारण =3

All of the above =4

उपरोक्‍त सभी

4). 29 yr Primigravida at 34 weeks POG presents with c/o mild bleeding with pain abdomen for 2hrs. On examination her pulse rate is 90/min and BP is 150/90 mmHg. On P/A uterus corresponds to 36 weeks and tender, FHS 110bpm and her pad is only mildly stained. What is your diagnosis?

29 वर्षीय प्राइमिग्रेविडा जिसका 34 सप्ताह का पीओजी है उसे 2 घंटे तक पेट में दर्द के साथ हल्का रक्तस्राव होता है। जांच करने पर उसका पल्‍स रेट 90/मिनट और बीपी 150/90 mmHg है। पी/ए पर गर्भाशय 36 सप्ताह का है और कोमल है, एफएचएस 110bpm है और उसका पैड केवल हल्का दागदार है। आपका डायग्‍नोसिस क्‍या है?

Placenta Previa =1

प्लेसेंटा प्रेविया=1

Abruptio placentae =2

एब्रुपियो प्लेसेंटा=2

Uterine rupture =3

गर्भाशय का टूटना=3

None of the above =4

उपरोक्त में से कोई नहीं

5). A pregnant woman with low lying placenta

एक गर्भवती महिला जिसकी नाल नीचे की ओर झुकी हुई है

Can be delivered at a PHC =1

पीएचसी पर डिलीवरी की जा सकती है

Can be delivered at a subcenter =2

उपकेंद्र पर डिलीवरी की जा सकती है

Should be delivered at a DH/tertiary care center where a specialist is available =3

डीएच/टर्शरी केयर सेटर में डिलीवरी कराई जानी चाहिए जहां स्‍पेशलिस्‍ट उपलब्ध हो =3

Not sure =4

पक्‍का नहीं

6). A pregnant woman comes bleeding, her pulse rate is 100bpm, BP is 110/70 mmHg with no other signs and symptoms. How will you classify the degree of shock?

एक गर्भवती महिला को रक्तस्राव हो रहा है, उसकी पल्‍स रेट 100bpm है, बीपी 110/70 mmHg है और कोई अन्य लक्षण या संकेत नहीं है। आप सदमे के स्‍तर को कैसे वर्गीकृत करेंगे?

Class I =1

क्‍लास I =1

Class II =2

क्‍लास II =2

Class III =3

क्‍लास III =3

None =4

कोई नहीं

7). Ideal fluid for replacement in a patient in shock is:

सदमे में किसी रोगी के रिप्‍लेसमेंट के लिए आदर्श फ्लूइड है:

5% dextrose =1

5% डेक्सट्रोज =1

Normal Saline =2

सामान्य सलाइन =2

Ringer lactate =3

रिंगर लैक्टेट =3

Blood =4

ब्‍लड

8). In a patient requiring blood transfusion, what is the ideal component to be transfused:

एक रोगी जिसे ब्‍लड ट्रांसफ्यूजन की आवश्‍यकता है, ट्रांसफ्यूज करने के लिए आदर्श कंपोनेंट क्‍या है

Whole blood =1

पूरा ब्‍लड

Packed red blood cells =2

पैक्‍ड रेड ब्‍लड सेल्‍स

Plasma =3

प्‍लाज्‍मा

None =4

कोई नहीं

9). A 28yr old G3P2L2 with 36 wks POG is brought to you in a semi-conscious state with pulse rate > 100/ min, BP 90/60 mmHg with bleeding P/V for 30 mins. Her clothes are soaked with blood. How will you manage?

28 वर्ष की एक G3P2L2 को 36 सप्ताह के पीओजी के साथ आधी बेहोशी की हालत में आपके पास लाया गया है, जिसकी पल्‍स रेट > 100/मिनट, बीपी 90/60 mmHg है तथा 30 मिनट तक P/V से रक्तस्राव हो रहा है। उसके कपड़े खून से भीगे हुए हैं। आप मामले को कैसे संभालेंगे?

Put a large bore I.V cannula and start fluids =1

एक लार्ज बोर I.V कैनुला डालेंगे और फ्लूइड शुरू करेंगे=1

Take sample for blood grouping and cross matching =2

ब्लड ग्रुपिंग और क्रॉस मैचिंग के लिए सैंपल लेंगे =2

Transport the patient yourself to a tertiary care center =3

रोगी को स्वयं टर्शरी केयर सेंटर ले जाएंगे =3

All of the above =4

उपरोक्‍त सभी

10). How will you counsel a pregnant woman with placenta previa on USG?

आप यूएसजी पर प्लेसेंटा प्रीविया से पीड़ित गर्भवती महिला को कैसे परामर्श देंगे?

Tell her about the warning signs =1

उसे चेतावनी संकेतों के बारे मे बताएंगे

Tell her about the need to register herself at a tertiary care center =2

उसे खुद का टर्शर केयर सेंटर में पंजीकरण कराने की आवश्यकता के बारे में बताएंगे =2

Both A and B =3

A और B दोनों=3

None of the above =4

उपरोक्त में से कोई नहीं

11). While transferring a patient of APH to a tertiary care hospital, following should be kept in mind:

एपीएच के रोगी को टर्शरी केयर हॉस्पिटल में ट्रांसफर करते समय इन बातों को ध्यान में रखा जाना चाहिए:

Send a skilled provider with the woman to ensure an open airway, to deliver first aid if she goes into shock =1

महिला के साथ एक कुशल प्रदाता को भेजें ताकि वायुमार्ग खुला रहे, तथा यदि वह सदमे में चली जाए तो प्राथमिक उपचार दिया जा सके =1

Elevate legs to improve blood supply to vital organs =2

महत्वपूर्ण अंगों में रक्त की आपूर्ति में सुधार करने के लिए पैरों को ऊपर उठाएं =2

Avoid hypothermia- keep her warm =3

हाइपोथर्मिया से बचें- उसे गर्म रखें =3

All of the above =4

उपरोक्‍त सभी

12). Following facilities should be available at any center to ensure safe delivery of a woman with APH:

एपीएच से पीड़ित महिला की सुरक्षित डिलीवरी सुनिश्चित करने के लिए किसी भी केंद्र पर निम्नलिखित सुविधाएं उपलब्ध होनी चाहिए:

Fully operational blood bank =1

पूरी तरह से चालू ब्‍लड बैंक

24 X 7, availability of senior obstetrician, pediatrician and anesthetist =2

24 x 7, सीनियर प्रसूति रोग विशेषज्ञ, बाल रोग विशेषज्ञ एवं एनेस्थेटिस्ट की उपलब्धता =2

Fully operational laboratory =3

पूरी तरह से चालू लेबोरेटरी=3

All of the above =4

उपरोक्‍त सभी

13). Patients with h/o last delivery by LSCS are at increased risk of

एलएससीएस द्वारा पिछली डिलीवरी वाली गर्भवती महिलाओ को इसका खतरा बढ़ जाता है

Placenta previa = 1

प्लेसेंटा प्रिविया

Uterine rupture =2

गर्भाशय का टूटना

Both a & b =3

a और b दोनों

None = 4

कोई नहीं

14). PV examination should not be done in

पीवी परीक्षण नहीं किया जाना चाहिए

Suspected placenta previa =1

संदिग्ध प्लेसेंटा प्रीविया

Suspected abruptio placentae =2

संदिग्ध प्लेसेंटा एब्रप्टियो

Suspected uterine rupture =3

संदिग्ध गर्भाशय का टूटना

Early labor pains =4

जल्‍दी प्रसव पीडा

15). In which of the following conditions the vital signs of the patient are proportional to the visible blood loss?

इनमें से किस स्थिति में रोगी के महत्वपूर्ण संकेत दिखने वाले ब्‍लड लॉस के समानुपाती होते हैं?

Abruptio placentae =1

एब्रुपियो प्लेसेंटा

Placenta previa =2

प्लेसेंटा प्रीविया

Rupture uterus =3

गर्भाशय का टूटना

None of the above =4

उपरोक्त में से कोई नहीं

**Thank you for answering all the questions. We are looking forward to meet you during the training.**

**सभी प्रश्नों का जवाब देने के लिए धन्यवाद. हम ट्रेनिंग के दौरान आपसे मिलने के लिए उत्सुक हैं।**
