# Supplementary material for: Impact of a Multicomponent Intervention to Build Capacity of Public Health Workers to Make Algorithmic Diagnosis and Management of High-Risk Pregnancies in Uttar Pradesh, India: Protocol for a Matched-Control, Before-After, Quasi-Experimental Study With a Mixed Methods Design
Source: JMIR Res Protoc. 2025 Dec 9;14:e74993. doi: 10.2196/74993 (PMC12690279; doi:10.2196/74993)
Supplement: Multimedia Appendix 5 [file resprot-v14-e74993-s005.docx]

**Annexure 5**

**ANM Tool**

**एएनएम टूल**

**Informed Consent Form**

सूचित सहमति फॉर्म

[Baseline Survey to Understand the ANMs Knowledge, Attitude and Practices on High-risk Pregnancy Management in Uttar Pradesh]

[उत्‍तर प्रदेश में हाई-रिस्‍क वाली गर्भावस्‍था के प्रबंधन में एएनएम की जानकारी, दृष्टिकोण और व्‍यवहारों को समझने के लिए बेसलाइन सर्वे]

Dear ANM

प्रिय एएनएम

Greetings to you. ARMMAN in partnership with Government of Uttar Pradesh, is going to capacitate all ANMs to improve their knowledge & skills to diagnose and manage handle high-risk pregnancies. Very soon all ANMs will have training sessions to expose you to the high-risk pregnancy diagnosis and management, using the colour coded protocols developed for six priority high risk conditions identified by the state of Uttar Pradesh. Before ARMMAN initiates the training, it is important to understand the current practices followed by ANMs to diagnose, manage, refer and follow-up done to women with HRPs. Purpose of baseline survey is to understand these aspects.

आपका स्‍वागत है। उत्‍तर प्रदेश में सरकार के साथ पार्टनरशिप में अरमान सभी एएनएम उच्‍च-जोखिम वाली गर्भावस्‍थाओं का पता लगाने और उसे संभालने के लिए उनके ज्ञान और कौशल में सक्षम बनाने जा रहा है। बहुत जल्द ही सभी एएनएम लिए ट्रेनिंग सत्र आयोजित किये जाएंगे, जिसमें आपको उच्च जोखिम वाली गर्भावस्था का पता लगाने और उनके प्रबंधन के बारे में बताया जाएगा, जिसमें उत्तर प्रदेश राज्य द्वारा पहचानी गई छह प्राथमिकता वाली उच्च जोखिम वाली समस्‍याओं के लिए तैयार किये गए कलर कोडेड प्रोटोकॉल का किया जाएगा। इससे पहले की अरमान ट्रेनिंग शुरू करे, एचआरपी से पीड़ित महिलाओं में डायग्‍नोस, प्रबंधन, रेफर करने और फॉलो-अप के लिए एएनएम द्वारा अपनाई जाने वाले मौजूदा तरीकों को समझना महत्वपूर्ण है। बेसलाइन सर्वेक्षण का उद्देश्य इन पहलुओं को समझना है।

Please read the questions and answer the questions that you feel is right. It is okay if you are not aware of some questions. You can say you do not know or skip the question. Purpose of baseline is not to evaluate you. It is only to understand your current understanding and practices pertinent to HRP management.

कृपया प्रश्‍नों को पढें और प्रश्‍नों का वह जवाब दें जो आपको सही लगता है। अगर आपको कुछ प्रश्‍नों के बारे में नहीं पता है तो कोई बात नहीं। आप कह सकते हैं कि आपको नहीं पता या प्रश्‍न को छोड़ सकते हैं। बेसलाइन का उद्देश्‍य आपका मूल्‍यांकन करन नहीं है। यह सिर्फ एचआरपी मैनेजमेंट के संबंध में आपकी मौजूदा समझ और अभ्‍यास को समझने के लिए है।

The survey responses will not be identified with your name or mobile phone number or anything specific with your identity. The responses of all the ANMs will be looked at together to understand the objectives of this survey. We intend to use this information to present to the state authorities, as part of situational assessment of HRP management in the state. The findings of this survey will be disseminated with Government of Uttar Pradesh and to other appropriate audience and/or platforms. Learning from this survey will also feed into designing of training plans, implementation of HRP management program in the state. This survey shall take 30-40 minutes.

सर्वे के जवाबों को आपके नाम या मोबाइल फ़ोन नंबर या आपकी पहचान से जुड़ी किसी भी विशेष चीज़ से पहचाना नहीं जाएगा। इस सर्वे के उद्देश्यों को समझने के लिए सभी एएनएम के जवाबों को एक साथ देखा जाएगा। हम इस जानकारी का इस्‍तेमाल राज्य के अधिकारियों को राज्य में एचआरपी के स्थितिजन्य मूल्यांकन के भाग के रूप में प्रस्तुत करने के लिए करना चाहते हैं। इस सर्वे के निष्कर्षों को उत्तर प्रदेश सरकार और अन्य उपयुक्त दर्शकों और/या प्लेटफार्मों के साथ साझा किया जाएगा। इस सर्वे से प्राप्त जानकारी का इस्‍तेमाल ट्रेनिंग योजनाओं को तैयार करने, राज्य में एचआरपी मैनेजमेंट प्रोग्राम के कार्यान्वयन में भी किया जाएगा। इस सर्वे में 30-40 मिनट का समय लगेगा।

Prior to participating in this survey, please answer the following.

इस सर्वे में भाग लेने से पहले, कृपया निम्‍नलिखित का जवाब दें।

1. Do you agree to take part in this survey?

क्‍या आप इस सर्वे में भाग लेने के लिए सहमत हैं?

1. Yes हां
2. No नहीं
3. Do you understand the purpose of this survey?

क्‍या आप इस सर्वे के उद्देश्‍य को समझती हैं?

1. Yes हां
2. No नहीं

Name of the Interviewer: ____________________

इंटरव्‍यूअर का नाम:

Date: __________________

तारीख:

**Contact** If you have any questions or concerns regarding this interview, please connect with: ARMMAN contact: Dr. Hanimi Reddy Modugu , Email: hanimi@armman.org, Mobile: +91 99118 22445

**संपर्क** यदि आपके पास इस इंटरव्‍यू के संबंध में कोई प्रश्‍न या चिंताएं हैं, तो कृपया निम्‍न से संपर्क करें: अरमान का कॉन्‍टेक्‍ट: डॉ. हनिमी रेड्डी मोडुगु, ईमेल: hanimi@armman.org, मोबाइल: +91 99118 22445

If you have any concerns regarding your rights as a participant, please contact Sigma Research and Consulting (IRB) at [irb.sigma@sigma-india.in](mailto:irb.sigma@sigma-india.in), Phone: 011- 41063450

1. Signature of ANM: _______________________ Date:___________

एएनएम के हस्‍ताक्षर तारीख

**Background information**

**पृष्‍ठभूमि की जानकारी**

Educational qualification: {options – 10^th^, Inter, Degree, _________}

शैक्षिक योग्‍यता{विकल्‍प – 10^वीं^, इंटर,डिग्री _________}

Name of the District currently serving: _______

जिस जिले में अभी सेवा प्रदान कर रही हैं:

Name of the Subcentre currently serving: _________

उपकेंद्र का नाम जिसे इसे समय सेवाएं प्रदान कर रही हैं:

Number of years in government service (in years): ____

सरकारी सेवाओं में सालों की संख्‍या (सालों में)

Number of years of service completed in the current Subcentre:

वर्तमान उपकेंद्र में पूरे किये गए सेवा के सालों की संख्‍या:

**HRP Pregnancy situation in your area**

आपके क्षेत्र में एचआरपी गर्भावस्‍था की स्थिति

Average number of ANCs at this Subcentre, per month:

इस उपकेंद्र पर हर महीने एएनसी की औसत संख्‍या:

Average number of pregnancies that are high-risk pregnancies out of all pregnancies in your subcentre, per month:_

आपके उपकेंद्र में प्रति माह सभी गर्भावस्‍थाओं में से उच्च जोखिम वाली गर्भावस्‍थाओं की औसत संख्या:_

Of the total high-risk pregnancies, how many are referred to designated facilities or specialists during ANC/delivery, per month: _________

**कुल उच्च जोखिम वाली गर्भावस्थाओं में से, प्रति माह एएनसी/प्रसव के दौरान कितनी को नामित सुविधाओं या स्‍पेशलिस्‍ट के पास भेजा जाता है:**

**Scope of work**

**कार्य क्षेत्र**

Do you think that you can play an important role in **diagnosing & manging high risk pregnancy conditions** during the antenatal period?

क्‍या आपको लगता है कि आप प्रसवपूर्व अवधि के दौरान **उच्‍च जोखिम वाली गर्भावस्‍था** की समस्‍याओं को डायग्‍नोस करने और प्रबंधित करने में एक महत्‍वपूर्ण भूमिका निभा सकती हैं?

1. To a great extent

काफी हद तक

1. To some extent

कुछ हद तक

1. Not at all

बिल्‍कुल भी नहीं

1. Not sure
2. पक्‍का नहीं

Do you think it is the responsibility of the ANM to take **extensive history** of all pregnant women when you register?

क्या आपको लगता है कि पंजीकरण करते समय सभी गर्भवती महिलाओं से उनका विस्तृत इतिहास लेना एएनएम की जिम्मेदारी है?

1. To a great extent

काफी हद तक

1. To some extent

कुछ हद तक

1. Not at all

बिल्‍कुल भी नहीं

1. Not sure

पक्‍का नहीं

Do you think it’s your responsibility **to provide counselling** including nutrition advice to the woman, about how to manage pregnancy, precautions to take as per diagnosed health condition, ensure she is aware about the hospital visits, why to visit a MO or a specialist if needed, if there are health concerns?

क्या आपको लगता है कि महिला को पोषण संबंधी सुझाव सहित इस पर परामर्श प्रदान करना आपकी जिम्‍मेदारी है कि, गर्भावस्था का प्रबंधन कैसे करें, डायग्‍नोस की गई स्वास्थ्य समस्‍या के अनुसार क्या सावधानियां बरतनी हैं, यह सुनिश्चित करना कि उसे हॉस्पिटल विजिट के बारे में पता है, यदि स्वास्थ्य संबंधी चिंताएं हैं तो विशेषज्ञ के पास जाने की आवश्यकता क्यों है, आदि है?

1. To a great extent

काफी हद तक

1. To some extent

कुछ हद तक

1. Not at all

बिल्‍कुल भी नहीं

1. Not sure
2. पक्‍का नहीं

Do you think it is your responsibility **to do early diagnosis of** high-risk condition during ANC registration or during following ANC visits?

क्या आपको लगता है कि क्‍या प्रारंभिक एएनसी पंजीकरण के दौरान या उसके बाद एएनसी विजिट के दौरान यह डायग्‍नोसक आपकी जिम्‍मेदारी है कि क्‍या महिला में कोई उच्च जोखिम वाली समस्‍या है?

1. To a great extent

काफी हद तक

1. To some extent

कुछ हद तक

1. Not at all

बिल्‍कुल भी नहीं

1. Not sure

पक्‍का नहीं

*Choose the THREE top most High risk conditions, higher in terms of number of cases (in order of higher to lower) that complicates pregnancy that you commonly see in your area:*

*अपने क्षेत्र में आमतौर पर देखी जाने वाली तीन सबसे उच्च जोखिम वाली समस्‍याओं को चुनें, जो केसेज की संख्या के मामले में अधिक हैं (उच्च से निम्न के क्रम में) जो गर्भावस्था को जटिल बनाती हैं:*

Rank 1.

रैंक 1

Rank 2:

रैंक 2

Rank 3.

रैंक 3

**According to you assign whether the below mentioned conditions is perceived by you as a high-risk pregnancy condition or not? If you are not sure and can’t decide, please select not sure.**

अपने अनुसार असाइन करें कि क्‍या नीचे दी गई समस्‍याएं आपके द्वारा उच्च जोखिम वाली गर्भावस्था संबंधी समस्‍याएं मानी जाती हैं या नहीं? यदि आपको पक्‍का नहीं पता है और निर्णय नहीं ले पा रहे हैं, तो कृपया पक्‍का नहीं चुनें।

| **S.No** | **Condition**  **समस्‍याएं** | 1. **Yes, HR**   **हां, एचआर**   1. **Not HR**   **एचआर नहीं**   1. **Not sure**   **पक्‍का नहीं** |
| --- | --- | --- |
| **1** | 17 yr. old primigravida, 24 weeks POG (Period of Gestation)  **17 वर्षीय प्राइमिग्रेविडा, 24 सप्ताह पीओजी (गर्भावस्था की अवधि)** |  |
| **2** | G7P6L6, 37 weeks POG with labour pains  G7P6L6**, प्रसव पीड़ा के साथ 37 सप्ताह का पीओजी** |  |
| **3** | Primigravida 32 wks. POG with BP 150/90 mmHg  **प्राइमिग्रेविडा 32 सप्ताह पीओजी के साथ बीपी 150/90 mmHg** |  |
| **4** | G2P1L1, 10 wks. POG with TSH >6 uIU/ml  **G2P1L1, 10 सप्ताह। TSH >6 uIU/ml के साथ पीओजी** |  |
| **5** | G3P2L2 20 wks. POG with Hb 8.8 mg/dl  **G2P1L1, 20 सप्ताह।** हीमोग्‍लोबिन 8.8 mg/dl **के साथ पीओजी** |  |
| **6** | G2P1L1, 38 wks. POG with H/O previous delivery by LSCS  G2P1L1, 38 **सप्ताह।** एलएससीएस द्वारा एच/ओ पिछली डिलीवरी के साथ पीओजी |  |
| **7** | Primigravida 28 weeks with painless bleeding P/V  **दर्द रहित रक्तस्राव पी/वी के साथ प्राइमिग्रेविडा 28 सप्ताह** |  |
| **8** | Primigravida 34 weeks with pain abdomen with bleeding P/V  प्राइमिग्रेविडा 34 सप्ताह पेट दर्द के साथ रक्तस्राव पी/वी |  |
| **9** | G2P1L1, 24 wks. POG with watery discharge P/V  G2P1L1, 24 सप्‍ताह। पानी जैसे डिस्चार्ज पी/वी के साथ पीओजी |  |
| **10** | Primigravida 32 weeks with fever with chills  प्राइमिग्रेविडा 32 सप्ताह बुखार के साथ ठंड लग रही है |  |
| **11** | G7P6L6, 37 weeks POG with Hb 7 gm/dl  G7P6L6, 37 सप्‍ताह, पीओजी के साथ हीमोग्‍लोबिन 7 gm/dl |  |
| **12** | G3P2L2 with 24 weeks POG with palpitations and breathlessness  G3P2L2 24 सप्ताह, घबराहट और सांस फूलने के साथ पीओजी |  |
| **13** | Primigravida 34 weeks with seizures and headache  दौरे पड़ने और सिरदर्द के साथ प्राइमिग्रेविडा 34 सप्ताह |  |
| **14** | 38 yr. Primigravida 32 weeks POG with Hb 12 mg/dl  **38 वर्ष, प्राइमिग्रेविडा, 32 सप्ताह, Hb 12 mg/dl के साथ के साथ पीओजी** |  |
| **15** | G2P1L1, 24 wks. POG with jaundice  G2P1L1, 24 सप्‍ताह, पीलिया के साथ पीओजी |  |

**Kindly suggest where do currently treat or refer for the following conditions?**

कृपया सुझाव दें कि मौजूदा समय में आप निम्नलिखित समस्‍याओं के लिए कहां उपचार किया या रेफर किया जाता है?

| **S.No** | **Condition** | 1. **Treat myself**   **खुद उपचार करती हैं**   1. **Treat it at PHC**   **इसका उपचार पीएचसी पर करवाती हैं**   1. **I will stabilize & refer to higher facility** 2. **मैं स्थिर करूंगी और उच्च सुविधा को रेफर करूंगी** |
| --- | --- | --- |
| **1** | Primigravida 20 weeks pregnancy with Hb < 10 gm% |  |
| **2** | Gravida 2 with one living issue, 34 weeks POG with Hb < 7 |  |
| **3** | Gravida 2 with one live issue term pregnancy with h/o previous delivery by LSCS |  |
| **4** | Primigravida 32 weeks POG with BP > 150/90 |  |
| **5** | Gravida 3, 24 weeks with OGTT 260 mg/dl |  |
| **6** | Primigravida 36weeks POG with BP > 140/90, with headache |  |
| **7** | Gravida 3, 10 weeks POG with pain lower abdomen and spotting |  |
| **8** | Gravida 3, 32 weeks POG with painless bleeding |  |
| **9** | Gravida 4, with 3 living issues with 34 weeks POG with Prev 2 LSCS with pain and bleeding |  |
| **10** | Gravida 2 with one live issue term pregnancy with h/o epilepsy |  |
| **11** | Primigravida 20 weeks POG with breathlessness and palpitations |  |
| **12** | Gravida 3, 24 weeks with jaundice |  |

**Kindly choose the medical tests you currently provide at your subcentre to pregnant women?**

कृपया उन मेडिकल टेस्‍ट्स को चुनें जो आप वर्तमान में अपने उपकेंद्र पर गर्भवती महिलाओं को प्रदान करते हैं?

BG Yes No

बीजी हां नहीं

Haemoglobin Yes No

होमोग्‍लोबिन हां नहीं

Urine routine & microscopy Yes No

यूरीन रूटी और माइक्रोस्‍कोपिक हां नहीं

Hepatitis Yes No

हेपेटाइटिस हां नहीं

HIV Yes No

एचआईवी हां नहीं

RPR/VDRL Yes No

आरपीआर/वीडीआरएल हां नहीं

Blood grouping of husband Yes No

पति की ब्‍लड ग्रुपिंग हां नहीं

Glucose OGTT Yes No

ग्‍लूकोज ओजीटीटी हां नहीं

Urine culture Yes No

यूरीन कल्‍चर हां नहीं

Urine-protein/ Sugar Yes No

यूरीन-प्रोटीन/शुगर हां नहीं

Urobilinogen-dipstic Yes No

यूरोबिलिनोजेन-डिपस्टिक हां नहीं

RDT Rapid diagnostic test-**malaria** and or Microscopy-Thick and thin smear Yes No

आरडीटी रैपिड डायग्नोस्टिक टेस्ट-मलेरिया और/या माइक्रोस्कोपी-थिक और थिन स्मीयर हां नहीं

**Share your perception on the following statements.**

**निम्‍नलिखित वाक्‍यों पर अपनी राय को साझा करें।**

Do you think you have enough training, knowledge and skills to manage **ALL** high-risk pregnancy conditions?

मुझे सभी उच्च जोखिम वाली गर्भावस्था संबंधी समस्‍याओं का उपचार करने के लिए पर्याप्त ट्रेनिंग, ज्ञान और कौशल प्राप्त है

1. Agree

सहमत

1. Neither agree or nor disagree (not sure)

ना तो सहमत और न ही असहमत (पक्‍का नहीं)

1. Disagree
2. असहमत

Do you think all women with any high-risk conditions should be referred to specialist right away during antenatal period

मेरा मानना ​​है कि किसी भी उच्‍च जोखिम वाली सभी महिलाओं को प्रसवपूर्व अवधि के दौरान तुरंत स्‍पेशलिस्‍ट के पास भेजा जाना चाहिए

1. Agree

सहमत

1. Neither agree or nor disagree

ना तो सहमत और न ही असहमत

1. Disagree

असहमत

**Current Knowledge platforms**

**मौजूदा नॉलेज प्‍लेटफॉर्म**

What do you do if you have doubts related to managing a pregnancy case? [Tick all applicable answers]

यदि गर्भावस्‍था के मामले को प्रबंधित करने के संबंध में आपको कोई संदेह है तो आप क्‍या करते हैं?[लागू होने वाले सभी जवाबो को टिक करें]

1. Reach out to doctor

डॉक्‍टर्स से संपर्क करती हैं

1. Reach out to senior ANM

सीनियर एएनएम से सपंर्क करती हैं

1. Reach out to peer ANMs
2. साथी एएनएम से संपर्क करती हैं
3. Browse the internet

इंटरनेट ब्राउज करती हैं

1. Refer the guidelines material

गाइडलाइन मटीरियल/किताबों को देखती हैं

**Access and use of Technology**

**टेक्‍नोलॉजी तक पहुंच और इस्‍तेमाल**

How comfortable are you to use personal mobile phone for official training programmes?

आधिकारिक ट्रेनिंग कार्यक्रमों के लिए आप अपना निजी मोबाइल फोन का इस्‍तेमाल करने में कितना सहज होते हैं?

1. Very comfortable

बहुत सहज

1. Not so comfortable

बहुत सहज नहीं

1. Uncomfortable

असहज

Which one do you use more frequently?

आप किसे ज्‍यादा अक्‍सर इस्‍तेमाल करते हैं?

1. SMS

एसएमएस

1. WhatsApp

वॉट्सऐप

1. Other Chatting apps (telegram, Signal)

अन्‍य चैटिंग ऐप्‍स (टेलीग्राम, सिग्‍नल)

1. Both SMS and WhatsApp

एसएमएस और वॉट्सऐप दोनों

How would you assess the internet connectivity when you are at the subcentre?

जब आप उपकेंद्र में हों तो आप इंटरनेट कनेक्टिविटी का मूल्‍यांकन कैसे करेंगे?

- - 1. Yes, always good connectivity

हां, हमेशा अच्‍छी कनेक्‍टीविटी

- - 1. No, poor connectivity
    2. नहीं, खराब कनेक्टिविटी
    3. Sometime good and other times poor connectivity

कभी-कभी अच्‍छी और अन्‍य समय खराब कनेक्टिविटी

How would you assess the internet connectivity when you are in the field/Home visits?

जब आप फील्ड/होम विजिट पर होती हैं तो आप इंटरनेट कनेक्टिविटी का कैसे मूल्‍यांकन करेंगी?

1. Yes, always good connectivity

हां, हमेशा अच्‍छी कनेक्‍टीविटी

1. No, poor connectivity
   नहीं, खराब कनेक्टिविटी
2. Sometime good and other times poor connectivity

कभी-कभी अच्‍छी और अन्‍य समय खराब कनेक्टिविटी

How comfortable are you with using RCH portal

आप आरसीएच पोर्टल का इस्‍तेमाल करने में कितनी सहज हैं

1. Very comfortable

बहुत सहज

1. Not so comfortable

बहुत सहज नहीं

1. Very uncomfortable

बहुत असहज

Do you think using tablets for gathering information from pregnant women is useful?

क्या आपको लगता है कि गर्भवती महिलाओं से जानकारी जुटाने के लिए टैबलेट का इस्‍तेमाल उपयोगी है?

1. Yes, very useful

हां, बहुत उपयोगी

1. Not sure, can’t say

पक्‍का नहीं, कह नहीं सकती

1. No, not useful

नहीं, उपयोगी नहीं

What problems do you expect to face while using tab in the field/community for learning and data collection? (tick all applicable)

सीखने और डेटा कलेक्‍शन के लिए फील्‍ड/समुदाय में टैब का इस्‍तेमाल करते समय आपको किन समस्याओं का सामना करना पड़ सकता है? (लागू होने वाले सभी को मार्क करें)

1. Tab screen sometimes doesn’t work

कभी-कभी टैब की स्‍क्रीन काम नहीं करती है

1. Tab works very slow

टैब बहुत धीमें काम करता है

1. Internet connection in tab is difficult

टैब में इंटरनेट कनेक्टिविटी कठिन है

1. The battery charge of the tab is poor

टैब की बैट्री चार्जिंग खराब है

1. It may take more time to do all the entry in the field itself

फील्‍ड में ही सारी एंट्री करने में ज्‍यादा समय लग सकता है

1. It may break
2. यह टूट सकता है
3. Difficult to use during rains
4. बारिश के दौरान इस्‍तेमाल करना कठिन है
5. Sometimes during power failure, it’s difficult to charge.

बिजली ना आने पर, इसे चार्ज करना मुश्किल होता है।

**Checklist of 15 questions for each HRP**

**प्रत्‍येक एचआरपी के लिए 15 प्रश्‍नों की चेकलिस्‍ट**

**Anemia**

**एनीमिया**

1)What is anaemia?

एनीमिया क्‍या है?

a)Deficiency of red blood cells or their oxygen-carrying capacity

लाल रक्त कोशिकाओं या उनकी ऑक्सीजन ले जाने की क्षमता की कमी

b)Deficiency of proteins in the body

शरीर में प्रोटीन की कमी

c)Deficiency of fats in the body

शरीर में फैट की कमी

d)None of the above

इनमें से कोई नहीं

e)Don't Know

पता नहीं

2) Anaemia in pregnancy is defined as

गर्भावस्था में एनीमिया को इस प्रकार परिभाषित किया गया है

a)Hb levels < 11gm/dl

एचबी स्तर < 11 gm/dl

b)Hb levels < 12gm/dl

एचबी स्तर < 12 gm/dl

c)Hb levels < 13 gm/dl

एचबी स्तर < 13 gm/dl

d)None

कोई नहीं

3) Current method of haemoglobin estimation in the field is using:

फील्‍ड में हीमोग्‍लोबिन का आकलन के लिए इस्‍तेमाल किया जाने वाला मौजूदा तरीका है:

a)Cyanmethemoglobin method

सायनमेथेमोग्लोबिन विधि

b)Alkaline- hematin method

एल्‍केलाइन-हेमाटीन विधि

c)Digital haemoglobinometer

डिजिटल हीमोग्लोबिनोमीटर d)Don't Know

पता नहीं

4) Correct classification of anaemia in pregnancy is:

गर्भावस्था में एनीमिया का सही वर्गीकरण इस प्रकार हैः

a)Mild: Hb 10-10.9 g/dl, Moderate: Hb 7-9.9g/dl, Severe: <7

हल्‍का: एचबी 10-10.9 g/dl, मध्‍यम: एचबी 7-9.9g/dl, गंभीर: <7

b)Mild: Hb 11-11.9 g/dl, Moderate: Hb 8- 10g/dl, Severe: <8

हल्‍का: एचबी 11-11.9 g/dl, मध्‍यम: एचबी 8- 10g /dl, गंभीर: <8

c)Mild: Hb 11-12.9 g/dl, Moderate: Hb 8- 10.9g/dl, Severe: <8

हल्‍का: एचबी 11-11.9 g/dl, मध्‍यम: एचबी 8- 10g /dl, गंभीर: <8 =2

d)None

कोई नहीं e)Don't Know

पता नहीं

5)What are the ways of preventing anaemia in pregnancy?

गर्भावस्था में एनीमिया को रोकने के क्या तरीके हैं?

a)Deworming

कृमिनाशक

b)Iron and Folic acid supplementation

आयरन और फोलिक एसिड सप्‍लीमेंटेशन

c)Increase intake of iron-rich food and vitamin C rich-foods

आयरन युक्त भोजन और विटामिन C युक्त भोजन का सेवन बढ़ाना

d)All of the above

उपरोक्‍त सभी

6) What is the recommended dose of iron and folic acid tablets in pregnant women with 8grms/dl haemoglobin in the 2nd trimester?

दूसरे ट्राइमेस्टर में 8grms/dl हीमोग्‍लोबिन वाली गर्भवती महिला के लिए आयरन और फोलिक एसिड की गोलियों की सुझाई गई डोज क्या है?

a)Two tablets of iron and folic acid (100 mg elemental iron and 500 mcg folic acid) daily for 6 months

आयरन और फोलिक एसिड की दो टैबलेट्स (100 mg एलीमेंट्स आयरन और 500 mcg फोलिक एसिड) 6 महीने तक प्रतिदिन

b)One tablet of iron and folic acid (100 mg elemental iron and 500 mcg folic acid) daily for 6 months

आयरन और फोलिक एसिड की एक टैबलेट्स (100 mg एलीमेंट्स आयरन और 500 mcg फोलिक एसिड) 6 महीने तक प्रतिदिन

c)Only iron tablet

केवल आयरन की टैबलेट

d)None

कोई नहीं e)Don't Know

पता नहीं

7) Can anaemia in pregnant women affect the baby?

क्या गर्भवती महिला में एनीमिया बच्चे को प्रभावित कर सकता है?

a)Yes, it can cause preterm, low birth weight babies

हाँ, इससे समय से अपरिपक्व, कम वजन वाले बच्चे पैदा हो सकते हैं

b)No, it can only lead to symptoms in the mother

नहीं, इससे केवल माँ में ही लक्षण उत्पन्न हो सकते हैं

c)No, it can only lead to complications during delivery

नहीं, इससे केवल प्रसव के दौरान ही जटिलताएं उत्पन्न हो सकती हैं

d)None of the above

उपरोक्‍त में से कोई नहीं e)Don't Know

पता नहीं

8)What are the common symptoms with which a patient with anemia may present?

एनीमिया के रोगी में कौन से सामान्य लक्षण दिखाई दे सकते हैं?

a)Fatigue, palpitations, breathlessness, giddiness

थकान, घबराहट, सांस फूलना, चक्कर आना

b)Cough, blood in sputum, fever

खांसी, बलगम में खून, बुखार

c)Nausea, vomiting, diarrhoea

मतली, उल्टी, दस्त

d)Headache, loss of vision, vomiting

सिरदर्द, नज़र कमजोर होना, उल्टी

e) Don't Know

पता नहीं

9)What are the signs of anaemia on clinical examination?

चिकित्सीय जांच में एनीमिया के लक्षण क्या हैं?

a)Clubbing

क्लबिंग

b)Pallor

पीलापन

c)Pedal oedema

पेडल इडीम

d)All of the above

उपरोक्‍त सभी

10)Estimation of haemoglobin levels in pregnancy should be done:

गर्भावस्था में हीमोग्लोबिन के स्तर का आकलन किया जाना चाहिए:

a)At 12, 24, and 36 weeks

12, 24 और 36 सप्ताह पर

b)In the first and last visit

पहली और आखिरी विजिट में

c)At all ANC visits

सभी एएनसी विजिट में

d)None

कोई नहीं

e)Don't Know

पता नहीं

11) Out of the given options, choose the correct nutritional advice for a pregnant woman with anaemia:

दिए गए विकल्पों में से, सही पोषण संबंधी सलाह चुनें जो गर्भावस्‍था में एनीमिया से पीड़ित महिला को दी जानी चाहिए:

a)Low salt diet

कम नमक वाला आहार

b)Low carbohydrate, high fat diet

कम कार्बोहाइड्रेट, उच्च फैट वाला आहार

c)Diet rich in proteins, iron, vitamin C, and other micronutrients

प्रोटीन, आयरन, विटामिन सी और अन्य माइक्रोन्‍यूट्रीएंट्स से भरपूर आहार

d)Low sugar diet

कम शुगर वाला आहार e)Don't Know

पता नहीं

12) 26 yr., primigravida at 22 weeks POG was tested to have a Hb of 10g/dl, mark the correct statement about her management plan:

26 वर्षीय प्राइमिग्रेविडा का 22 सप्ताह के पीओजी का टेस्‍ट किया गया जिसमें Hb 10g/dl पाया गया, उसके प्रबंधन योजना के बारे में सही वाक्‍य को मार्क करें:

1. She is a low risk so can be given usual care

उसे कम जोखिम है इसलिए उसे सामान्य देखभाल दी जा सकती है

1. She has mild anaemia, needs to be managed as a high-risk pregnancy

उसे हल्का एनीमिया है, उसका उच्च जोखिम वाली गर्भावस्था के रूप में उपचार किया जाना चाहिए

1. She has severe anaemia, needs to be referred to a tertiary care center

उसे गंभीर एनीमिया है, उसे टर्शर केयर सेंटर में रेफर करने की आवश्यकता है

d)None कोई नहीं

13) 26 yr., primigravida received recommended dose of iron and folic acid tablets for mild anemia for one month. On repeat testing a month later, no improvement was seen in her Hb levels. What will you do next?

26 वर्षीय प्राइमिग्रेविडा को हल्के एनीमिया के लिए एक महीने तक आयरन और फोलिक एसिड की गोलियों की सिफारिश की गई डोज दी गई। एक महीने बाद दोबारा जांच करने पर, उसके हीमोग्‍लोबिना के स्तर में कोई सुधार नहीं देखा गया। अब आप क्या करेंगी?

a)Check for compliance

जांच करेंगे कि क्‍या डोज का पालन किया गया

b)If compliance is good, refer to FRU/DH for further workup

यदि ठीक तरह से पालन किया गया, तो आगे की कार्यवाही करने के लिए एफआरयू/डीएच को रेफ करेंगे

c)Give her injectable iron

उसे आयरन का इंजेक्‍शन देंगे

d)Both a & b

a और b दोनों

14)What precautions will you ask the pregnant woman to take while taking iron tablets:

आयरन की टैबलेट्स लेते समय आप गर्भवती महिला को क्या सावधानियां बरतने के लिए कहेंगे:

a)Take Iron tablets for 1 hr. after food

आयरन की टैबलेट खाने के 1 घंटे के बाद खाएं

b)Keep a gap of 2hrs between iron and calcium tablets

आयरन और कैल्शियम की टैबलेट्स के बीच 2 घंटे का अंतर रखें

c)Do not take iron tablets with milk/tea/coffee

आयरन की टैबलेट्स दूध/चाय/कॉफी के साथ न लें

d)Taking iron with lemon water may increase its absorption

नींबू पानी के साथ आयरन लेने से इसका अवशोषण बढ़ सकता है

e)All of the above

उपरोक्‍त सभी

15) Which of the following cases should be referred to a higher center:

इनसे से कौन से केसेस को हायर सेंटर को भेजा जाना चाहिए:

a)All pregnant women with Hb < 7 - 9.9 g/dl

सभी गर्भवती महिलाएं जिनका एचबी < 7-9.9 g/dl =1 है

b)Any pregnant woman with pallor and breathlessness (RR >24/min) and or tachycardia (PR>100 bpm)

कोई भी गर्भवती महिला जिसका शरीर पीला पड़ा हो और सांस फूल रही हो (RR >24/मिनट) और या तीव्र हृदयगति (पीआर>100 bpm) =2

c)All pregnant women who develop vomiting/severe nausea/diarrhea/severe constipation with IFA

सभी गर्भवती महिलाएं जिनमें आईएफबी से उल्टी/गंभीर मतली/दस्त/गंभीर कब्ज की समस्या होती है

d)All of the above

उपरोक्‍त सभी

e)Don't Know

पता नहीं

**Antepartum Haemorrhage (APH)**

**प्रसवपूर्व रक्तस्राव (एपीएच) :**

1. Antepartum haemorrhage (APH) is defined as

प्रसवपूर्व रक्तस्राव (एचपीएच) को इस प्रकार परिभाषित किया जाता है

a)bleeding from the genital tract, occurring after 20 weeks of pregnancy and prior to the birth of the baby

गर्भावस्था के 20 सप्ताह के बाद और बच्चे के जन्म से पहले जननांग मार्ग से या उसके अन्दर रक्तस्राव होना

b)bleeding from the genital tract, occurring from 24 weeks of pregnancy and prior to the birth of the baby

गर्भावस्था के 24सप्ताह के बाद और बच्चे के जन्म से पहले जननांग मार्ग से या उसके अन्दर रक्तस्राव होना

c)bleeding from the genital tract, occurring before 20 weeks of pregnancy and prior to the birth of the baby

गर्भावस्था के 20 सप्ताह के पहले और बच्चे के जन्म से पहले जननांग मार्ग से या उसके अन्दर रक्तस्राव होना, =3

d)None of the above

उपरोक्त में से कोई नहीं e)Don't Know

पता नहीं

1. Which of the following is true for APH?

इनमें से कौन सा एपीएच के लिए सही है?

a)A little bleeding after 20 weeks is normal

20 सप्ताह के बाद थोड़ा रक्तस्राव होना सामान्य है

b)It is always associated with pain abdomen

यह हमेशा पेट दर्द से जुड़ा होता है

c)It is always painless

यह हमेशा दर्द रहित होता है

d)None of the above

उपरोक्त में से कोई नहीं

e)Don't Know

पता नहीं

1. APH is a high-risk condition as:

एपीएच एक उच्‍च जोखिम वाली समस्‍या हो सकती है:

a)Bleeding can affect both mother and the baby

रक्‍तस्राव मां और बच्‍चे दोनों को प्रभावित कर सकता है

b)Bleeding can affect only the mother

रक्‍तस्राव केवल मां को प्रभावित कर सकता है

c)Bleeding can affect only the baby

रक्‍तस्राव केवल बच्‍चे को प्रभावित कर सकता है

d)None of the above

उपरोक्‍त में से कोई नहीं

1. Causes of APH are:

एपीएच के कारण हैं:

a)Placenta previa

प्लेसेंटा प्रीविया

b)Abruptio placentae

एब्रुपियो प्लेसेंटा

c)Local causes

स्थानीय कारण

d)All of the above

उपरोक्‍त सभी

e)Don't Know

पता नहीं

1. A pregnant woman with low lying placenta

एक गर्भवती महिला जिसकी नाल नीचे की ओर झुकी हुई है

a)Can be delivered at a PHC

पीएचसी पर डिलीवरी की जा सकती है

b)Can be delivered at a subcentre

उपकेंद्र पर डिलीवरी की जा सकती है

c)Should be delivered at a DH/tertiary care centre where a specialist is available

डीएच/टर्शरी केयर सेटर में डिलीवरी कराई जानी चाहिए जहां स्‍पेशलिस्‍ट उपलब्ध हो

d)Not sure

पक्‍का नहीं

1. A 24 yr old primigravida at 32 weeks POG presents to you with c/o painless spotting P/V. On examination her pulse and BP are normal, fundal height corresponds to 32 weeks with normal fetal heart sounds. Her pad is minimally soaked. What will you do next?

32 सप्ताह के पीओजी में 24 वर्षीय प्राइमिग्रेविडा आपके पास आती है जिसे दर्द रहित स्पॉटिंग P/V है। जांच करने पर उसकी पल्‍स और बीपी सामान्य है, फंडल की ऊंचाई 32 सप्ताह के अनुरूप है और भ्रूण की हार्ड का साउंड सामान्य है। उसका पैड बहुत भीगा हुआ है। आप आगे क्या करेंगी?

a)Do a P/S & P/V examination

पी/एस और पी/वी परीक्षण करेंगी

b)Send her home after counseling

परामर्श के बाद उसे घर भेज देंगी

c)Check her Hb levels and refer to MO

उसके हीमोग्‍लोबिन के स्‍तर की जांच करेंगी और एमओ को रेफर करेंगी

d)None of the above

उपरोक्‍त में से कोई नहीं

1. Don't Know

पता नहीं

1. A 28yr old G3P2L2 with 36 weeks POG is brought to you in a semi-conscious state with pulse rate > 100/ min, BP 90/60 mmHg with bleeding P/V for 30 mins. Her clothes are soaked with blood. How will you manage?

28 वर्ष की एक G3P2L2 को 36 सप्ताह के पीओजी के साथ आधी बेहोशी की हालत में आपके पास लाया गया है, जिसकी पल्‍स रेट > 100/मिनट, बीपी 90/60 mmHg है तथा 30 मिनट तक P/V से रक्तस्राव हो रहा है। उसके कपड़े खून से भीगे हुए हैं। आप मामले को कैसे संभालेंगे?

a)Put a large bore I.V cannula and start fluids

एक लार्ज बोर I.V कैनुला डालेंगे और फ्लूइड शुरू करेंगी

b)Take samples for blood grouping and cross matching

ब्लड ग्रुपिंग और क्रॉस मैचिंग के लिए सैंपल लेंगी

c)Transport the patient yourself to a tertiary care center

रोगी को स्वयं टर्शरी केयर सेंटर ले जाएंगी

d)All of the above

उपरोक्‍त में से कोई नहीं

1. Don't Know

पता नहीं

8) How will you counsel a pregnant woman with placenta previa on USG? "

आप यूएसजी पर प्लेसेंटा प्रीविया से पीड़ित गर्भवती महिला को कैसे परामर्श देंगी?

a)Tell her about the warning signs

उसे चेतावनी संकेतों के बारे मे बताएंगी

b)Tell her about the need to register herself at a tertiary care center

उसे खुद का टर्शर केयर सेंटर में पंजीकरण कराने की आवश्यकता के बारे में बताएंगी

c)Both A and B

ए और बी दोनों

d)None of the above

उपरोक्‍त में से कोई नहीं

e)Don't Know

पता नहीं

9) While transferring a pregnant woman of APH to a tertiary care hospital, the following should be kept in mind:

एपीएच के रोगी को टर्शरी केयर हॉस्पिटल में ट्रांसफर करते समय, इन बातों को ध्यान में रखा जाना चाहिए:

a)Send a skilled provider with the woman to ensure an open airway, to deliver first aid if she goes into shock

महिला के साथ एक कुशल प्रदाता को भेजें ताकि वायुमार्ग खुला रहे, ताकि यदि वह सदमे में चली जाए तो प्राथमिक उपचार दिया जा सके

b)Elevate legs to improve blood supply to vital organs

महत्वपूर्ण अंगों में रक्त की आपूर्ति में सुधार करने के लिए पैरों को ऊपर उठाएं =2

c)Avoid hypothermia- keep her warm

हाइपोथर्मिया से बचाएं- उसे गर्म रखें

d)All of the above

उपरोक्‍त में से कोई नहीं

e)Don't Know

पता नहीं

10) Following facilities should be available at any center to ensure safe delivery of a woman with APH:

एपीएच से पीड़ित महिला की सुरक्षित डिलीवरी सुनिश्चित करने के लिए किसी भी केंद्र पर निम्नलिखित सुविधाएं उपलब्ध होनी चाहिए:

a)Fully operational blood bank

पूरी तरह से चालू ब्‍लड बैंक

b)24 X 7, availability of senior obstetrician, pediatrician, and anesthetist

24 x 7, सीनियर प्रसूति रोग विशेषज्ञ, बाल रोग विशेषज्ञ एवं एनेस्थेटिस्ट की उपलब्धता

c)Fully operational laboratory

पूरी तरह से चालू लेबोरेटरी

d)All of the above

उपरोक्‍त सभी

11) While referring a patient to a higher centre in view of APH what all points should she and her family is counselled about:

एपीएच को ध्‍यान में रखते हुए किसी रोगी को हयर सेंटर में रेफर करते समय उसे और उसके परिवार को किन-किन बातों पर परामर्श दिया जाना चाहिए:

a)High risk to mother and foetus

मां और भ्रूण के लिए उच्‍च जोखिम

b)Need for shifting immediately

तुरंत शिफ्ट करने की आवश्‍यकता

c)Blood donation/transfusion

ब्‍लड डोनेशन/ट्रांसफ्यूज़न

d)ALL of the above

उपरोक्‍त सभी

e)Don't Know

पता नहीं

12) Which of the following is true?

इनमें से कौन सा सही है?

a)APH is not an emergency if patients’ vitals are stable

यदि मरीज के वाइटल्‍स स्थिर है तो एपीएच आपातकालीन स्थिति नहीं है।

b)Pregnant women with abruptio placentae can be delivered at PHC

एब्रुपियो प्लेसेंटा वाली गर्भवती महिलाओं की डिलीवरी पीएचसी में कराई जा सकती है।

c)While referring Pregnant women of APH all the details of her clinical findings and treatment received should be mentioned on the referral slip.

एपीएच की गर्भवती महिलाओं को रेफर करते समय उनके क्लिनिक ​​निष्कर्षों और दिये गए उपचार का पूरा विवरण रेफरल स्लिप पर लिखा जाना चाहिए।

d)Don't Know

पता नहीं

13) Which of the following pregnant women is at high risk for APH?

इनमें से किस गर्भवती महिला को एपीएच का उच्च जोखिम होता है?

a)Pregnant women with h/o APH in a previous pregnancy

पिछली गर्भावस्था में एच/ओ एपीएच से पीड़ित गर्भवती महिलाएं

b)Preeclampsia

प्रीक्लेम्पसिया

c)Pregnant women with previous delivery by LSCS

पिछली डिलीवरी एलएससीएस द्वारा करा चुकी गर्भवती महिलाएं

d)All of the above

उपरोक्‍त सभी

e)Don't Know

पता नहीं

14) Pregnant women with h/o last delivery by LSCS are at increased risk of

एलएससीएस द्वारा पिछली डिलीवरी वाली गर्भवती महिलाओ को इसका खतरा बढ़ जाता है

a)Placenta previa

प्लेसेंटा प्रिविया

b)Uterine rupture

गर्भाशय का टूटना

c)Both (a) and (b)

a और b दोनों

d)None

कोई नहीं

15) Primi woman at 28 weeks POG comes with h/o BPV (bleeding per vagina) for 30 mins. Which of the following will you ask her?

28 सप्ताह के पीओजी वाली में प्राइमी महिला 30 मिनट तक h/o बीपीवी (योनि से रक्तस्राव) के साथ आती है। आप उससे निम्नलिखित में से क्या पूछेंगी?

a)Any documented USG

कोई डाक्‍यूमेंटेड यूएसजी

b)h/o IFA intake

एच/ओ आईएफए का सेवन

c)h/o UTI

एच/ओ यूटीआई

d)None

कोई नहीं

1. Don't Know

पता नहीं

**Pregnancy Induced Hypertension (PIH)**

**गर्भावस्था से प्रेरित हाइपरटेंशन (पीआईएच)**

1) What is hypertension?

हाइपरटेंशन क्‍या है?

a)A condition in which the force of the blood against the artery walls is too high.

एक ऐसी स्थिति जिसमें धमनी की दीवारों पर ब्‍लड का दबाव बहुत अधिक होता है।

b)A condition in which the force of the blood against the artery walls is low.

एक ऐसी स्थिति जिसमें धमनी की दीवारों पर ब्‍लड का दबाव बहुत कम होता है।

c)A condition in which the blood pressure is raised.

एक ऐसी स्थिति जिसमें ब्‍लड प्रेशर बढ़ता है

d)Both a & c

ए और सी दोनों

e)None of the above

उपरोक्‍त में से कोई नहीं

2) Hypertension in pregnancy is defined as?

गर्भावस्था में हाइपरटेंशन को इस प्रकार परिभाषित किया जाता है?

a)Systolic BP >=140 mm of Hg; diastolic >=90 mm of Hg

सिस्‍टोलिक बीपी >=140 mm of Hg; डायस्टोलिक >=90 mm of Hg

b)Systolic BP <120 mm of Hg; diastolic <80 mm of Hg

सिस्‍टोलिक बीपी <120 mm of Hg; डायस्टोलिक <80 mm of Hg

c)Systolic BP >=90 mm of Hg; diastolic >=140mm of Hg

सिस्‍टोलिक बीपी >=90 mm of Hg; डायस्टोलिक >=140mm of

d)None of the above

उपरोक्‍त में से कोई नहीं

e)Don't Know

पता नहीं

3) Which of the following is a warning symptom of hypertension in pregnancy?

इनमें से कौन सा गर्भावस्था में हाइपरटेंशन की चेतावनी का लक्षण है?

1. Generalized oedema which is non-dependent

सामान्यीकृत एडिमा जो गैर-निर्भर है

1. Frequent urination

बार-बार पेशाब आना

1. Loss of appetite

भूख न लगना

d)All of the above

उपरोक्‍त सभी

e)None of the above

इनमें से कोई नहीं

4) Risk factors for preeclampsia are:

प्रीक्लेम्पसिया के जोखिम के कारक हैं:

a)High B.P in previous pregnancy

पिछली गर्भावस्था में हाई बीपी

b)Age >35 years

आयु 35 वर्ष से अधिक

c)First pregnancy

पहली गर्भावस्‍था

d)All of the above
 उपरोक्‍त सभी

5) What are the signs of hypertension on clinical examination during ANC visit?

एएनसी विजिट के दौरान क्‍लीनिकल परीक्षण में हाइपरटेंशन के लक्षण क्या हैं?

a)Excessive weight gain

अत्यधिक वजन बढ़ना

b)BP more than 140/90 mm of Hg

बीपी 140/90 mm of Hg से अधिक होना

c)Non-dependent pedal oedema

नॉन-डिपेंडेंट पेडल इडीम

d)All of the above

उपरोक्‍त सभी

e)Don't Know

पता नहीं

6) What is the next step after a pregnant woman is detected with hypertension during ANC visit?

एएनसी जांच के दौरान किसी गर्भवती महिला में हाइपरटेंशन का पता चलने पर अगला कदम क्या होता है?

a)Deworming

कृमिनाशक

b) counselling on diet change

आहार में बदलाव पर परामर्श

c)Refer the patient to MO or FRU

रोगी को एमओ या एफआरयू को रेफर करना

d)Start medications

दवा शुरू करना e)Don't Know

पता नहीं

7) What are the medications given to treat hypertension in pregnancy?

गर्भावस्था में हाइपरटेंशन का उपचार करने के लिए कौन सी दवाएं दी जाती हैं?

a)Iron and folic acid tablets

आयरन और फोलिक एसिड की टैबलेट्स

b)Cap. Nifedipine 10 mg

निफेडिपिन 10 mg का कैप्‍सूल

c)Tab. Labetalol 200 mg

लैबेटालोल 200 mg की टैबलेट

d)Both b & c

बी और सी दोनों

1. Can hypertension in pregnant women affect the baby?

क्या गर्भवती महिलाओं में हाइपरटेंशन से बच्‍चे पर असर पड़ सकता है?

a)Yes, it can cause decreased placental blood flow and preterm babies.

हां, इससे प्लेसेंटा में रक्त प्रवाह कम हो सकता है और बच्‍चे समय से पहले पैदा हो सकते हैं।

b)No, it can only lead to symptoms in the mother

नहीं, यह केवल माँ में लक्षण पैदा कर सकता है

c)No, it can only lead to complications during delivery

नहीं, इससे केवल डिलीवरी के दौरान ही समस्‍याएं उत्पन्न हो सकती हैं।

d)None of the above

उपरोक्‍त में से कोई नहीं

e)Don't Know

पता नहीं

1. What are the warning symptoms a patient of preeclampsia may present with?

प्रीक्लेम्पसिया के रोगी में कौन से चेतावनी के लक्षण दिखाई दे सकते हैं?

a)Fatigue, giddiness, pallor

थकान, चक्कर आना, पीलापन

b)Cough, blood in sputum, fever

खांसी, बलगम में खून, बुखार

c)Nausea, vomiting, diarrhea

मतली, उल्टी, दस्त

d)Headache, blurry vision, vomiting

सिरदर्द, धुंधला दिखाई देना, उल्टी

e)Don't Know

पता नहीं

1. Blood pressure levels in pregnancy should be checked:

गर्भावस्था में ब्‍लड प्रेशर के स्तर की जाँच की जानी चाहिए:

a)At 12, 24, and 36 weeks

12, 24 और 36 सप्ताह पर

b)In the first and last visit

पहली और आखिरी विजिट में

c)At all ANC visits

सभी एएनसी विजिट में

d)None

कोई नहीं

e)Don't Know

पता नहीं

1. Out of the given options, choose the correct nutritional advice that should be given to a woman with gestational hypertension:

दिए गए विकल्पों में से, सही पोषण संबंधी सलाह चुनें जो गर्भावस्‍था में हाइपरटेंशन से पीड़ित महिला को दी जानी चाहिए:

a)Low carbohydrate, high fat diet

कम कार्बोहाइड्रेट, उच्च फैट वाला आहार

b)Diet rich in proteins

प्रोटीन से भरपूर आहार

c)Low salt diet

कम नमक वाला आहार

d)None of the above

उपरोक्‍त में से सभी

e)Don't Know

पता नहीं

1. 26 yr., prim gravida at 22 weeks has BP reading 166/112 mm Hg, mark the correct statement about her management plan:

26 वर्ष की प्राइमिग्रेविडा कर 22 सप्ताह में बीपी की रीडिंग 166/112 mm Hg है, उसकी प्रबंधन योजना के बारे में सही वाक्‍य को मार्क करें:

a)She is low risk so can be given usual care.

उसे कम जोखिम है इसलिए उसे सामान्य देखभाल दी जा सकती है

b)She has hypertension, can be managed with medications alone.

उसे हाइपरटेंशन है, जिसका केवल दवाओं से उपचार किया जा सकता है।

c)She has the possibility of eclampsia, give necessary medications, and refer to the tertiary care center.

उसे एक्लेम्पसिया होने की संभावना है, आवश्यक दवाएं दें, और टर्शरी केयर सेंटर में रेफर करें।

d)None

कोई नहीं

e)Don't Know

पता नहीं

1. What would be your plan for 29 yrs, gravida 2, with normal BP but the history of hypertension during a previous pregnancy?

29 वर्ष की गर्भवती महिला, जिसका बीपी सामान्य है, लेकिन पिछली गर्भावस्था के दौरान हाइपरटेंशन का इतिहास है, उसके लिए आपकी क्या योजना होगी?

a)Monitor BP at every ANC visit

प्रत्येक एएनसी विजिट पर बीपी की जांच करना

b)Counselling on warning signs of gestational hypertension

गर्भावधि संबंधी हाइपरटेंशन के चेतावनी के संकेतों पर परामर्श देना

c)Start her on Cap. Nifedipine 10 mg or Tab. Labetalol 200 mg

उसे कैप्‍सूल निफेडिपिन 10 mg या टैबलेट लेबेटालोल 200 mg देना शुरू करना

d)Both a & b

a और b दोनों =4

e)All of the above

उपरोक्‍त सभी

*Add T aspirin 150mg in one of the option

*किसी एक विकल्प में टी एस्पिरिन 150mg जोड़ें

1. Do you think there is any advantage of early detection and diagnosis of hypertension in pregnancy?

क्या आपको लगता है कि गर्भावस्था में हाइपरटेंशन का जल्‍दी करने और डायग्‍नोसिस करने से कोई लाभ होता है?

a)Yes, it can avoid adverse effects on the fetus and the mother during labour

हां, इससे प्रसव के दौरान भ्रूण और मां पर पड़ने वाले प्रतिकूल प्रभाव से बचा जा सकता है।

b)No, the mother can be started on antihypertensive whenever detected during pregnancy

नहीं, गर्भावस्था के दौरान पता चलने पर माँ को एंटी हाइपरटेंसिव दवा दी जा सकती है

c)Not sure

पक्‍का नहीं

d)Don't Know

पता नहीं

1. Which of the following cases should be referred to a higher center:

इनमें से किस मामले को हायर सेंटर में भेजा जाना चाहिए:

a)All pregnant women with BP >= 160/110 mm Hg

सभी गर्भवती महिलाएं जिनका बीपी >= 160/110 mm Hg

b)Any pregnant woman with generalized oedema, BP >140/90mm Hg, and proteinuria (2+)

कोई भी गर्भवती महिला जिसमें इडीम सामान्यीकृत है, बीपी >140/90mm Hg, और प्रोटीनुरिया (2+) हो

c)All pregnant women who develop eclampsia-seizure/blurry vision/high BP

सभी गर्भवती महिलाएं जिनमें एक्लेम्पसिया-दौरा आना/धुंधली नज़रें/हाई बीपी विकसित होता है

d)All of the above

उपरोक्‍त सभी

1. Don't Know

पता नहीं

**Thank you for answering all the questions. We are looking forward to meet you during the training.**

**सभी प्रश्नों का जवाब देने के लिए धन्यवाद. हम ट्रेनिंग के दौरान आपसे मिलने के लिए उत्सुक हैं।**
