# Supplementary material for: Impact of a Multicomponent Intervention to Build Capacity of Public Health Workers to Make Algorithmic Diagnosis and Management of High-Risk Pregnancies in Uttar Pradesh, India: Protocol for a Matched-Control, Before-After, Quasi-Experimental Study With a Mixed Methods Design
Source: JMIR Res Protoc. 2025 Dec 9;14:e74993. doi: 10.2196/74993 (PMC12690279; doi:10.2196/74993)
Supplement: Multimedia Appendix 6 [file resprot-v14-e74993-s006.docx]

|  | | | | | | Confidential, information to be used for research purposes only | | | | | | | | | |  |
| --- | --- | --- | --- | --- | --- | --- | --- | --- | --- | --- | --- | --- | --- | --- | --- | --- |
|  | **Integrated High-Risk Pregnancy Tracking & Management (IHRPTM)**  **MAIN QUESTIONNAIRE FOR RECENTLY DELIVERED WOMEN** | | | | | | | | | | | | | | |  |
| **Respondent: RDW are woman who delivered during past 12 months at the time of survey.** | | | | | | | | | | | | | | | |  |
| I. District:_____________ | | | II. Block:________________ | | | | | III. Village:__________________ | | | | | | | |  |
| IV. Name of woman:________  IVa. Serial Number of the Women interviewed:____ | | | V. Name of father/husband of woman:_________ | | | | | Via. Date of birth of woman: ☐☐ ☐☐ ☐☐☐☐  Vib. Current age of woman: ☐☐ | | | | | | | |  |
| **VIIa Date of delivery:**  ☐☐ ☐☐ ☐☐☐☐  **VIIb Type of delivery:**  Normal Vaginal 1  Caesarean 2  Forceps 3  Other(specify) 99 | | | **VIII Place of delivery:**  Govt. hospital 1  Private Hospital 2  Home 3  On the way to hospital 4  Other (Specify) 99 | | | | | **IXa. If delivery was in Govt. hospital:**  Place of delivery (location):______  **IXb. Type of Govt. hospital:**  District hospital 1  Sub-district Hospital/Area hospital 2  CHC 3  PHC 4  Others 99 | | | | | | | |  |
| **X. Outcome of delivery:**  Live birth………..1  Stillbirth.………..2 🡪**XIIb** | | | XIa. Youngest Child name: _______  XIb. Infant’s age today (in days): ☐☐☐ | | | | | XIIa Weight of the baby at the time of birth: ☐:☐☐☐ in kg  Don’t Know……………………98  **XIIb. Term**  Preterm (<= 34 weeks) 1  Full term (> 34 weeks) 2 | | | | | | | |  |
|  | | | **XIII. MCP card availability**  Yes, available and shown to surveyor 1  Available, but DID NOT SHOW card 2  Not available 3  Others (Specify) 99 | | | | | **If 1 coded in XIII.**  **XIV.** Whether it was a HIGH-RISK PREGNANCY OR NOT as per MCP card (Check the MCP card and record the response)**:**  Yes, it was HRP as per card 1  No, it was not HRP as per card 2  Not recorded anything in card 3 | | | | | | | |  |
|  | | | **XV Whether MCP has the serial number: if yes then move to XV1, If no then skip XVI.**  **If 1 coded in XIII.**  **XVI. Copy mother’s identification number:** | | | | |  | | | | | | | |  |
|  | | | **Interview start time – Hour: Minute**  \|_______\|______\| : \|_____\|______\| | | | | | **Interview end time – Hour: Minute**  \|_______\|______\| : \|_____\|______\| | | | | | | | |  |
|  | | | | | | | | | | | | | | | |  |
|  | | | | | | | | |  | | |  |  | |  |  |
| **INTERVIEWER’S VISITS AND STATUS** | | | | | | | | | | | | | | | |  |
|  | | **Visit 1** | | | **Visit 2** | | **Visit 3** | | |  | **Final Visit** | | | | |  |
| **Date** | | \|_____\|_____\|-\|_____\|_____\|-\|_____\|_____\| | | | \|_____\|_____\|-\|_____\|_____\|-\|_____\|_____\| | | \|_____\|_____\|-\|_____\|_____\|-\|_____\|_____\| | | |  | **Date** \|_____\|_____\|-\|_____\|_____\|-\|_____\|_____\| | | | | |  |
| **Interviewer’s Name** | |  | | |  | |  | | |  | **Interviewer’s code** | | | \|_____\|_____\|_____\| | |  |
| **Result code*** | | \|_____\|_____\| | | | \|_____\|_____\| | | \|_____\|_____\| | | |  | **Result code** | | | \|_____\|_____\| | |  |
| **Next Visit** | | Date: | | Date: | | |  | | | | **Total no of visits** | | | \|_____\| | |  |
|  |  | Time: | | Time: | | |  |  |  |  |  |  |  |  |  |  |
| ***Result Codes:** 01 = Interview completed 02 = Respondent declined interview 03 = Time and date set for later  04 = Respondent not at home 97 = Other, specify: _____________ | | | | | | | | | | | | | | | |  |

**Section A: Birth History (Administer this section to all respondents)**

***Now I would like to ask about all the pregnancies you have had during your life.***

| NO. | **Questions** | **Codes** | **GO TO Q.** |
| --- | --- | --- | --- |
| A1 | How many daughters are alive? And how many sons are alive?  (How many children do you have? How many girls? And how many boys?) | D: Daughters alive \|____\|____\|  S: Sons alive \|____\|____\| |  |
| A2 | Have you ever given birth to a boy or girl who was born alive but later died? (Any baby who cried or showed signs of life but did not survive?) | Yes 1  No 2 | **🡫**A4 |
| A3 | How many boys have died? And how many girls have died? | G; Girls dead \|____\|____\|  B: Boys dead \|____\|____\| |  |
| A4 | Have you had any pregnancies that did not result in live births?  Some pregnancies end before full term as miscarriage or an abortion, while others may result in a stillbirth. | Yes 1  No 2 | **🡫**A6 |
| A5 | In all, how many pregnancies did not result in a live birth?  (Can be asked – Any miscarriages or abortions – and then ask them to specify in which month? If before 3 to 5 months mark as abortion, 7-9 months mark as IUD, if during delivery mark still birth) | A. (Pregnancy Loss & stillbirths) \|____\|____\|  B. No of abortions\|____\|____\|  C. No of **Intra Uterine Death (IUD)**  \|____\|____\|  D. No of still births \|____\|____\| |  |
| A6 | *[CHECK A1, A3 and A5, and ask]*  Just to make sure that I have this correct: you have had  ______# children still alive (A1)  ______# children have died (A3), and  ______# pregnancies which did not result in a live birth (A5)?  Is that correct?  **CAPI: (A1+A3+A5)** | Yes………………………………………1  No………………………………………..2  ***If no, then reconcile from A1, A3, and A5*** |  |
|  | *Comments/Remarks If any* |  |  |

**SECTION B : IS APPLICABLE IF 1 CODED IN XIII.**

**Section B: ANC during Last Pregnancy copied From MCP card**

**Recall serial number of questionnaire __________**

**Take photo of the MCP card (for this mother) the three pages using tablet and save the photo with SRNO as extension. Then this data needs to be entered into Excel/STATA, and link it with remaining data collected by speaking to the other/family.**

**Section C: Note down as elaborately as possible details of ANC visits made by RDW during entire pregnancy and also record details about the health problems faced by her - in her own words.**

**As part of discussion, specifically do probing on:**

**• By who & in which month, pregnancy was first confirmed?**

**• What was done when pregnancy was first confirmed?**

**• Was Scan/USG done any time & what was told to you about your health condition based on Scan/USG (if done). Why was it done? How many times was the sonography done?**

**• Please ask RDW to recall ALL the health problems/HRP conditions faced by her?**

**• Type(s) of problem(s)? Then probe on the other issues.**

**• When (which month of pregnancy) these problems were first diagnosed? Did you confirmed the problem by consulting other health personnel? Who diagnosed? What was done for diagnosed problem(s)? Who all provided management to diagnosed problem(s)?**

**• Any referral(s) made during pregnancy? Description of the referrals?**

- **Were you hospitalized anytime during pregnancy because of HRP? If yes, then why? (Detail)**
- **Whether health problem(s) got cured? Anybody made follow-up visits about persisting problem(s)?**

**s**

| **SECTION C: RECORD THE OPEN ENDED RESPONSES IN THE BELOW GIVEN GRID** | | | | | |
| --- | --- | --- | --- | --- | --- |
| **Main Question** | **Identification / diagnosis** | **Management** | **Counseling** | **Follow-up** |  |
| **Anemia** | Qc2_id - Which month of ANC Anemia was first diagnoses: ____  Qc2_id_1 - Type of anemia: Moderate / Severe  Qc2_id_2 - Hb level at the first time of diagnosis:_______  Qc2_id_3 - Who diagnosed^2^: ___ | Qc2_m - IFA given when anemia was diagnosed: Y/N  Qc2_m_1 - IFA dosage counselled: Yes/No  Qc2_m_2 - Whether Iron sucrose injection given anytime during pregnancy: Y/N  Qc2_m_3 – Was Blood Transfusion any time in pregnancy: Y/N | Qc2_c - Counseling on precautions to be taken: Y/N | Qc2_F1 - How many days stayed in Hosp after delivery: ___ |  |
| **BP (Hypertension)** | Qc3_id - Got BP problem prior to this pregnancy: Prior/In this pregnancy  Qc3_id_2 - Which month BP problem was diagnosed first time in this pregnancy: _____  Qc3_id_3 - Where was BP problem diagnosed first time^1^:  Qc3_id_4 - Who diagnosed^2^: | Qc3_m - Were you referred immediately after BP problem was diagnosed^1^: Y/N  Qc3_m_1 - Urine test done for confirmation of BP: Y/N  Qc3_m_2 - Who confirmed^2^: ___  Qc3_m_3 - Severity of problem:  Qc3_m_4 – Did you get pre-eclampsia/eclampsia (fits)  BP medicines changed (after confirmation): Y/N  Qc3_m_5 - Calcium dose provided: one/two | Qc3_c - Counseling done on Diet precautions or not: Y/N  Qc3_c_1 - Counseling on Other Precautions done or not: Y/N  Qc3_c_2 - Counseling on Preparedness for Delivery provided or not: Y/N  Qc3_c_3- what changes you made in your life after identification | Qc3_f - Repeat BP checkups done or not: Y/N  Qc3_f_1 - During further visits BP: Increased / decreased.  Qc3_f_2 - Now after delivery BP: Increased/ decreased  Qc3_f_3- How many days stayed in Hosp after delivery: ___ |  |
| **Bleeding** | Qc5_id - In which month bleeding problem first diagnosed:____  Qc5_id_1 - Which month bleeding problem diagnosed first time: _____  Qc5_id_2 - Where was bleeding problem diagnosed first time^1^:  Qc5_id_3 - Who diagnosed^2^:  Qc5_id_4 - Whether severity of bleeding assessed or not: Y/N  Qc5_id_5 - Did bleeding happened on emergency? Y/N | Qc5_m - Were you referred to higher facility to confirm bleeding problem: Y/N  Qc5_m_1 - If referred, where were you referred^1^:  Qc5_m_2 - Who confirmed bleeding problem on ^2^:  Qc5_m_3 - Medicines given: Y/N  Qc5_m_4 - Blood transfusion done any time during pregnancy: Y/N | Qc5_c - Counseling on Exercises given: Y/N  Qc5_c_1 - Counseling on Preparedness for Delivery provided or not: Y/N | Qc5_f - Present bleeding status of bleeding now: No/little/ still continuing  Qc5_f_1 - How many days stayed in Hosp after delivery: ___ |  |
| **Any other problem-1 diagnosed (specify):____** | Qc6_id - Which month problem was diagnosed first time: _____  Qc6_id_1 - Where was this problem diagnosed first time^1^:  Qc6_id_2 - Who diagnosed for first time^2^: | Qc6_m - Were you referred any time during pregnancy due to this problem: Y/N  Qc6_m_1 - If yes, where were you referred^1^:____  Qc6_m_2 - Who confirmed this problem^2^:  Qc6_m_3 - Medicines given for this problem: Y/N  Qc6_m_4 - Which medicine:_______ |  | Qc6_f - How many days stayed in Hosp after delivery: ___ |  |
| **Any other problem-2 diagnosed (specify):____** | Qc7_id - Which month problem was diagnosed first time: _____  Qc7_id_1 - Where was this problem diagnosed first time^1^:  Qc7_id_2 - Who diagnosed for first time^2^: | Qc7_m - Were you referred any time during pregnancy due to this problem: Y/N  Qc7_m_1 - If yes, where were you referred^1^:____  Qc7_m_2 - Who confirmed this problem^2^:  Qc7_m_3 - Medicines given for this problem: Y/N  Qc7_m_4 - Which medicine:_______ |  | Qc7_f - How many days stayed in Hosp after delivery: ___ |  |

1: **Where 1. ANM/HSC, 2. PHC, 3. CHC, 4. SDH/AH, 5. DH 6. Other Pub. Hosp 7. Pvt. Hosp 99. Other 77. Don’t remember**

2: **Who: 1. ASHA, 2. ANM, 3. SN, 4. MO (Gov), 5. OBGY (Gov), 6. MO (Pvt), 7. OBGY (Pvt), 99. Other 77. Don’t remember**

**Section D: Details of ANC during Recent Pregnancy**

**(Administer this section to all respondents)**

D1a. How many months pregnant were you when you came to know about the pregnancy? (enter number of months)

D1b. How many months pregnant were you when you registered? (enter number of months) (enter 99 if don’t know)

D1c. With whom did you register your pregnancy?

ASHA………1, ANM…………2, AWW……….3, Others…………4

D2: Can you please recall how many ANC visits in total you had when you were pregnant with-----(name):

**Number of ANC visits☐☐ No visits 0 Don’t remember…………….77**

D3: Can you please recall, what all examinations and tests were done during your first ANC visist.

| ***A. How many months were when you received 1^st^ ANC*** | ***B. Place of first ANC^a^*** | ***C. Who did first ANC^b^*** | ***D. Whether below examinations were done or not (Yes-1, No-2, unable to recall/don’t know-98)*** | | | | | | | | |
| --- | --- | --- | --- | --- | --- | --- | --- | --- | --- | --- | --- |
|  |  |  | ***a. Weight*** | ***b. Heamo-globin*** | ***c. BP*** | ***d. Pulse*** | ***f. Urine*** | ***g. Baby heart rate*** | ***h. Oedema*** | ***i. Abdomen*** | ***x. Other (specify)*** |
|  |  |  | ***1 2 98*** | ***1 2 98*** | ***1 2 98*** | ***1 2 98*** | ***1 2 98*** | ***1 2 98*** | ***1 2 98*** | ***1 2 98*** | ***1 2 98*** |

a: **Place of ANC: 1. ANM/HSC, 2. PHC, 3. CHC, 4. SDH/AH, 5. DH 6. Other Pub. Hosp 7. Pvt. Hosp 99. Other 77. Don’t remember**

b: **Who mainly did ANC check-up: 1. ASHA, 2. ANM, 3. SN, 4. MO (Gov), 5. OBGY (Gov), 6. MO (Pvt), 7. OBGY (Pvt), 99. Other 77. Don’t remember**

| D3D.1 | Whether **Weight** was measured in subsequent ANCs?  If Yes, how many times? | Yes 1  No 2  If YES how many times: ------ |  |
| --- | --- | --- | --- |
| D3 D.1 a | Do you know how much weight you have gained during your entire pregnancy? | Yes………1  No……….2 | If coded 1 then ask D3.D1 b |
| D3 D.1 b | If yes, how much weight you have gained during pregnancy in Kgs. | ----Kg |  |
| D3D.2 | Whether ***Heamo-globin*** was tested in subsequent ANCs?  If Yes, how many times? | Yes 1  No 2  If YES, how many times: ------ |  |
| D3D.3 | Whether **blood pressure** (***BP)*** was tested in subsequent ANCs?  If Yes, how many times? | Yes 1  No 2  If YES, How many times: --- |  |
| D3D.4 | Whether **Pulse** was tested in subsequent ANCs?  If Yes, how many times? | Yes 1  No 2  If YES, how many times: |  |
| D3D.5 | Whether **urine** was tested in subsequent ANCs?  If Yes, how many times? | Yes 1  No 2  If YES, how many times: |  |
| D3D.6 | Whether **Baby heart beat** was tested in subsequent ANCs?  If Yes, how many times? | Yes 1  No 2  If YES, how many times: |  |
| D3D.8 | Whether **examined Abdomen** in subsequent ANCs?  If Yes, how many times? | Yes 1  No 2  If YES, how many times: |  |

D4. During pregnancy women experience/undergo certain health problem which are common. These health problems are mild in some women and severe in some others. I wish to know your experience when you were pregnant with ----------------(name of last child). Kindly recall and tell me about your experience for each one of the following.

| Q.No | Did you experience the health issue like……… | | Was this health issue asked and recorded by the ANM during the any ANC visits? |
| --- | --- | --- | --- |
| D4.a | Nausea and vomiting | Yes- 1  No- 2 | Yes-1; No-2 |
| D4.b | Heartburn | Yes- 1  No- 2 | Yes-1; No-2 |
| D4.c | Constipation | Yes- 1  No- 2 | Yes-1; No-2 |
| D4.d | Increased frequency of urination | Yes- 1  No- 2 | Yes-1; No-2 |
| D4.e | Abdominal pain | Yes- 1  No- 2 | Yes-1; No-2 |
| D4.f | Vaginal Bleeding P/V | Yes- 1  No- 2 | Yes-1; No-2 |
| D4.g | Vaginal Discharge | Yes- 1  No- 2 | Yes-1; No-2 |
| D4.h | Fever with Rash | Yes- 1  No- 2 | Yes-1; No-2 |
| D4.i | Convulsions | Yes- 1  No- 2 | Yes-1; No-2 |
| D4.j | Swelling of the legs, body or face | Yes- 1  No- 2 | Yes-1; No-2 |
| D4.k | Difficulty with vision during daylight | Yes- 1  No- 2 | Yes-1; No-2 |

| NO. | **Questions** | **Codes** | **GO TO Q.** |
| --- | --- | --- | --- |
| D5 | a. Were you asked about **last mensural period (LMP)** by ANM ?  b. Were you told about **Expected Date of delivery (EDD)** by ANM? | Yes-1; No-2  Yes-1; No-2 |  |
| D8 a | During (any of) your antenatal care visit(s), were you told about the following **signs of**  **pregnancy complications?**  READ OUT THE OPTIONS  (**CAPI INSTRUCTION**:  Please GIVE separate questions on separate screens) | 1. Vaginal Bleeding Y N 2. Convulsions Y N 3. Prolonged Labour Y N 4. Severe Abdominal Pain Y N 5. High Blood Pressure (BP) Y N |  |
| D8 b | Were you told where to go if you had any pregnancy complications? | Yes – 1; No – 2 |  |
| D8 c | Was (NAME's) father present during (any of) your antenatal visit(s)? | Yes – 1; No – 2 |  |
| D8 d | During this pregnancy, were you given any injection in the arm to prevent the baby from getting **tetanus**, that is, convulsions after birth?  | Yes - 1  No – 2  Not Sure -3  |  |
| D8e | Ask if 1 coded in D8d  During this pregnancy, how many times did you get a tetanus injection? | Number of times …________ (MAX 3)  Do not remember…77 |  |
| D8 i | During this pregnancy, were you given or did you buy any iron folic acid tablets or syrup? | YES Tablets 1  YES syrup 2  Both 3  NO 4 Don't know 8 | D8 l |
| D8 j1 | How many iron folic acid tablets/doses of syrup  did you consume per day during your pregnancy? | Number of tablets Per day -------- (Ask if 1/3 coded in D8J)  Number of Syrup doses Per day-------(Ask if 2/3 coded in D8J) | If 0 in both SKIP to D8L |
| D8j2 | During the whole pregnancy, for how many days did you take IFA tablets or syrup? | Number of Days -------- |  |
| D8 k | In which month of your pregnancy did you start taking iron and folic acid supplements and in which month did you stop taking the IFA supplementation? | Starting ------th month  Ending -------th month |  |
| D8 l | During this pregnancy, did you take any drugfor intestinal worms? | Yes 1  No 2  Don’t Know 8 |  |
| D8 m | During this pregnancy, did you use a mosquito net regularly, sometimes or never? | Regularly 1  Sometimes 2  Never 3 |  |
| D9a | Did you receive any supplementary nutrition from the Anganwadi center during this pregnancy? | Yes 1  No 2 |  |
| D9b | If Yes in D9a, did you receive supplementary nutrition | Always – 1  Always or occasionally? Occasionally – 2 |  |
| D10a | During the last three months of this  pregnancy, Where did you go for ANC checkup | Not visited 0  HSC 1  PHC(MO) 2  CHC(MO/Spl) 3  Sub-District Hospital 4  District Hospital and above 5  Private hospital/Clinic 6  Others (specify) 7 |  |
| D11 | If you missed any ANC visit, what were the reasons for missing that ANC visit?  **(Multiple response question)** | Bad experiences A  Excessive waiting times B  Staff unavailability C  Lack of medicines D  Lack of convenient appointment times E  Did not receive timely notification from health staff F  Too far from govt facility G  Lack of transportation H  Lack of money to travel I  No support from family to accompany woman J  Moved to paternal home K  Did not miss L  Other (Specify) X |  |
| D13 | On what all topics you were Counselled or provided health education?  **(Multiple response question)** | Nutrition……………………….Y/N  Initiation of Breastfeeding within one hour of birth…………………….Y/N  Exclusive breastfeeding till six months after birth………………………..Y/N  Contraception……………………YN  **Other (specify)…………………Y/N** |  |

D15: Please once again recall what all health problems or High-Risk Pregnancy (HRP) problems did you suffer when you were pregnant with (ChildName)?

***Instruction:*** ***Initially ask her to recall all the health problems. Then quickly ask whether she had any of the listed problems one by one, excluding the ones she has already stated. Finally, match responses in below table with information she has provided in open ended question, and modify accordingly. SEPARATE SECTIONS FOR SPONT AND AIDED***

| **S.No** | **Type of high-risk condition or health problem** | **Duration of high-risk** | |
| --- | --- | --- | --- |
|  |  | **Month of first diagnosis**  **(Record month of pregnancy)** | **Till which month the problem continued**  **(Record month of pregnancy)** |
| 1 | **Moderate Anaemia**  **(ASK-** Did you have low blood percentage during pregnancy as suggested by the health provider**)** |  |  |
| 2 | **Severe Anaemia**  **(ASK-** Were you having severely low blood percentage during pregnancy) or were you informed of severely low blood percentage during pregnancy |  |  |
| 3 | **Pregnancy Induced Hypertension (PIH)**  **(ASK –** Did you have B.P during pregnancy) |  |  |
| 4 | **Ante Partum Haemorrhage (APH)**  **(ASK –** Did you have bleeding issues after 6 months of pregnancy) |  |  |
| 5 | **Gestational Diabetes Mellitus (GBD)**  **(ASK –** Did you have sugar (Common word for diabetes) during pregnancy) |  |  |
| 6 | **COVID** |  |  |
| 7 | **Epilepsy** (Ask regarding fits, before/during pregnancy) |  |  |
| 8 | **Pregnancy with Heart Disease** |  |  |
| 9 | **Tuberculosis (TB)** |  |  |
| 10 | **Jaundice/liver disease** |  |  |
| 11 | **Pregnancy with Previous Lower Segment Caesarean Section (LSCS)**  **(ASK-** Did you undergo operation to deliver the baby during your previous pregnancy?) |  |  |
| 12 | **Shortness of breath**  **(ASK-** Did you experience significant breathing difficulties that led you to seek medical attention?) |  |  |
| 13 | **Fever** |  |  |
| 14 | **Preterm labour/Preterm Premature Rupture of Membrane (PPROM)**  **(ASK-** Did you deliver the baby before full term) |  |  |
| 15 | **Pregnancy with Intra Uterine Growth Retardation (IUGR)**  **(ASK**- Did doctors mention that your baby was not growing properly/ underweight during pregnancy**)** |  |  |
| 16 | **Bleeding before 20 weeks of pregnancy**  (Specify -Bleeding before 5 months) |  |  |
| 17 | **Decreased foetal movements/ Intra Uterine Death (IUD)** |  |  |
| 18 | **Human Immunodeficiency Virus (HIV)** |  |  |
| 19 | **Thyroid** |  |  |
| 20 | **Any other-1 (please specify) _______________** |  |  |
| 21 | **Any other-2 (please specify) _______________** |  |  |

***Kindly check if RDW responded of having:***

***Moderate anaemia……….1***

***Severe anaemia…………..2***

***BP……………………….3***

***APH………………………4***

**D16: ONLY FOR ABOVE LISTED HEALTH PROBLEMS/HRPs FILL THE RESPECTIVE ROWS**

For above diagnosed problem(s), were you referred from the place it was first diagnosed to a higher facility or not? If referred, from which facility to which facility? Problem resolved or not?

| **S.No** | **Type of high-risk condition** | **Referred or not?**  **1-Yes, 2-No** | **From where you were referred** | **To where you were referred** | **From 1^st^ referral were you referred to further higher facility**  **1-Yes, 2-N0** | **If yes, to where?** | **Was the Problem resolved?**  **1-Yes, 2-N0** |
| --- | --- | --- | --- | --- | --- | --- | --- |
| 1 | **Moderate Anaemia**  **(ASK-** Did you have low blood percentage during pregnancy as suggested by the health provider**)** | Yes:◻🡪  No: ◻ | ◻ | ◻ | Yes..1  No…2 | ◻ | Yes..1  No…2 |
| 2 | **Severe Anaemia**  **(ASK-** Were you having low blood percentage during pregnancy) | Yes:◻🡪  No: ◻ | ◻ | ◻ | Yes..1  No…2 | ◻ | Yes..1  No…2 |
| 3 | **Pregnancy Induced Hypertension (PIH)**  **(ASK –** Did you have B.P during pregnancy) | Yes:◻🡪  No: ◻ | ◻ | ◻ | Yes..1  No…2 | ◻ | Yes..1  No…2 |
| 4 | **Ante Partum Haemorrhage (APH)**  **(ASK –** Did you have bleeding issues after 6 months of pregnancy) | Yes:◻🡪  No: ◻ | ◻ | ◻ | Yes..1  No…2 | ◻ | Yes..1  No…2 |
| 5 | **Gestational Diabetes Mellitus (GBD)**  **(ASK –** Did you have sugar (Common word for diabetes) during pregnancy) | Yes:◻🡪  No: ◻ | ◻ | ◻ | Yes..1  No…2 | ◻ | Yes..1  No…2 |
| 6 | **COVID** | Yes:◻🡪  No: ◻ | ◻ | ◻ | Yes..1  No…2 | ◻ | Yes..1  No…2 |
| 7 | **Epilepsy** (Ask regarding fits, before/during pregnancy) | Yes:◻🡪  No: ◻ | ◻ | ◻ | Yes..1  No…2 | ◻ | Yes..1  No…2 |
| 8 | **Pregnancy with Heart Disease** | Yes:◻🡪  No: ◻ | ◻ | ◻ | Yes..1  No…2 | ◻ | Yes..1  No…2 |
| 9 | **Tuberculosis (TB)** | Yes:◻🡪  No: ◻ | ◻ | ◻ | Yes..1  No…2 | ◻ | Yes..1  No…2 |
| 10 | **Jaundice/liver disease** | Yes:◻🡪  No: ◻ | ◻ | ◻ | Yes..1  No…2 | ◻ | Yes..1  No…2 |
| 11 | **Pregnancy with Previous Lower Segment Caesarean Section (LSCS)**  **(ASK-** Did you undergo operation to deliver the baby during your previous pregnancy?) | Yes:◻🡪  No: ◻ | ◻ | ◻ | Yes..1  No…2 | ◻ | Yes..1  No…2 |
| 12 | **Shortness of breath**  **(ASK-** Did you experience significant breathing difficulties that led you to seek medical attention?) | Yes:◻🡪  No: ◻ | ◻ | ◻ | Yes..1  No…2 | ◻ | Yes..1  No…2 |
| 13 | **Fever** | Yes:◻🡪  No: ◻ | ◻ | ◻ | Yes..1  No…2 | ◻ | Yes..1  No…2 |
| 14 | **Preterm labour/Preterm Premature Rupture of Membrane (PPROM)**  **(ASK-** Did you deliver the baby before full term) | Yes:◻🡪  No: ◻ | ◻ | ◻ | Yes..1  No…2 | ◻ | Yes..1  No…2 |
| 15 | **Pregnancy with Intra Uterine Growth Retardation (IUGR)**  **(ASK**- Did doctors mention that your baby was not growing properly/ underweight during pregnancy**)** | Yes:◻🡪  No: ◻ | ◻ | ◻ | Yes..1  No…2 | ◻ | Yes..1  No…2 |
| 16 | **Bleeding before 20 weeks of pregnancy**  (Specify -Bleeding before 5 months) | Yes:◻🡪  No: ◻ | ◻ | ◻ | Yes..1  No…2 | ◻ | Yes..1  No…2 |
| 17 | **Decreased foetal movements/ Intra Uterine Death (IUD)** | Yes:◻🡪  No: ◻ | ◻ | ◻ | Yes..1  No…2 | ◻ | Yes..1  No…2 |
| 18 | **Human Immunodeficiency Virus (HIV)** | Yes:◻🡪  No: ◻ | ◻ | ◻ | Yes..1  No…2 | ◻ | Yes..1  No…2 |
| 19 | **Thyroid** | Yes:◻🡪  No: ◻ | ◻ | ◻ | Yes..1  No…2 | ◻ | Yes..1  No…2 |
| 20 | **Any other-1 (please specify) _______________** | Yes:◻🡪  No: ◻ | ◻ | ◻ | Yes..1  No…2 | ◻ | Yes..1  No…2 |
|  | *Comments/Remarks If any* |  | | | | | |

a: **Place of referral : 1. ANM/HSC, 2. PHC, 3. CHC, 4. SDH/AH, 5. DH 6. Other Pub. Hosp 7. Pvt. Hosp 99. Other 77. Don’t remember**

**Section E: Delivery & New-born care particulars**

**(Administer this section to all respondents)**

| NO. | **Questions** | **Codes** | **GO TO Q.** |
| --- | --- | --- | --- |
| E1 | During delivery with (Child Name), did you experience a breech presentation? | Yes 1  No 2 |  |
| E2 | During delivery, did you experience prolonged labour?  Def: when the combined duration of the first and second stage is more than the arbitrary time limit of 18 hours | Yes 1  No 2 |  |
| E3 | During delivery, did you experience excessive bleeding? (As they recall Doctor’s advice – probe: Had to perform blood transfusion?)  *Def:* loss of more than 2 points of blood or symptoms of significant blood loss (500 ml) that occur within 24 hours of delivery | Yes 1  No 2 |  |
| E3a | Had to perform blood transfusion within 24 hours of delivery? | Yes 1  No 2  Did not remember 97 |  |
| E4 | **Ask if it is institutional delivery (VIII on cover page)**  What was the main mode of transportation used by you to reach the health facility for delivery? | Govt. Ambulance 1  Other ambulance 2  Jeep/car 3  Motor cycle/scooter 4  Bus/Train 5  Temp/Auto/Tractor 6  Cart 7  On foot 8  Other(specify) 99 |  |
| E5 | Total (transportation, hospital stay, tests, medicines, food, other costs) how much did it cost for your delivery? | Rs…………………………… ☐☐☐☐☐☐(Approx.) |  |
| E6 | Did you receive any financial assistance for delivery care? | Yes 1  No 2  Don’t know 98 | 🡪E8  🡪E8 |
| E7 | If yes, How much financial assistance did you receive after delivery? | Rs…………………………… ☐☐☐☐☐☐ |  |
| E8 | Did you receive delivery kit after the delivery? | Yes 1  No 2 |  |
| E9 | How long after (NAME) was delivered did you stay in the health facility?  **IF LESS THAN ONE DAY, RECORD IN HOURS**  **IF LESS THAN ONE WEEK RECORD IN DAYS**  **IF ONE WEEK OR MORE IN WEEKS** | 1. HOURS: ☐☐ 2. DAYS: ☐☐ 3. WEEKS: ☐☐   Don’t Know 98 |  |
|  | **Ask E9a-c if it is institutional delivery (VIII on cover page)** | | |
| E9a | When did you go to hospital for delivery?  Record date and time in 24 hours format | Date: DD/MM/YYYY  Time: MM:HH |  |
| E9b | When did you deliver the baby?  Record date and time in 24 hours format | Date: DD/MM/YYYY  Time: MM:HH |  |
| E9c | When did you leave the hospital after the delivery?  Record date and time in 24 hours format | Date: DD/MM/YYYY  Time: MM:HH |  |
|  | **ASK E10 & 11 ONLY IF LIVE BIRTH (X on cover page)OTHERWISE SKIP TO E12** | |  |
| E10 | Was (Child name) born with any congenital anomalies? | Yes 1  No 2  Don’t know 98 |  |
| E11 | Was --------------(name) admitted in hospital or in NICU during the first 4 weeks of birth for any severe health issues? | Admitted only in hospital 1  Admitted only in NICU 2  Both 3  No admission 4 |  |
| E12 | Were you admitted in hospital within 4 weeks of birth for any health issues after coming home? | Yes 1  No 2 |  |
| E13 | In case C-Section (Refer **VIIb coded 2 on cover page)**  When was the decision made for you to have a C-section? Was it before the onset of labour or after the onset of labour? | Before onset of labour (Elective) 1  After onset of labour (Emergency) 2  Don’t know 3 |  |
| E13a | **If Elective** **(1 coded in E13)**  What are the reasons for having C-section?  MULTIPLE RESPONSE POSSIBLE | Fear of Labour pain A  Previous birth was C-section B  Safer for baby C  Convenience in Scheduling birth D  Suggested by Health provider during ANC E  Self / Family Decision F  Prior Traumatic birth G  Others (Specify) |  |
| E14a | **If Emergency (2 coded in E13)**  What are the reasons for having C-section?  MULTIPLE RESPONSE POSSIBLE | Hypertension A  Diabetes B  Failure to progress/prolonged labour C  Umbilical cord stuck around the neck D  Heavy bleeding E  Breech presentation F  Safer for baby G  Others (Specify) X |  |
|  | *Comments/Remarks If any* |  |  |

**Section F: Only for RDW with MODERATE/SEVERE ANAEMIA**

**(If recorded as M/S anaemia in open question/MCP card/D15 question)**

***Anaemia definition: Moderate if Hb% is 7-9.9gm/dL; Severe if Hb% is 5-6.9gm/dL***

| ***No.*** | ***Questions*** | ***Codes*** | ***GO TO Q.*** | | |
| --- | --- | --- | --- | --- | --- |
|  | **Identification:** |  |  | | |
| F1 | Is it severe or moderate anaemia as per what she recalls? MCP card?  (CAPI - auto recode from d15/section C) | Moderate anaemia 1  Severe anaemia 2 |  | | |
| F2 | At what stage of pregnancy / delivery problem of moderate/severe anaemia was first detected? | During pregnancy 1  At the time of delivery 2 |  | | |
| F2A | If During Pregnancy, in which month of pregnancy | Month: ______ |  | | |
| F3 | Who diagnosed the moderate/severe anaemia problem for the first time? | ANM 1  MO at PHC 2  MO/Specialist at CHC 3  MO/Specialist at Dist. Hosp or above 4  private hospital 5  Don’t remember 77  Other (specify) 99 |  | | |
| F4 a | Haemoglobin (Hb) readings by MCP card | Not recorded in MCP card 0  Note all the values recorded:  1^st^ visit ___________  2^nd^ visit ___________  3^rd^ visit ___________  4^th^ visit ___________  5^th^ visit ___________  6^th^ visit ___________  7^th^ visit ___________  8^th^ visit ___________ |  | | |
| F4 b | Haemoglobin (Hb) readings as recalled by mother | Note all the values recalled:  1^st^ visit ___________  2^nd^ visit ___________  3^rd^ visit ___________  4^th^ visit ___________  5^th^ visit ___________  6^th^ visit ___________  7^th^ visit ___________  8^th^ visit ___________ |  | | |
| F5 | Did the ANM/heath personal inquired about the following health issues during ANC period  READ OUT | Fatigue Y/N  Palpitations Y/N  Pallor Y/N  Shortness of Breath Y/N  Giddiness Y/N  History of blackouts, pedal oedema, swelling all over body Y/N  Any History of PPH in previous pregnancies Y/N  Any History of of blood transfusions Y/N  Any History of fever with chills Y/N  History of TB Y/N  History of Sicklcell anaemia Y/N  Other (specify) Y/N |  | | |
| F5 a | **Ask if A6 Total Preg > 2**  Was this your 3^rd^ or above pregnancy | Yes 1  No 2 |  | | |
| F5 b | **Ask if A6 Total Preg > 1**  Were the spacing between previous deliveries was less than two years | Yes 1  No 2 |  | | |
|  | **Management:** |  |  | | |
| F6 a | Where was Anaemia problem managed for the first time | Home 1  Subcentre 2  PHC 3  CHC 4  SDH/AH 5  DH 6  MC 7  MCH 8  Private hospital 9 |  | | |
| F6 b | Who managed it subsequently?  **MULTIPLE RESPONSE POSSIBLE** | ANM A  MO at PHC B  Staff nurse at PHC C  MO/specialist at CHC D  Mo/ specialist at district hospital or above E  Private hospital F  Don’t remember G  Others (specific) X |  | | |
| F7 | How many IFA tablets per day were prescribed to you? | One……………………………1  Two……………………………2  None…………………………..00  Don’t remember………………77 |  | | |
| F8 | When were you given Albendazole tablet? | Month of Pregnancy _____  Did not take Albendazole……….00  Don’t remember…………………77 |  | | |
| F9 | Were you given any Iron sucrose injections through IV during your pregnancy? | Yes 1  No 2  Don’t Know 98 | 🡪F12  🡪F12 | | |
| F10 | If yes, When did they administer Iron Sucrose IV?  NOTE: ASK FOR MONTH OF PREGNANCY AND ACCORDINGLY CODE | 1^st^ trimester ……………………1  2^nd^ trimester……………………2  3^rd^ trimester ……………………3  Don’t remember………………..77 |  | | |
| F11 | If yes, How many doses of Iron Sucrose were administered? Specify number | ☐☐ |  | | |
| F12 | Were you suggested for any blood transfusion? | Yes 1  No 2  Don’t Know 98 | 🡪F15  🡪F15 | | |
| F13 | If yes, when did they recommend blood transfusion?  NOTE: ASK FOR MONTH OF PREGNANCY AND ACCORDINGLY CODE | 1^st^ trimester ……………………1  2^nd^ trimester……………………2  3^rd^ trimester ……………………3  During delivery…………………4  Don’t remember………………..77 |  | | |
| F14 | If yes, How many units of blood did they transfuse? Specify number | ☐☐ |  | | |
|  | **Counselling** |  |  | | |
| 17A | Did you receive counselling after treatment to Anaemia? | Yes 1  No 2 |  | | |
| 17B | Who provided counselling?  **MULTIPLE RESPONSE POSSIBLE** | ASHA A  ANM B  Medical Officer C  Staff Nurse D  Specialist E  Others (specify) X  None Y |  | | |
| 17C | On what all issues counselling was provided?  **PROBE FOR ANY OTHER?**  **Multiple response possible** | Dietary habits A  IFA use B  HB testing C  Sensitise about warning signs D  Rest E  Others X |  | | |
| F17 | **ASK, IF A CODED IN F17C**  What kind of dietary counselling was given to you when you were identified for Severe/Moderate Anaemia?  **(Multiple response question)** | **Increased intake of -**  Cereals A  Milk products B  Green leafy vegetables C  Pulses D  Eggs E  Meat F  Groundnuts G  Ragi H  Jaggery I  Lemon J  Guava K  Oranges L  Amla M  Others X |  | | |
| F18 | **ASK, IF B CODED IN F17C**  What type of IFA related Counselling did you receive?  S – Spontaneous 1  P – Prompted 2  N- No answer 3 | a. Side effects like Nausea, constipation and black stools  b. Avoid with calcium supplements  c. Importance of IFA tablets during pregnancy  d. Iron tablet- 1hr after meal  e. Calcium- 2 hrs after meal  x. Others | S  1  1  1  1  1  1 | P  2  2  2  2  2  2 | N  3  3  3  3  3  3 |
|  | **Referral** |  |  | | |
| F21 | Was Hb% test repeated after one month of severe/moderate anaemia diagnosis? | Yes 1  No 2  Don’t remember 77 |  | | |
| F21a | Did you observe improvement in the Hb level? | Yes 1  No 2 |  | | |
| F22 | IF YES IN F21a, What was the extent of change observed in Hb% after a month? | More than 1g/dl 1  Less than 1g/dl 2  No change 3  Don’t remember 77 |  | | |
| F23 | What was advised/suggested to you further?  **(Multiple response question)** | IFA continued A  IV Iron Sucrose B  Blood transfusion C  Don’t remember Z |  | | |
| F23a | Were you referred to any higher health facility? | Yes 1  No 2 | 🡪F24 | | |
| F23b | Where were you referred?  **Codes**  Subcentre………………….1  PHC………………………...2  CHC…………………………3  SDH/AH………………….……….…4  DH……………………………..5  MC……………..……………..6  MCH……………..………….7  Private hospital…………8 | Referred From ________________  Referred to____________________  (CAPI NOTE: PLEASE CHECK THE CODE IN REFERRED TO SHOULD BE HIGHER THAN REFERRED FROM) |  | | |
| F23c | Which month were you referred during your pregnancy? | Month of pregnancy ______ |  | | |
| F23d | How long did it take for you to visit the above the referral facility? | Immediately/ same day 1  Next day 2  After 3-4 days 3  After a week 4  On emergency 5  Not visited the referred facility 0 |  | | |
| F23e | Were you referred to any other higher health facility after first referral? | Yes 1  No 2 | 🡪F24a | | |
| F23d | If Yes in F23e, Where were you referred?  **Codes**  Subcentre…………………. 1  PHC………………………. 2  CHC……………………… 3  SDH/AH………………….…… 4  DH……………………… 5  MC……………..………… 6  MCH……………..…… 7  Private hospital…… 8 | Referred From ________________  Referred to____________________  (CAPI NOTE: PLEASE CHECK THE CODE IN REFERRED TO SHOULD BE HIGHER THAN REFERRED FROM) |  | | |
|  | **Follow up** |  |  | | |
| F24a | Were you followed up after coming back from the referral facility? | Yes 1  No 2 | 🡪F24 | | |
| F24b | Who followed up? | ANM 1  MO at PHC 2  MO/Specialist at CHC 3  MO/Specialist at Dist. Hosp or above 4  Doctor at Private hospital 5  Don’t remember 77  Other (specify) 99 |  | | |
| F24 | Were you advised to take IFA **post-delivery**? | Yes 1  No 2 | 🡪F25 | | |
| F24c | If Yes in F24, Are you taking IFA tablets now or Did you take IFA tablets **post-delivery**?  (**CAPI NOTE**: IF 2 CODED ALLOW TO RECORD THE NUMBER OF MONTHS) | Continuing 1  Taken till month ________ 2  Did not consume 3 |  | | |
| F25 | Were your Anaemia problem resolved before delivery? | Yes 1  No 2 | 🡪F27 | | |
| F26 | If NO, what was done? | RECORD VERBATIM |  | | |
| F27 | What extent you were satisfied with the management / referral services you have received to address the anaemia problem? | Very Satisfied 5  Satisfied 4  Neither satisfied nor satisfied 3  Not satisfied 2  Not at all satisfied 1 |  | | |
|  | *Comments/Remarks If any* |  |  | | |

**Section G: Only for RDW with Hypertension**

**(If recorded as having hypertension problem in Open question/MCP card/D15 question)**

***Definition of Hypertension in Pregnancy (If BP > 140/90):***

| ***No.*** | ***Questions*** | ***Codes*** | | | ***GO TO Q.*** |
| --- | --- | --- | --- | --- | --- |
|  | **Identification** |  | | |  |
| G1 | Was RDW diagnosed with Hypertension /BP problem prior to current pregnancy or during current pregnancy? | General Hypertension (Before Pregnancy) 1  Identified during current pregnancy 2 | | | 🡪G4 |
| G2 | **ASK G2 & G3 IF 1 CODED IN G1**  If General Hypertension: Identified first time at the age (in yrs.) | ☐☐ | | |  |
| G3 | Was there any Change in medication for high BP, during current pregnancy? | Yes 1  No 2 | | |  |
| G4A | **ASK IF 2 CODED IN G1**  At what stage of pregnancy / delivery problem of hypertension was first detected? | During pregnancy 1  At the time of delivery 2 | | | 🡪G5 |
| G4B | If During Pregnancy, in which month of pregnancy | Month:______ | | |  |
| G5 | Who diagnosed Hypertension for the first time? | ANM 1  MO at PHC 2  MO/Specialist at CHC 3  MO/Specialist at Dist. Hosp or above 4  Private hospital 5  Don’t remember 77  Other (specify) 99 | | |  |
| G5 a | Record BP recording from MCP card | Not recorded in MCP card 0  Note all the values recorded/recalled:  1^st^ visit ___________  2^nd^ visit ___________  3^rd^ visit ___________  4^th^ visit ___________  5^th^ visit ___________  6^th^ visit ___________  7^th^ visit ___________  8^th^ visit ___________ | | |  |
| G6 | BP recordings –recalled by respondent? | Note all the values recorded/recalled:  1^st^ visit ___________  2^nd^ visit ___________  3^rd^ visit ___________  4^th^ visit ___________  5^th^ visit ___________  6^th^ visit ___________  7^th^ visit ___________  8^th^ visit ___________ | | |  |
| G7 | Was Urine Dip stick test conducted? | Yes 1  No 2 | | |  |
| G9 | Ask if more than one pregnancies  When diagnosed with BP, whether the following information was collected or not?  **Read out the responses** | Previous pregnancy losses like Abortions/ Intrauterine Death/ Still birth **Y/N**  High BP in previous pregnancy **Y/N**  Preeclampsia/eclampsia in previous pregnancy **Y/N**  Other (specify) **Y/N** | | |  |
| G10 | Along with BP, whether any other high-risk problems identified?  **Read out the responses** | Age less than 20 yrs **Y/N**  Age more than 35 yrs **Y/N**  Overweight (BMI >25) /obesity (BMI > 30) **Y/N**  Pregnancy interval more than 10 yrs **Y/N**  Family history of hypertension in mother  /sister **Y/N**  Pre-existing chronic hypertension, kidney disease, diabetes (on medications) **Y/N**  Other (specify) **Y/N** | | |  |
|  | **Management** |  | | |  |
| G11 | Where was BP Managed during the pregnancy?  **(Multiple response question)** | Subcentre A  PHC B  CHC C  SDH/AH D  DH E  MC F  MCH G  Private hospital H  Home remedies…………………………I | | |  |
| G11a | Who managed the BP during your pregnancy?  **(Multiple response question)** | ANM A  MO at PHC B  MO/Specialist at CHC C  MO/Specialist at Dist. Hosp or above D  Private hospital E  Don’t remember Z  Other (specify) X | | |  |
| G12 | What were the symptoms did you experience and shared with health provider during pregnancy?  **(READ OUT)**  Yes Y  No N | a. Generalized oedema particularly non- dependent  b. Sudden onset headache  c. Vomiting  d. Pain in upper abdomen (epigastric)  e. Blurring of vision  f. Excessive weight gain (> 0.5 kg/week)  g. Any growth issues in the foetus observed  h. Experienced fits (Eclampsia) | Experienced  Y N  Y N  Y N  Y N  Y N  Y N  Y N  Y N | Shared  Y N  Y N  Y N  Y N  Y N  Y N  Y N  Y N |  |
|  | **Referral** |  | | |  |
| G13a | Were you referred to any higher health facility? | Yes 1  No 2 | | | 🡪G14 |
| G13b | Where were you referred?  **Codes**  Subcentre 1  PHC 2  CHC 3  SDH/AH 4  DH 5  MC 6  MCH 7  Private hospital 8 | Referred From ____________________  Referred to_______________________  (CAPI NOTE: PLEASE CHECK THE CODE IN REFERRED TO SHOULD BE HIGHER THAN REFERRED FROM) | | |  |
| G13c | Which month were you referred during your pregnancy? | Month of pregnancy ______ | | |  |
| G13d | How long did it take for you to visit the above the referral facility? | Immediately/ same day 1  Next day 2  After 3-4 days 3  After a week 4  On emergency 5  Not visited the referred facility 0 | | |  |
| G13e | Were you referred to any other higher health facility after first referral? | Yes 1  No 2 | | |  |
| G13d | Where were you referred?  **Codes**  Subcentre 1  PHC 2  CHC 3  SDH/AH 4  DH 5  MC 6  MCH 7  Private hospital 8 | Referred From ____________________  Referred to_______________________  (CAPI NOTE: PLEASE CHECK THE CODE IN REFERRED TO SHOULD BE HIGHER THAN REFERRED FROM) | | |  |
| G14 | What type of medicine was provided for the BP problem? (Check MCP card) | Nifedipine 1  Methyldopa 2  Labetalol 3  Others (specify) 99  None 00 | | |  |
| G15 | In case of emergency (fits due to High BP), where did you go for treatment?  **(Multiple response questions)** | Subcentre A  PHC B  CHC C  SDH/AH D  DH E  MC F  MCH G  Private hospital H  None (no fits) Y | | | 🡪G17 |
| G16 | If you have experienced fits due to high BP, were you given any Injection to the buttocks before referring you to the higher facility? | Yes 1  No 2  Don’t remember 77  None (no fits) 00 | | |  |
|  | **Counselling** |  | | |  |
| G17 | What kind of counselling was provided to you regarding the warning signs of High BP?  READ OUT | Risks related to High BP during pregnancy **Y/N**  Importance of Daily foetal movement count **Y/N**  Calcium + Vit D3 Supplementation **Y/N**  Early registration at Higher centre **Y/N**  Need for admission, monitoring and hospital delivery in case of emergency **Y/N**  Warning signs and symptoms during 3 months post-delivery **Y/N** | | |  |
| G18 | Were your family members counselled in case of fits due to high BP? | Yes 1  No 2  Don’t remember 77  None (no fits) 00 | | |  |
| G19 | General Counselling was provided by:  **(Multiple response question)** | ASHA A  ANM B  MO C  Specialist D  Others (specify) X  None Y | | |  |
|  | **Follow up** |  | | |  |
| G20a | **Ask IF yes coded in G13a**  Were you followed up after coming back from the referral facility? | Yes…………………………………………1  No………………………………………….2 | | | 🡪G21 |
| G20b | Who followed up?  **(Multiple response possible)** | ANM A  MO at PHC B  MO/Specialist at CHC C  MO/Specialist at Dist. Hosp or above D  Doctor at Private hospital E  Don’t remember Z  Other (specify) X | | |  |
| G21 | Was BP taken post-delivery? | Yes ……..………..………………………1  No ………………..………………………2  Don’t remember………………………..…77 | | |  |
| G22 | If you had any other complication post-delivery other than hypertehnsion? | YES…………..1  NO…………2 | | |  |
| G23 | Was the anti-hypertensive medication continued and reviewed after 1 wk., post-delivery at the nearest PHC? | Yes 1  No 2 | | |  |
| G24 | Were you Followed-up any time by ANM during three months of post-partum period? | Yes 1  No 2 | | | 🡪G27 |
| G25 | Did you Checked your BP at 6 monthsafter delivery? | Yes 1  No 2 | | |  |
| G26 | Did you Checked your BP at 12 monthsafter delivery? | Yes 1  No 2 | | |  |
| G27 | Were your hypertension problem resolved before delivery? | Yes 1  No 2 | | | 🡪G29 |
| G28 | If NO, what was done? | **Record verbatim** | | |  |
| G29 | What extent you were satisfied with the management / referral services you have received to address the hypertension problem? | Very Satisfied 5  Satisfied 4  Neither satisfied nor satisfied 3  Not satisfied 2  Not at all satisfied 1 | | |  |
|  | *Comments/Remarks If any* |  | | |  |

**Section I: Only for RDW with APH/Bleeding problem**

**(If recorded as having APH/bleeding problem in Open question (Section C)/MCP card/D15 question)**

**Definition- less than 24 weeks**

***Definition of APH: (Check USG)***

| ***No.*** | ***Questions*** | ***Codes*** | ***GO TO Q.*** |
| --- | --- | --- | --- |
|  | **Identification:** |  |  |
| I2 | Since you have reported bleeding issue, what type of bleeding did you observe during recent pregnancy? | Spotting 1  Mild 2  Heavy 3  Don’t remember 77 |  |
| I3 | Did you experience any pain? | Painful…………………………..1  Painless………………………….2 |  |
| I4 | In which month of pregnancy, did you observe bleeding? | Month of pregnancy : _________ |  |
| I5 | Type of APH identified? /MCP card  (If painless, minimal to moderate bleeding, was discussed after observing scan – May be Placenta Previa  If painful, with moderate to profuse bleeding, sudden onset – May be abruptio placenta  Preceded by labour pains and sudden cessation of pains, with minimal to moderate bleeding, revelation of foetal parts – May be Uterine rupture) | Placenta previa……………….1  Abruptio Placenta……………2  Uterine rupture ……………….3 |  |
| I6 | Where was APH diagnosed for first time in recent pregnancy? | Home 1  Subcentre 2  PHC 3  CHC 4  SDH/AH 5  DH 6  MC 7  MCH 8  Private hospital 9 |  |
| I7 | Who diagnosed APH for the first time in recent pregnancy? | ASHA……………..………………………1  ANM………………………………………2  MO………………………..………………3  Specialist …………………………………4  Others (specify)…………..………………99 |  |
| I8 | When scan was done, did they inform you about APH/Placenta previa? | Yes………….……………………………..1  No………………………………………….2  Don’t remember………….……………….77  None (Not Placenta previa) 00 |  |
| I9 | What additional history was taken when you have approached a health functionary with bleeding?  READ OUT | Duration, amount, onset of bleeding **Y/N**  Associated pain **Y/N**  Reduced or absent foetal movements **Y/N**  Previous caesarean section **Y/N**  Any documented USG **Y/N**  Other(Specify) Y/N |  |
|  | **Management:** |  |  |
| I10em | **Was there any emergency with APH?** | Yes 1  No 2 | 🡪I10a |
| I10 | In case of emergency, whether the following examinations were done?  READ OUT | Consciousness…….………………… **Y/N**  Pulse ………………………………… **Y/N**  B.P…………………………………… **Y/N**  Respiratory rate……………………… **Y/N**  Pallor ………………………………… **Y/N**  P/A - Uterine contour………………… **Y/N**  Foetal heart present or absent/rate…… **Y/N**  P/V- …………………………..……… **Y/N** Other(Specify) Y/N |  |
| I10A | At which facility your APH problem was Managed during your pregnancy with (CHILD NAME)?  **MULTIPLE RESPONSE POSSIBLE** | Home…………….…………….A  Subcentre……………..….…….B  PHC………………………....…C  CHC……………………………D  SDH/AH………………….……….…E  DH……………………………..F  MC……………..……….……..G  MCH……………..…………….H  Private hospital…………………I |  |
| I10B | Who managed APH?  **MULTIPLE RESPONSE POSSIBLE** | ANM A  MO at PHC B  Staff nurse at PHC C  MO/Specialist at CHC D  MO/Specialist at Dist. Hosp or above E  Private hospital F  Don’t remember Z  None Y  Other (specify) X |  |
| I10c | Did you require any blood transfusion? | Yes 1  No 2  Don’t remember 77 | 🡪I12  🡪I12 |
| I10D | If yes, How many units were received? | No. of units: ______ |  |
|  | **Referral** |  |  |
| I12 | Were you referred to any higher health facility? | Yes 1  No 2 | 🡪I16 |
| I13 | **ASK I13 AND 14 IF YES CODED IN I10em**  Was this an emergency referral? | Yes 1  No 2 |  |
| I14 | Before referring, in case of emergency, was the following management done?  READ OUT | Inserted I.V canula (16/18 G) Y/N  Taken sample for blood grouping and cross matching, haemoglobin, clotting time, bleeding time Y/N  Started i.v fluid (ringer lactate), 1L in 20mins Y/N  Inserted a Foleys catheter Y/N  Started Oxygen @ 6-8 litres by mask Y/N  Started blood transfusion if available Y/N  Spoke to higher facility for availability of bed Y/N  Other (specify) Y/N |  |
| I15a | Where were you referred?  **Codes**  Subcentre………………….1  PHC………………………...2  CHC…………………………3  SDH/AH………………….……….…4  DH……………………………..5  MC……………..……………..6  MCH……………..………….7  Private hospital…………8 | Referred From ____________________  Referred to_______________________  (CAPI NOTE: PLEASE CHECK THE CODE IN REFERRED TO SHOULD BE HIGHER THAN REFERRED FROM) |  |
| I15b | Which month were you referred during your pregnancy? | Month of pregnancy ______ |  |
| I15c | How long did it take for you to visit the above the referral facility? | Immediately/ same day 1  Next day 2  After 3-4 days 3  After a week 4  On emergency 5  Not visited the referred facility 0 |  |
| I15d | Were you referred to any other higher health facility after first referral? | Yes 1  No 2 | 🡪I16 |
| I15e | Where were you referred?  **Codes**  Subcentre………………….1  PHC………………………...2  CHC…………………………3  SDH/AH………………….……….…4  DH……………………………..5  MC……………..……………..6  MCH……………..………….7  Private hospital…………8 | Referred From ____________________  Referred to_______________________  (CAPI NOTE: PLEASE CHECK THE CODE IN REFERRED TO SHOULD BE HIGHER THAN REFERRED FROM) |  |
|  | **Counselling:** |  |  |
| I16 | **ASK IF 1 CODED IN I 5**  In case of placenta previa identified in the scan , what kind of counselling was provided?  READ OUT | To rest properly **Y/N**  Counsel the family about danger signs **Y/N**  Counsel about the Complications to mother and foetus **Y/N**  The need to shift to higher centre in case of emergency **Y/N**  Need of blood transfusions in case of emergency **Y/N** |  |
| I17 | **ASK IF YES IN I 13**  In case of emergency, what kind of counselling was provided? (For Abruptio Placenta and Uterine rupture)  READ OUT | Counsel the family about danger signs **Y/N**  Counsel the family about the Complications to mother and foetus **Y/N**  The urgent need to shift to higher centre **Y/N**  Need of blood transfusions **Y/N** |  |
| I18 | Overall counselling was provided by?  **(Multiple response question)** | ASHA A  ANM B  MO C  Specialist D  Others (specify) X  None Y |  |
|  | **Follow up:** |  |  |
| I19a | **ASK I19A AND I19 IF 1 CODED IN I12**  Were you followed up after coming back from the referral facility? | Yes…………………………………………1  No………………………………………….2 |  |
| I19 | Who followed up? | ANM 1  MO at PHC 2  MO/Specialist at CHC 3  MO/Specialist at Dist. Hosp or above 4  Doctor at Private hospital 5  Don’t remember 77  Other (specify) 99 |  |
| I20 | Are you having any of the following APH associated complications post delivery?  **(Multiple response question)** | PPH A  Blood transfusion B  ICU admission required for mother C  Other complications, (Specify) D  None Y |  |
| G27 | Were your APH problem resolved before delivery? | Yes…………………………………………1  No………………………………………….2 | 🡪G29 |
| G28 | If NO, what was done? |  |  |
| G29 | What extent you were satisfied with the management / referral services you have received to address the APH problem? | Very Satisfied 5  Satisfied 4  Neither satisfied nor satisfied 3  Not satisfied 2  Not at all satisfied 1 |  |
|  | *Comments/Remarks If any* |  |  |

**Section K: RDW with any other mentioned HRP on the MCP card/as recalled by RDW**

**(Administer this section to all respondents)**

| ***o.*** | ***Questions*** | ***Codes*** | ***GO TO Q.*** |
| --- | --- | --- | --- |
| K0 | Have you been identified with any other HRP | Yes 1  No 2 | 🡪 **SEC L** |
| KI | **Type of HRP** | GIVE CODE LIST FROM D15 (OTHER THAN, BP, ANAEMIA, APH) |  |
| K2 | **Was it identified before pregnancy** | Yes 1  No 2 |  |
| K3 | **If identified before at what age** | Women Age: ______ |  |
| K4 | What kind of History was taken? | RECORD VERBATIM |  |
| K5 | What kind of examination was done?  EG physical test such as eyes, hand, pulse etc | RECORD VERBATIM |  |
| K6 | What kind of investigations were performed? (Check in MCP Card) | RECORD VERBATIM |  |
| K7 | Who identified and registered you to be a case for the first time? | ANM A MO at PHC B Staff nurse at PHC C MO/Specialist at CHC D MO/Specialist at Dist. Hosp or above E Private hospital F Don’t remember Z none Y Other (specify) X |  |
| K8 | Where was it first identified? | Sub centre……………..….…….A PHC………………………....…B CHC……………………………C SDH/AH………………….……….…D DH……………………………..E MC……………..……….…….F MCH……………..…………….G Private hospital…………………H |  |
|  | **Management:** |  |  |
| K10 | Who managed you for the high risk during the pregnancy?  (**MULTIPLE RESPONSE)** | ANM A MO at PHC B Staff nurse at PHC C MO/Specialist at CHC D MO/Specialist at Dist. Hosp or above E Private hospital F Don’t remember Z None Y Other (specify) X |  |
| K11 | Where was your high risk condition was Managed? | Home…………….…………….A Subcentre……………..….…….B PHC………………………....…C CHC……………………………D SDH/AH………………….……….…E DH……………………………..F MC……………..……….……..G MCH……………..…………….H Private hospital…………………I |  |
|  | **Referral** |  |  |
| K12 | Were you referred to any higher health facility? | Yes 1  No 2 | 🡪 K16 |
| K13 | Was this an emergency referral? | Yes 1  No 2 |  |
| K14a | Where were you referred?  **Codes**  Subcentre 1  PHC 2  CHC 3  SDH/AH 4  DH 5  MC 6  MCH 7  Private hospital 8 | Referred From ____________________  Referred to_______________________  (CAPI NOTE: PLEASE CHECK THE CODE IN REFERRED TO SHOULD BE HIGHER THAN REFERRED FROM) |  |
| K14b | Which month were you referred during your pregnancy? | Month of pregnancy ______ |  |
| K14c | How long did it take for you to visit the above the referral facility? | Immediately/ same day 1  Next day 2  After 3-4 days 3  After a week 4  On emergency 5  Not visited the referred facility 0 | 🡪K15 |
| K14d | Were you referred to any other higher health facility after first referral? | Yes 1  No 2 | 🡪K15 |
| K14e | Where were you referred?  **Codes**  Subcentre………………….1  PHC………………………...2  CHC…………………………3  SDH/AH………………….……….…4  DH……………………………..5  MC……………..……………..6  MCH……………..………….7  Private hospital…………8 | Referred From ____________________  Referred to_______________________  (CAPI NOTE: PLEASE CHECK THE CODE IN REFERRED TO SHOULD BE HIGHER THAN REFERRED FROM) |  |
|  | **Follow up** |  |  |
| K15 | Were you followed up after coming back from the referral facility? | Yes 1  No 2 |  |
| K16 | Were your HRP problem resolved before delivery? | Yes 1  No 2 | 🡪K18 |
| K17 | If NO, what was done? |  |  |
| K18 | What extent you were satisfied with the management / referral services you have received to address the HRP issues? | Very Satisfied 5  Satisfied 4  Neither satisfied nor satisfied 3  Not satisfied 2  Not at all satisfied 1 |  |
|  | Comments |  |  |

**Section L: Client (RDW) Satisfaction Survey**

**(Who suffered from any high-risk pregnancy condition and sought care mainly in public facility)**

**Type of facility from where care for HRP was mainly sought:**

**HSC/ANM…1 ; PHC……….2 ; CHC………3 ; Area Hospital…..4**

**District hospital….5 ; Other public facility……..6**

**INSTRUCTIONS TO FILL THE FORMAT: PLEASE MENTION THE NUMBER IN THE SCORE BOX GIVEN BELOW STARTING FROM 1-5**

**1 - Poor, 2 – Average, 3 – Good, 4 – Very Good, 5 – Excellent**

| **L1** | | Attitude and Behaviour of Staff (Attitude and behaviour of the reception staff when you reached the facility, Adequacy of information displayed at the reception/ registration counter, Promptness of the registration process, Did somebody attend to you immediately? Time taken to be seen by the doctor after your arrival in the facility) | **Scale (1-5)** | **Comments/Remarks** |
| --- | --- | --- | --- | --- |
| **L2** | Cleanliness and Hygiene (General cleanliness of the building, corridors and premises, Cleanliness of OPD & immunization area, Cleanliness of ANC ward, Were bedsheets clean?, Availability of running water, hand-washing facilities in toilets, Availability of 24/7 safe and clean water supply) | **Scale (1-5)** | **Comments/Remarks** |  |
| **L3** | Comfort and Care (Satisfied with the safety, privacy and confidentiality in the OPD/ANC wards, Were regular, free meals provided to the mother during her stay in hospital? Were regular, free meals provided to the family during the mother’s stay in hospital? IF food is provided by the health facility, was the quality and quantity adequate?) | **Scale (1-5)** | **Comments/Remarks** |  |

| **S.no** | **Out of Pocket Expenses** |  | **Comments/Remarks** |
| --- | --- | --- | --- |
| L4 | Did you have to pay for any medications/any out of pocket expenses (If so, please specify) | Yes-1  No-2 | If yes how much:__________ |
| L5 | Did you have to pay for any diagnostic facilities (Laboratory, Radiology, Ultrasound, specialized investigation, etc.)? | Yes-1  No-2 | If yes how much:__________ |
| L6 | Did you have to pay for transport? | Yes-1  No-2 | If yes how much:__________ |

| **S.no** | **Quality of Service Delivery at the Facility (if stayed more than a day)** |  | **Comments/ Remarks** |
| --- | --- | --- | --- |
| L7 | Were you referred to external laboratories for tests? | Yes 1  No 2 |  |
| L8 | How many times was the patient seen by the doctor and nurses in a day? | A-Doctor_____  B-Nurse______ |  |
| L9 | Were you given any instructions at the time of discharge? | Yes 1  No 2 |  |
| L10a | Were the doctors and nurses friendly with you? | Yes 1 No 2 |  |
| L10b | Were the nurses friendly with you? | Yes 1 No 2 |  |
| **L11** | How satisfied are you with OVERALL MANAGEMENT you have received from the facility for the high risk pregnancy problem you have come to this facility ? | Poor 1  Average 2  Okay/Good 3  Very good 4  Excellent 5  Not applicable 9 |  |
| **L11a** | How was your overall satisfaction during your stay at the facility. | Poor 1  Average 2  Okay/Good 3  Very good 4  Excellent 5 |  |

**SECTION I** **: RESPONDENT (RDW’s) BACKGROUND**

***Interviewer: “Now I would like to ask some questions about you and your household.”***

| **Q. #** | **Question** | **Codes** | **Go to Q** |
| --- | --- | --- | --- |
| QQ1 | Which religion do you follow? | Hindu 1  Muslim 2  Christian 3  Jain 4  Buddhist 5  Sikh 6  No Religion 7  Other (specify)…………………….. 99 |  |
| QQ2 | What is your caste?  ***RECORD AS STATED BY RESPONDENT*** | Scheduled Caste (SC) 1  Scheduled Tribe (ST) 2  Other Backward Caste (OBC) 3  General 4  Other (Specify) 99 |  |
| 1. Q3 | How many persons usually live in your household including you  (who stayed here last night) | Number of members___________ |  |
| 1. Q4 | Did you ever attend a formal school? | Yes 1  No 2 | 🡪Q6 |
| 1. Q5 | What is the highest standard you have completed?  ***if not attended school or completed class one enter ‘00’***)  Till class 12th - Record the class that  respondent has passed last  Diploma/Technical/ITI 13  Graduation(BA/BSc/BCom/LLB) 14  Post-Graduation  (MA, MSc, M Com/LLM) 15  Professional Degree (MBA/MBBS) 16 | Highest standard completed…[ ___\| ___ ]  Less than 1 year completed or pre-primary...00  Don’t know ………………………………98 |  |
| 1. Q6 | What is your main occupation? | Household work 1  Not working/retired/unemployed 2  Cultivator 3  Agricultural labourer 4  Non-agricultural labourer(skilled) 5  Non-agricultural labourer(unskilled) 6  Business / petty trader/ self employed 7  Salaried employee government 8  Salaried employee private 9  Other (Specify) 99 |  |
| 1. Q7 | Did your husband ever attend a formal school? | Yes 1  No 2  No Husband……………..………………3 | 🡪Q9  🡪Q10 |
| 1. Q8 | What is the highest standard he has completed?  ***if not attended school or completed class one enter ‘00’***)  Till class 12th - Record the class that  respondent has passed last  Diploma/Technical/ITI 13  Graduation(BA/BSc/BCom/LLB) 14  Post-Graduation  (MA, MSc, MCom/LLM) 15  Professional Degree (MBA/MBBS) 16 | Highest standard completed…[ ___\| ___ ]  Less than 1 year completed or pre-primary..00  Don’t know ………………………………98 |  |
| 1. Q9 | What is your husband’s main occupation? | Household work 1  Not working/retired/unemployed 2  Cultivator 3  Agricultural labourer 4  Non-agricultural labourer(skilled) 5  Non-agricultural labourer(unskilled) 6  Business / petty trader/ self employed 7  Salaried employee government 8  Salaried employee private 9  Other (Specify) 99 |  |
| 1. Q10 | What is the main source of drinking water for members of your household? | Piped water(Dwelling/Yard/Plot) 1  Piped water (Public Tab) 2  Hand Pump into Dwelling/Yard/Plot 3  Public Hand Pump/Tube Well/Borehole 4  Protected Dug Well 5  Unprotected Dug Well 6  Rainwater 7  Surface water (river/dam/lake/pond/stream) 8  Other (Specify)___________________ 99 |  |
| 1. Q11 | What kind of TOILET facility do members of your household usually use? | OWN Flush facility 1  SHARE Flush 2  OWN Pit latrine 3  SHARE PIT LATRINE 4  No Facility/open space or field 5  Other (Specify)___________________ 99 |  |
| 1. Q12 | What type of fuel does your household mainly use for COOKING? | Electricity 1  LPG/Natural GAS 2  Biogas 3  Kerosene 4  Lignite 5  Charcoal 6  Wood 7  Straw/Shrubs/Grass 8  Dung Cakes 9  Other (Specify) 99 |  |
| 1. Q13 | Does any member of this household own any agricultural land? | Yes 1  No 2 | 🡪Q16 |
| 1. Q14 | How much agricultural land do members of this household own?  (IF NOT IN ACRES, THEN CONVERT INTO ACRES) | ACRES: ☐☐☐: ☐: |  |
| 1. Q15 | Out of above agricultural land How much land is irrigated?  (IF NOT IN ACRES, THEN CONVERT INTO ACRES) | ACRES: ☐☐☐: ☐  NONE……………………………….00  Don’t Know………………………98 |  |
| 1. Q16 | Does your household have a ration card? | Yes…………..…………………………..1  No……………………………………….2  Don’t know…………………………….98 | 🡪Q18  🡪Q18 |
| 1. Q17 | Which type of ration card does your household have?  (Yellow cards - below the poverty line (BPL)  White cards - above the poverty line (APL).  Red/orange cards - special scheme, the Antyodaya scheme) | White Card 1  Yellow card 2  Red/Orange card 3  Other (Specify) 99  Don’t know 98 |  |
| 1. Q18 | Do you (RDW) have your own mobile phone? | Yes-1  No- 2 | END |
| 1. Q19 | If RDW owns a mobile phone type of phone owned by RDW | Basic ….1  Smart…..2 |  |

**RECORD INTERVIEWER OBSERVATIONS**

**THANKS AND CLOSE**
