# Supplementary material for: Impact of a Multicomponent Intervention to Build Capacity of Public Health Workers to Make Algorithmic Diagnosis and Management of High-Risk Pregnancies in Uttar Pradesh, India: Protocol for a Matched-Control, Before-After, Quasi-Experimental Study With a Mixed Methods Design
Source: JMIR Res Protoc. 2025 Dec 9;14:e74993. doi: 10.2196/74993 (PMC12690279; doi:10.2196/74993)
Supplement: Multimedia Appendix 7 [file resprot-v14-e74993-s007.docx]

**Annexure 6a**

**Advancing Reduction in Mortality and morbidity of Mothers, Children and Neonates (ARMMAN)**

माताओं, बच्चों और नवजात शिशुओं की मृत्यु दर और रुग्णता में कमी लाना (एआरएमएएन)

**Integrated High-Risk Pregnancy Tracking & Management (IHRPTM)**

इंटीग्रेटेड उच्च जोखिम वाली गर्भावस्था ट्रैकिंग और प्रबंधन (आईएचआरपीटीएम)

**Informed consent for RDW (aged 18 years & above) participating in research**

रिसर्च में भाग लेने वाले आरडीडब्‍ल्‍यू (10 साल या अधिक उम्र के) के लिए सूचित सहमति

**Introduction:**

**परिचय:**

*Namaste*, my name is ______________________________, and I am working for ARMMAN, which in partnership with Government of Uttar Pradesh, is implementing a program known as ‘’Integrated High Risk Pregnancy Management program-IHRPTM. I am working for ____________ (Name of agency), responsible for collecting the data from eligible respondents. As a part of this program, ARMMAN is training and handholding all the ANMs, MOs and Gynaecologists in UP to efficiently track and manage six high-risk pregnancy (HRP) conditions. In this regard we wanted to conduct a survey with around 4,000 recently delivered women (RDW) to understand the services offered to women on ANC, identification, management, referral and follow up strategies adopted by ANMs/MOs while managing these HRPs. This survey will help us to understand management strategies, current burden of HRPs and your perceptions about the overall quality of ANC, diagnosis & management of three HRPs and satisfaction with HRP tracking, management, referral etc., services. as diagnosed by ANMs/MOs, so that an effective implementation strategy for ANMs/MOs can be developed and implemented for next 18 months.

नमस्ते, मेरा नाम ______________________________ है, और मैं अरमान के लिए काम कर रहा हूं, जो उत्तर प्रदेश सरकार के साथ साझेदारी में, ‘इंटीग्रेट हाई रिस्‍क प्रेग्‍नेंसी मैनेजमेंट प्रोग्राम-आईएचआरपीटीएम’ नाम के प्रोग्राम को क्रियान्वित कर रहा है। इस प्रोग्राम के एक हिस्से के रूप में, अरमान यूपी में सभी एएनएम, एमओ और गाइनकालजिस्ट को उच्च जोखिम वाली गर्भावस्था (एचआरपी) की छह समस्‍यों को कुशलतापूर्वक ट्रैक करने और प्रबंधित करने के लिए प्रशिक्षण और सहायता प्रदान कर रहा है।इस संबंध में हम हाल ही में बच्‍चे को जन्‍म देने वाली लगभग 4,000 महिलाओं (आरडीडब्ल्यू) के साथ एक सर्वे करना चाहते थे, ताकि एएनसी पर महिलाओं को दी जाने वाली सेवाओं, पहचान, प्रबंधन, रेफरल और एचआरपी का प्रबंधन करते समय एएनएम/एमओ द्वारा अपनाई गई फॉलो-अप रणनीतियों को समझा जा सके। यह सर्वे हमें प्रबंधन रणनीतियों, एचआरपी के वर्तमान बोझ और एएनसी की कुलमिलाकर गुणवत्ता, तीन एचआरपी के डायग्‍नोसिस और प्रबंधन और एएनएम/एमओ द्वारा डायग्‍नोसिस के अनुसार एचआरपी ट्रैकिंग, प्रबंधन, रेफरल आदि सेवाओं के साथ संतुष्टि के बारे में आपकी धारणाओं को समझने में मदद करेगा, ताकि एएनएम/एमओ के लिए एक प्रभावी कार्यान्वयन रणनीति तैयार की जा सके और अगले 18 महीनों के लिए लागू की जा सके।

**SELECTION PROCESS AND PURPOSE OF THE RESEARCH**

**चुनाव की प्रक्रिया और रिसर्च का उद्देश्‍य**

You are being asked to participate in this study because you satisfy our eligibility criteria which are: *‘’You were diagnosed as a ‘high risk case’ (severe/moderate anaemia, hypertension, ante-partum haemorrhage) or you were categorised as ‘normal’ without any of three high-risk pregnancy conditions – by ANM/MO during most recent pregnancy, in the past one year ’’*.

आपसे इस स्‍टडी में भाग लेने के लिए इसलिए कहा जा रहा है क्योंकि आप हमारी एलिजब‍िलिटी को क्राइटेरिया को पूरा करते हैं, जो हैं: ''आपको एक 'उच्च जोखिम वाले मामले' (गंभीर/मध्यम एनीमिया, हाइपरटेंशन, प्रसवपूर्व रक्तस्राव) के रूप में डायग्‍नोस किया गया था या आपको पिछले एक वर्ष में, सबसे हाल की गर्भावस्था के दौरान एएनएम/एमओ द्वारा उच्च जोखिम वाली गर्भावस्था संबंधी तीन समस्‍याओं के बिना 'सामान्य' के रूप में वर्गीकृत किया गया था।''

We will talk in private, in your home or any other location where you feel comfortable talking with us. I will read the questions to you and write down/record electronically your responses. No one else will be there when we talk.

हम आपके घर में, किसी अन्‍य स्‍थान पर, प्राइवेट में बात करेंगे जहां आप हमसे बात करने में सहज महसूस करती हो। मैं आपके लिए प्रश्‍नों को पढूंगा और आपके जवाब को इलेक्‍ट्रॉनिक तरीके से लिखूंगा/रिकॉर्ड करूंगा। जब हम बात करेंगे तो वहां पर कोई और नहीं होगा।

**STUDY PROCEDURES AND COMPENSATION**

**स्‍टडी की प्रक्रिया और क्षतिपूर्ति**

We will ask some general questions about you/your family, your recent ANC, HRPs during pregnancy, delivery care, immediate post-partum care. We will also ask you some questions about your knowledge, awareness, and experience of ANC care at facility and at home, and if you have developed three HRPs during ANC. Your participation in the study is voluntary and you may choose not to participate or to stop participating whenever you like, for any reason. We will not proceed without your consent. There are six sections in the survey and we think it will take about 50-60 minutes to complete the interview, but you can take as long as you need.

हम आप/आपके परिवार, आपकी हाल की एएनसी, गर्भावस्था के दौरान एचआरपी, प्रसव संबंधी देखभाल, प्रसव के तुरंत बाद की देखभाल के बारे में कुछ सामान्य प्रश्न पूछेंगे। हम आपसे सुविधा पर और घर पर एएनसी देखभाल के बारे में आपके ज्ञान, जागरूकता और अनुभव के बारे में भी कुछ प्रश्न पूछेंगे, और यह भी कि पूछेंगे कि क्या एएनसी के दौरान आपमें तीन एचआरपी विकसित हुए हैं। स्‍टडी में आपकी भागीदारी स्‍वैच्छिक है और आप भाग ना लेना या किसी भी कारण से, जब भी आप चाहे भागीदारी को रोकना चुन सकते हैं। हम आपकी सहमति के बिना आगे नहीं बढ़ेंगे। इस सर्वे में छह सेक्‍शन हैं और हमें लगता है कि इंटरव्‍यू को पूरा करने में 50-60 मिनट का समय लगेगा, लेकिन आप जितना समय चाहें ले सकती हैं।

**RISK, DISCOMFORT, AND BENEFIT**

**जोखिम, असुविधा और लाभ**

There are no major risks associated with your participation in this study, that your participation could become known to others outside the study. If you do not want to answer/respond to any questions that make you feel uncomfortable - we can simply move onto the next question. You can refuse to answer some questions or stop participating in the interview at any point during our interview. If you decline to participate or answer some questions, this decision will not impact your future access to health care or other services for you or your family. There are no benefits from your participation in this study. However, the findings from this research may help improve ANC care services in your district/UP. If you withdraw mid-stream, only with your permission data collected up to the point will be used, otherwise entire data collected from you will be deleted.

इस स्‍टडी में आपकी भागीदारी से जुड़े कोई बड़े जोखिम नहीं हैं, कि आपकी भागीदारी के बारे में स्‍टडी के बाहर अन्य लोगों को पता चल सकता है। यदि आप किसी ऐसे प्रश्न का जवाब/उत्‍तर नहीं देना चाहती हैं जो आपको असहज महसूस कराता है - तो हम अगले प्रश्न पर जा सकती हैं। आप हमारे इंटरव्‍यू के दौरान किसी भी समय कुछ सवालों का जवाब देने से मना कर सकती हैं या इंटरव्‍यू में भागीदारी रोक सकती हैं। यदि आप भाग लेने या कुछ सवालों का जवाब देने से इनकार करती हैं, तो यह निर्णय आपके या आपके परिवार के लिए स्वास्थ्य देखभाल या अन्य सेवाओं तक भविष्य की पहुँच को प्रभावित नहीं करेगा। इस स्‍टडी में आपकी भागीदारी से कोई लाभ नहीं है। हालाँकि, इस रिसर्च के निष्कर्ष आपके जिले/यूपी में एएनसी देखभाल सेवाओं को बेहतर बनाने में मदद कर सकते हैं। यदि आप बीच में ही इस स्‍टडी से बाहर निकल जाती हैं, तो उस समय तक एकत्र किए गए डेटा का इस्‍तेमाल केवल आपकी अनुमति से ही किया जाएगा, अन्यथा आपसे लिया किया गया पूरा डेटा डिलीट कर दिया जाएगा।

**CONFIDENTIALITY**

**गोपनीयता**

Your answers will not be shared with anyone outside this project. The information you provide will be kept strictly confidential; your and your baby’s names, and any personal and professional details will be removed from the interview at the time of analysis. During the discussion, I will be noting your response on a tablet. However, the noted information will be securely stored and will not be shared with anyone outside the research team. We will destroy personal information once we complete the study.

आपके जवाबो को इस प्रोजेक्‍ट के बाहर किसी से साथ साझा नहीं किया जाएगा। आपके द्वारा दी गई जानकारी को पूरी तरह से गोपनीय रखा जाएगा, आपके और आपके बच्‍चे के नाम, और किसी भी व्‍यक्तिगत या प्रोफेशनल जानकारी को विश्‍लेषण के समय इंटरव्‍यू से हटा दिया जाएगा। इस चर्चा के दौरान, मैं आपके जवाबों को टैबलेट पर नोट करूंगा। हांलाकि, हालांकि नोट की गई जानकारी को सुरक्षित तरीके से स्‍टोर किया जाएगा और रिसर्च टीम के बाहर किसी के साथ साझा नहीं किया जाएगा। हम स्‍टडी को पूरा करने के बाद आपकी व्‍यक्तिगत जानकारी को नष्‍ट कर देंगे।

**Compensation:**

क्षतिपूति**:**

We will not pay you or your family for taking part in the study.

हम स्‍टडी में भाग लेने के लिए आपको या आपके परिवार को कोई भुगतान नहीं करेंगे।

**Voluntary Participation:**

स्‍वैच्छिक भागीदारी:

Taking part in this study is voluntary, meaning you decide if you want to take part in the study. There will be no consequence to you or to your family if you choose not to take part or if you want to stop taking part at any time. If you decide not to take part or stop at any time, we will respect it and not ask you why you do not want to take part.

इस स्‍टडी में भाग लेना स्वैच्छिक है, यानि कि आप तय करती हैं कि क्‍या आप स्‍टडी में भाग लेना चाहती हैं या नहीं। आप या आपके परिवार पर इसका कोई असर नहीं होगा, और यदि आप भाग नहीं लेना चुनती हैं या किसी भी समय भागीदारी को समाप्‍त करना चाहती हैं तो इसके लिए कोई पैनल्‍टी नहीं है। यदि आप किसी भी समय भाग नहीं लेने या भागीदारी समाप्‍त करने का निर्णय लेती हैं, तो हम इसका सम्मान करेंगे और आपसे यह नहीं पूछेंगे कि आप भाग क्यों नहीं लेना चाहती हैं।

**Contact information:** This study is being conducted by ARMMAN in partnership with Government of UP, and the study protocol has been reviewed by Sigma ethics committee, that works to protect your rights and welfare. If you have additional questions or concerns or in the case of an emergency, please contact:

**संपर्क की जानकारी:** यह स्‍टडी को अरमान द्वारा उत्तर प्रदेश सरकार के साथ साझेदारी में किया जा रहा है, और स्‍टडी प्रोटोकॉल की समीक्षा सिग्मा इथिकल कमेटी द्वारा की गई है, जो आपके अधिकारों और कल्याण की रक्षा के लिए काम करती है। यदि आपके पास कोई और प्रश्न या चिंताएँ हैं या किसी आपात स्थिति में, कृपया संपर्क करें:

Name: Dr Hanimi Reddy (Principal investigator)

Mobile: 9911822445

Email: [hanimi@armman.org](mailto:hanimi@armman.org)

नाम: डॉ. हनीमी रेड्डी (प्रमुख इन्‍वेटीगेटर)

मोबाइल: 9911822445

ईमेल: hanimi@armman.org

If you have any concerns regarding your rights as a participant, please contact Sigma Research and Consulting (IRB) at [irb.sigma@sigma-india.in](mailto:irb.sigma@sigma-india.in), Phone: 011- 41063450

**CONSENT**

**सहमति**

If you are not able to understand any question/topic during the interview, please feel free to ask me to repeat. The information we are collecting is important for the improvement of health programs for mothers and new-borns in India, so we ask that you answer the questions to the best of your knowledge and ability.

यदि आप इंटरव्‍यू के दौरान कोई प्रश्‍न/विषय को नहीं समझ पा रही हैं, तो कृपया मुझसे बेझिझक पूछें। हम जो जानकारी एकत्र कर रहे हैं वह भारत में माताओं और नवजात शिशुओं के लिए हेल्‍थ प्रोग्राम में सुधार करने के लिए महत्वपूर्ण है, इसलिए हम आपसे अनुरोध करते हैं कि आप अपने सर्वोत्तम ज्ञान और क्षमता के अनुसार सवालों के जवाब दें।

Now, can you tell me if you **agree** to participate in this survey?

अब, क्या आप मुझे बता सकती हैं कि क्या आप इस सर्वे में भाग लेने के लिए सहमत हैं? यदि आप हाँ कहती हैं, तो इसका मतलब है कि आप स्‍टडी का हिस्सा बनने के लिए सहमत हैं।

**Participant’s signature_______________________________________ Date: ___________**

**प्रतिभागी के हस्‍ताक्षर तारीख**

***If respondent cannot provide written consent:***

***यदि रिस्‍पोंडेंट लिखित सहमति नहीं प्रदान कर सकता है:***

Do you have any questions? YES NO

क्‍या आपके पास कोई प्रश्‍न हैं हां नहीं

Do you consent to participate in this study? YES NO

क्‍या आप इस स्‍टडी में भाग लेने के लिए सहमति देती हैं? हां नहीं

Do you consent for spot check by supervisor/research staff of ARRMAN, to cross-check your responses to a sample of questions? YES NO (if applicable)

Please let me know if you would like to keep a copy of this form so that you can review the information at a later date, contact someone about the study, or keep it for your records.

कृपया मुझे बताएं कि क्या आप इस फॉर्म की एक कॉपी रखना चाहेंगी ताकि आप बाद में इसकी जानकारी को देख सकें, स्‍टडी के बारे में किसी से संपर्क कर सकें, या इसे अपने रिकॉर्ड के लिए रख सकें।

May we begin the interview?: Yes🡪 Start interview No🡪 End interview

क्‍या हम इंटरव्‍यू शुरू कर सकते हैं?: हां🡪 इंटरव्‍यू शुरू करें नहीं🡪 इंटरव्‍यू समाप्‍त करें

**Annexure 6bपरिशिष्‍ट 6b**

**Advancing Reduction in Mortality and morbidity of Mothers, Children and Neonates (ARMMAN)**

माताओं, बच्चों और नवजात शिशुओं की मृत्यु दर और रुग्णता में कमी लाना (एआरएमएएन)

**Integrated High-Risk Pregnancy Tracking & Management (IHRPTM)**

**इंटीग्रेटेड उच्च जोखिम वाली गर्भावस्था ट्रैकिंग और प्रबंधन (आईएचआरपीटीएम)**

**Parent/Guardian Consent Form for Participation of Daughter/Daughter-in-law below 18 years in the research**

रिसर्च में 18 साल से कम आयु की आरडीडब्‍ल्‍यू की भागीदारी के लिए माता-पिता/अभिभावक की सहमति

**Introduction:**

**परिचय:**

*Namaste*, my name is ______________________________, and I am working for ARMMAN, which in partnership with Government of Uttar Pradesh, is implementing a program known as ‘’Integrated High Risk Pregnancy Management program-IHRPTM. As a part of this program, I am working for ____________ (Name of agency), responsible for collecting the data from eligible respondents. ARMMAN is training and handholding all the ANMs, MOs and Gynaecologists in UP to efficiently track and manage six high-risk pregnancy (HRP) conditions. In this regard we wanted to conduct a survey with around 4,000 recently delivered women (RDW) to understand the services offered to women on ANC, identification, management, referral and follow up strategies adopted by ANMs/MOs while managing these HRPs. This survey will help us to understand management strategies, current burden of HRPs and your perceptions about the overall quality of ANC, diagnosis & management of three HRPs and satisfaction with HRP tracking, management, referral etc., services. as diagnosed by ANMs/MOs, so that an effective implementation strategy for ANMs/MOs can be developed and implemented for next 18 months.

नमस्ते, मेरा नाम ______________________________ है, और मैं अरमान के लिए काम कर रहा हूं, जो उत्तर प्रदेश सरकार के साथ साझेदारी में, ‘इंटीग्रेट हाई रिस्‍क प्रेग्‍नेंसी मैनेजमेंट प्रोग्राम-आईएचआरपीटीएम’ नाम के प्रोग्राम को क्रियान्वित कर रहा है। इस प्रोग्राम के एक हिस्से के रूप में, अरमान यूपी में सभी एएनएम, एमओ और गाइनकालजिस्ट को उच्च जोखिम वाली गर्भावस्था (एचआरपी) की छह समस्‍यों को कुशलतापूर्वक ट्रैक करने और प्रबंधित करने के लिए प्रशिक्षण और सहायता प्रदान कर रहा है।इस संबंध में हम हाल ही में बच्‍चे को जन्‍म देने वाली लगभग 4,000 महिलाओं (आरडीडब्ल्यू) के साथ एक सर्वे करना चाहते थे, ताकि एएनसी पर महिलाओं को दी जाने वाली सेवाओं, पहचान, प्रबंधन, रेफरल और एचआरपी का प्रबंधन करते समय एएनएम/एमओ द्वारा अपनाई गई फॉलो-अप रणनीतियों को समझा जा सके। यह सर्वे हमें प्रबंधन रणनीतियों, एचआरपी के वर्तमान बोझ और एएनसी की कुलमिलाकर गुणवत्ता, तीन एचआरपी के डायग्‍नोसिस और प्रबंधन और एएनएम/एमओ द्वारा डायग्‍नोसिस के अनुसार एचआरपी ट्रैकिंग, प्रबंधन, रेफरल आदि सेवाओं के साथ संतुष्टि के बारे में आपकी धारणाओं को समझने में मदद करेगा, ताकि एएनएम/एमओ के लिए एक प्रभावी कार्यान्वयन रणनीति तैयार की जा सके और अगले 18 महीनों के लिए लागू की जा सके।

**SELECTION PROCESS AND PURPOSE OF THE RESEARCH**

**चुनाव की प्रक्रिया और रिसर्च का उद्देश्‍य**

You are being asked to participate in this study because your daughter/daughter-in-law satisfy our eligibility criteria which are: *‘’She was diagnosed as a ‘high risk case’ (severe/moderate anaemia, hypertension, ante-partum haemorrhage) or she was categorised as ‘normal’ without any of three high-risk pregnancy conditions – by ANM/MO during most recent pregnancy, in the past one year ’’*.

आपसे इस स्‍टडी में भाग लेने के लिए इसलिए कहा जा रहा है क्योंकि आपकी बेटी/बहू/पत्‍नी एलिजबिलिटी के क्राइटेरिया को पूरा करती हैं, जो हैं: ''उसे एक 'उच्च जोखिम वाले मामले' (गंभीर/मध्यम एनीमिया, हाइपरटेंशन, प्रसवपूर्व रक्तस्राव) के रूप में डायग्‍नोस किया गया था या उसे पिछले एक वर्ष में, सबसे हाल की गर्भावस्था के दौरान एएनएम/एमओ द्वारा उच्च जोखिम वाली गर्भावस्था संबंधी तीन समस्‍याओं के बिना 'सामान्य' के रूप में वर्गीकृत किया गया था।''

We will talk with your daughter/daughter-in-law in private, in your home or any other location where she feels comfortable talking with us. A female investigator will read the questions to your daughter/daughter-in-law and write down/record electronically your responses. No one else will be there when we talk. There are six sections in the survey and we think it will take about 50-60 minutes to complete the interview, but you can take as long as you need.

हम आपकी बेटी/बहू/पत्‍नी से आपके घर में, किसी अन्‍य स्‍थान पर, प्राइवेट में बात करेंगे जहां वह हमसे बात करने में सहज महसूस करती हो। एक महिला इन्‍वेस्‍टीगेटर आपकी बेटी/बहू/पत्‍नी के लिए प्रश्‍नों को पढेगी और आपके जवाब को इलेक्‍ट्रॉनिक तरीके से लिखेगी/रिकॉर्ड करेगी। जब हम बात करेंगे तो वहां पर कोई और नहीं होगा। इस सर्वे में छह सेक्‍शन हैं और हमें लगता है कि इंटरव्‍यू को पूरा करने में 50-60 मिनट का समय लगेगा, लेकिन आप जितना समय चाहें ले सकती हैं।

**STUDY PROCEDURES AND COMPENSATION**

**स्‍टडी की प्रक्रिया और क्षतिपूर्ति**

We will ask some general questions about you/your family, your recent ANC, HRPs during pregnancy, delivery care, immediate post-partum care. We will also ask you some questions about your knowledge, awareness, and experience of ANC care at facility and at home, and if you have developed three HRPs during ANC. Your participation in the study is voluntary and you may choose not to participate or to stop participating whenever you like, for any reason. We will not proceed without your consent.

हम आप/आपके परिवार, आपकी हाल की एएनसी, गर्भावस्था के दौरान एचआरपी, प्रसव संबंधी देखभाल, प्रसव के तुरंत बाद की देखभाल के बारे में कुछ सामान्य प्रश्न पूछेंगे। हम आपसे सुविधा पर और घर पर एएनसी देखभाल के बारे में आपके ज्ञान, जागरूकता और अनुभव के बारे में भी कुछ प्रश्न पूछेंगे, और यह भी कि पूछेंगे कि क्या एएनसी के दौरान आपमें तीन एचआरपी विकसित हुए हैं। स्‍टडी में आपकी भागीदारी स्‍वैच्छिक है और आप भाग ना लेना या किसी भी कारण से, जब भी आप चाहे भागीदारी को रोकना चुन सकते हैं।

**RISK, DISCOMFORT, AND BENEFIT**

**जोखिम, असुविधा और लाभ**

There are no major risks associated with your participation in this study, that your daughter/daughter-in-law participation could become known to others outside the study. To try and make it so this will not happen we will not write down or record your / your (daughter/daughter-in-law)’s name or other information that could identify her when we write down her responses**.** If someone comes near us to find out what we are talking about we will stop talking until they leave. If you or your daughter/daughter-in-law is upset by or does not like any of the questions we ask you or she does not have to answer the questions and you or she can stop taking part in the study at any time. We will also provide your daughter/daughter-in-law with contact information for people who provide MCH services for RDW who they can talk with if they are upset if they want to.

इस स्‍टडी में आपकी भागीदारी से जुड़े कोई बड़े जोखिम नहीं हैं, कि आपकी बेटी/बहू/पत्‍नी भागीदारी के बारे में स्‍टडी के बाहर अन्य लोगों को पता चल सकता है। ऐसा न हो, इसके लिए हम जब आपकी/आपकी बेटी/बहू/पत्नी जवाब लिखेंगे तो उसका नाम या अन्य जानकारी नहीं लिखेंगे या रिकॉर्ड नहीं करेंगे ताकि उसकी पहचान हो सके। अगर यह जानने के लिए कोई हमारे पास आता है कि हम किस बारे में बात कर रहे हैं, तो हम तब तक बात करना बंद कर देंगे तब तक दोबारा शुरू नहीं करेंगे जब तक वे चले नहीं जाते। यदि आप या आपकी बेटी/बहू/पत्नी हमारे द्वारा पूछे गए किसी सवाल से परेशान होती हैं या जो हम पूछ रहे हैं वह उसे पसंद नहीं आता है तो उसे सवालों का जवाब देने की आवश्यकता नहीं है और आप या वह किसी भी समय स्‍टडी में भाग लेना बंद कर सकती हैं। हम आपकी बेटी/बहू/पत्नी को उन लोगों की संपर्क जानकारी भी देंगे जो आरडीडब्लू के लिए एमसीएच सेवाएं प्रदान करते हैं, ताकि यदि वे परेशान हों तो वे उनसे बात कर सकें।

If you or your daughter/daughter-in-law do not want to answer/respond to any questions that make you/respondent feel uncomfortable - we can simply move onto the next question. You / respondent can refuse to answer some questions or stop participating in the interview at any point during our interview. If you/respondent decline to participate or answer some questions, this decision will not impact your/respondents future access to health care or other services for you or your family.

यदि आप या आपकी बेटी/बहू/पत्‍नी किसी ऐसे प्रश्न का जवाब/उत्‍तर नहीं देना चाहती हैं जो आपको/रिस्‍पोंडेंट को असहज महसूस कराता है- तो हम अगले प्रश्न पर जा सकती हैं। आप/रिस्‍पोंडेंट इंटरव्‍यू के दौरान किसी भी समय कुछ सवालों का जवाब देने से मना कर सकती हैं या इंटरव्‍यू में भागीदारी रोक सकती हैं। यदि आप/रिस्‍पोंडेंट भाग लेने या कुछ सवालों का जवाब देने से इनकार करती हैं, तो यह निर्णय आपके या आपके परिवार के लिए स्वास्थ्य देखभाल या अन्य सेवाओं तक भविष्य की पहुँच को प्रभावित नहीं करेगा।

There are no benefits from your daughter/daughter-in-law participation in this study. However, the findings from this research may help improve ANC care services in your district/UP. If you or respondent withdraw mid-stream, only with your daughter/daughter-in-law permission data collected up to the point will be used, otherwise entire data collected from daughter/daughter-in-law will be deleted.

इस स्‍टडी में आपकी बेटी/बहू/पत्‍नी की भागीदारी से कोई लाभ नहीं है। हालाँकि, इस रिसर्च के निष्कर्ष आपके जिले/यूपी में एएनसी देखभाल सेवाओं को बेहतर बनाने में मदद कर सकते हैं। यदि आप/रिस्‍पोंडेंट बीच में ही इस स्‍टडी से बाहर निकल जाती हैं, तो उस समय तक एकत्र किए गए डेटा का इस्‍तेमाल केवल आपकी बेटी/बहू/पत्‍नी की अनुमति से ही किया जाएगा, अन्यथा आपकी बेटी/बहू/पत्‍नी लिया किया गया पूरा डेटा डिलीट कर दिया जाएगा।

**CONFIDENTIALITY**

**गोपनीयता**

Your daughter/daughter-in-law answers will not be shared with anyone outside this project. The information your daughter/daughter-in-law provide will be kept strictly confidential; your and your daughter/daughter-in-law/wife names, and any personal and professional details will be removed from the interview at the time of analysis. During the discussion, we will be noting your response on a tablet. However, the noted information will be securely stored and will not be shared with anyone outside the research team. We will destroy personal information once we complete the study. We will not tell anybody including your family members and other members of the household what your daughter/daughter-in-law tell us. We will not identify you in anything we write about the study. We will not tell you what your daughter/daughter-in-law tells us.

आपकी बेटी/बहू/पत्‍नी के जवाबो को इस प्रोजेक्‍ट के बाहर किसी से साथ साझा नहीं किया जाएगा। आपकी बेटी/बहू/पत्‍नी द्वारा दी गई जानकारी को पूरी तरह से गोपनीय रखा जाएगा, आपका और आपकी बेटी/बहू/पत्‍नी के नाम, और किसी भी व्‍यक्तिगत या प्रोफेशनल जानकारी को विश्‍लेषण के समय इंटरव्‍यू से हटा दिया जाएगा। इस चर्चा के दौरान, मैं आपके जवाबों को टैबलेट पर नोट करूंगा। हांलाकि, हालांकि नोट की गई जानकारी को सुरक्षित तरीके से स्‍टोर किया जाएगा और रिसर्च टीम के बाहर किसी के साथ साझा नहीं किया जाएगा। हम स्‍टडी को पूरा करने के बाद आपकी व्‍यक्तिगत जानकारी को नष्‍ट कर देंगे। हम आपके परिवार के सदस्यों और घर के अन्य सदस्यों सहित किसी को भी यह नहीं बताएंगे कि आपकी बेटी/बहू/पत्नी हमें क्या बताती है। स्‍टडी के बारे में हम जो कुछ भी लिखेंगे, उसमें हम आपकी पहचान नहीं बताएंगे। हम आपको नहीं बताएंगे कि आपकी बेटी/बहू/पत्नी हमें बताती है।

**Compensation:**

क्षतिपूति**:**

We will not pay you or your daughter/daughter-in-law for taking part in the study.

हम स्‍टडी में भाग लेने के लिए आपको या आपके बेटी/बहू/पत्‍नी को कोई भुगतान नहीं करेंगे।

**Voluntary Participation:**

स्‍वैच्छिक भागीदारी:

Taking part in this study is voluntary, meaning you decide if you want to take part in the study and your daughter/daughter-in-law/wife may be in the study, and your daughter/daughter-in-law decides to take part in the study. Even though you give permission, your daughter/daughter-in-law does not have to be in the study if she does not want to. There will be no consequence to your daughter/daughter-in-law or to your family if you choose not to take part or do not let your daughter/daughter-in-law take part. if you or your daughter/daughter-in-law/wife decides not to take part or stop at any time, we will respect this and not ask you why your daughter/daughter-in-law do not want to take part.

इस स्‍टडी में भाग लेना स्वैच्छिक है, यानि कि आप तय करती हैं कि क्‍या आप स्‍टडी में भाग लेना चाहती हैं या नहीं या आपकी बेटी/बहू/पत्‍नी स्‍टडी में भाग लेगी या नहीं और स्‍टडी में भाग लेने के बारे में आपकी बेटी/बहू/पत्‍नी तय करती है। भले ही आप अनुमति देती हैं, फिर भी यदि आपकी बेटी/बहू/पत्‍नी स्‍टडी में भाग नहीं लेना चाहती है तो वह भाग नहीं लेगी। इसका आपकी बेटी/बहू/पत्‍नी या आपके परिवार पर कोई असर नहीं होगा, और यदि आप भाग नहीं लेना चुनती हैं या आपकी बेटी/बहू/पत्‍नी को भाग नहीं लेने देना चाहती हैं इसके लिए कोई दंड नहीं है। यदि आप या आपकी बेटी/बहू/पत्नी किसी भी समय भाग न लेने या भागीदारी को समापत करने का निर्णय लेते हैं, तो हम इसका सम्मान करेंगे तथा आपसे यह नहीं पूछेंगे कि आपकी बेटी/बहू/पत्नी भाग क्यों नहीं लेना चाहती हैं।

**Contact information:** This study is being conducted by ARMMAN in partnership with Government of UP, and the study protocol has been reviewed by Sigma ethics committee, that works to protect your rights and welfare. If you have additional questions or concerns or in the case of an emergency, please contact:

**संपर्क की जानकारी:** यह स्‍टडी को अरमान द्वारा उत्तर प्रदेश सरकार के साथ साझेदारी में किया जा रहा है, और स्‍टडी प्रोटोकॉल की समीक्षा सिग्मा इथिकल कमेटी द्वारा की गई है, जो आपके अधिकारों और कल्याण की रक्षा के लिए काम करती है। यदि आपके पास कोई और प्रश्न या चिंताएँ हैं या किसी आपात स्थिति में, कृपया संपर्क करें:

Name: Dr Hanimi Reddy (Principal investigator)

Mobile: 9911822445

Email: [hanimi@armman.org](mailto:hanimi@armman.org)

नाम: डॉ. हनीमी रेड्डी (प्रमुख इन्‍वेटीगेटर)

मोबाइल: 9911822445

ईमेल: hanimi@armman.org

If you have any concerns regarding your rights as a participant, please contact Sigma Research and Consulting (IRB) at [irb.sigma@sigma-india.in](mailto:irb.sigma@sigma-india.in), Phone: 011- 41063450

**CONSENT**

**सहमति**

. Now, can you tell me if you agree to participate in this survey. If you say yes, it means that you have agreed about participation of your daughter/daughter-in-law. We will interview your daughter/daughter-in-law only after obtaining assent from her.

यदि आप इंटरव्‍यू के दौरान कोई प्रश्‍न/विषय को नहीं समझ पा रही हैं, तो कृपया मुझसे बेझिझक पूछें। हम जो जानकारी एकत्र कर रहे हैं वह भारत में माताओं और नवजात शिशुओं के लिए हेल्‍थ प्रोग्राम में सुधार करने के लिए महत्वपूर्ण है, इसलिए हम आपसे अनुरोध करते हैं कि आप अपने सर्वोत्तम ज्ञान और क्षमता के अनुसार सवालों के जवाब दें।

We seek your consent to talk to your daughter/daughter-in-law for this study. We will also take assent confirmation from your daughter-in-law before we talk to her. Now, can you tell me if you **agree** to participate in this survey? अब, क्या आप मुझे बता सकती हैं कि क्या आप इस सर्वे में भाग लेने के लिए सहमत हैं? यदि आप हाँ कहती हैं, तो इसका मतलब है कि आप स्‍टडी का हिस्सा बनने के लिए सहमत हैं।

**Adult member/guardian signature_______________________________________ Date: ___________**

**वयस्‍क सदस्‍य/अभिभावक के हस्‍ताक्षर तारीख**

***If respondent cannot provide written consent:***

***यदि रिस्‍पोंडेंट लिखित सहमति नहीं प्रदान कर सकता है:***

Do you have any questions? YES NO

क्‍या आपके पास कोई प्रश्‍न हैं हां नहीं

Do you consent to participate in this study? YES NO

क्‍या आप इस स्‍टडी में भाग लेने के लिए सहमति देती हैं? हां नहीं

Do you give consent for your daughter/daughter-in-law to participate in this study? YES NO

आप आप अपनी बेटी/बहू/पत्‍नी को इस स्‍टडी में भाग के लिए सहमति देती हैं? हां नहीं

Please let me know if you would like to keep a copy of this form so that you can review the information at a later date, contact someone about the study, or keep it for your records.

कृपया मुझे बताएं कि क्या आप इस फॉर्म की एक कॉपी रखना चाहेंगी ताकि आप बाद में इसकी जानकारी को देख सकें, स्‍टडी के बारे में किसी से संपर्क कर सकें, या इसे अपने रिकॉर्ड के लिए रख सकें।

May we begin the interview?: Yes🡪 Start interview No🡪 End interview

क्‍या हम इंटरव्‍यू शुरू कर सकते हैं?: हां🡪 इंटरव्‍यू शुरू करें नहीं🡪 इंटरव्‍यू समाप्‍त करें

**Annexure 6c.**

**Assent Form for Daughter/daughter-in-law below 18 years for participating in Research**

परिशिष्‍ट 6c. रिसर्च में भागीदारी के लिए 18 साल से कम की आरडीडब्‍लयू के लिए सहमति फॉर्म

**Introduction:**

**परिचय:**

*Namaste*, my name is ______________________________, and I am working for ARMMAN, which in partnership with Government of Uttar Pradesh, is implementing a program known as ‘’Integrated High Risk Pregnancy Management program-IHRPTM. I am working for ____________ (Name of agency), responsible for collecting the data from eligible respondents. As a part of this program, ARMMAN is training and handholding all the ANMs, MOs and Gynaecologists in UP to efficiently track and manage six high-risk pregnancy (HRP) conditions. In this regard we wanted to conduct a survey with around 4,000 recently delivered women (RDW) to understand the services offered to women on ANC, identification, management, referral and follow up strategies adopted by ANMs/MOs while managing these HRPs. This survey will help us to understand management strategies, current burden of HRPs and your perceptions about the overall quality of ANC, diagnosis & management of three HRPs and satisfaction with HRP tracking, management, referral etc., services. as diagnosed by ANMs/MOs, so that an effective implementation strategy for ANMs/MOs can be developed and implemented for next 18 months.

नमस्ते, मेरा नाम ______________________________ है, और मैं अरमान के लिए काम कर रहा हूं, जो उत्तर प्रदेश सरकार के साथ साझेदारी में, ‘इंटीग्रेट हाई रिस्‍क प्रेग्‍नेंसी मैनेजमेंट प्रोग्राम-आईएचआरपीटीएम’ नाम के प्रोग्राम को क्रियान्वित कर रहा है। इस प्रोग्राम के एक हिस्से के रूप में, अरमान यूपी में सभी एएनएम, एमओ और गाइनकालजिस्ट को उच्च जोखिम वाली गर्भावस्था (एचआरपी) की छह समस्‍यों को कुशलतापूर्वक ट्रैक करने और प्रबंधित करने के लिए प्रशिक्षण और सहायता प्रदान कर रहा है।इस संबंध में हम हाल ही में बच्‍चे को जन्‍म देने वाली लगभग 4,000 महिलाओं (आरडीडब्ल्यू) के साथ एक सर्वे करना चाहते थे, ताकि एएनसी पर महिलाओं को दी जाने वाली सेवाओं, पहचान, प्रबंधन, रेफरल और एचआरपी का प्रबंधन करते समय एएनएम/एमओ द्वारा अपनाई गई फॉलो-अप रणनीतियों को समझा जा सके। यह सर्वे हमें प्रबंधन रणनीतियों, एचआरपी के वर्तमान बोझ और एएनसी की कुलमिलाकर गुणवत्ता, तीन एचआरपी के डायग्‍नोसिस और प्रबंधन और एएनएम/एमओ द्वारा डायग्‍नोसिस के अनुसार एचआरपी ट्रैकिंग, प्रबंधन, रेफरल आदि सेवाओं के साथ संतुष्टि के बारे में आपकी धारणाओं को समझने में मदद करेगा, ताकि एएनएम/एमओ के लिए एक प्रभावी कार्यान्वयन रणनीति तैयार की जा सके और अगले 18 महीनों के लिए लागू की जा सके।

**SELECTION PROCESS AND PURPOSE OF THE RESEARCH**

**चुनाव की प्रक्रिया और रिसर्च का उद्देश्‍य**

You are being asked to participate in this study because you satisfy our eligibility criteria which are: *‘’You were diagnosed as a ‘high risk case’ (severe/moderate anaemia, hypertension, ante-partum haemorrhage) or you were categorised as ‘normal’ without any of three high-risk pregnancy conditions – by ANM/MO during most recent pregnancy, in the past one year ’’*.

आपसे इस स्‍टडी में भाग लेने के लिए इसलिए कहा जा रहा है क्योंकि आप हमारी एलिजबिलिटी को क्राइटेरिया को पूरा करते हैं, जो हैं: ''आपको एक 'उच्च जोखिम वाले मामले' (गंभीर/मध्यम एनीमिया, हाइपरटेंशन, प्रसवपूर्व रक्तस्राव) के रूप में डायग्‍नोस किया गया था या आपको पिछले एक वर्ष में, सबसे हाल की गर्भावस्था के दौरान एएनएम/एमओ द्वारा उच्च जोखिम वाली गर्भावस्था संबंधी तीन समस्‍याओं के बिना 'सामान्य' के रूप में वर्गीकृत किया गया था।''

We will talk in private, in your home or any other location where you feel comfortable talking with us. I will read the questions to you and write down/record electronically your responses. No one else will be there when we talk.

हम आपके घर में, किसी अन्‍य स्‍थान पर, प्राइवेट में बात करेंगे जहां आप हमसे बात करने में सहज महसूस करती हो। मैं आपके लिए प्रश्‍नों को पढूंगा और आपके जवाब को इलेक्‍ट्रॉनिक तरीके से लिखूंगा/रिकॉर्ड करूंगा। जब हम बात करेंगे तो वहां पर कोई और नहीं होगा।

**STUDY PROCEDURES AND COMPENSATION**

**स्‍टडी की प्रक्रिया और क्षतिपूर्ति**

We will ask some general questions about you/your family, your recent ANC, HRPs during pregnancy, delivery care, immediate post-partum care. We will also ask you some questions about your knowledge, awareness, and experience of ANC care at facility and at home, and if you have developed three HRPs during ANC. Your participation in the study is voluntary and you may choose not to participate or to stop participating whenever you like, for any reason. We will not proceed without your consent. There are six sections in the survey and we think it will take about 50-60 minutes to complete the interview, but you can take as long as you need.

हम आप/आपके परिवार, आपकी हाल की एएनसी, गर्भावस्था के दौरान एचआरपी, प्रसव संबंधी देखभाल, प्रसव के तुरंत बाद की देखभाल के बारे में कुछ सामान्य प्रश्न पूछेंगे। हम आपसे सुविधा पर और घर पर एएनसी देखभाल के बारे में आपके ज्ञान, जागरूकता और अनुभव के बारे में भी कुछ प्रश्न पूछेंगे, और यह भी कि पूछेंगे कि क्या एएनसी के दौरान आपमें तीन एचआरपी विकसित हुए हैं। स्‍टडी में आपकी भागीदारी स्‍वैच्छिक है और आप भाग ना लेना या किसी भी कारण से, जब भी आप चाहे भागीदारी को रोकना चुन सकते हैं। हम आपकी सहमति के बिना आगे नहीं बढ़ेंगे। इस सर्वे में छह सेक्‍शन हैं और हमें लगता है कि इंटरव्‍यू को पूरा करने में 50-60 मिनट का समय लगेगा, लेकिन आप जितना समय चाहें ले सकती हैं।

**RISK, DISCOMFORT, AND BENEFIT**

**जोखिम, असुविधा और लाभ**

There are no major risks associated with your participation in this study, that your participation could become known to others outside the study. If you do not want to answer/respond to any questions that make you feel uncomfortable - we can simply move onto the next question. You can refuse to answer some questions or stop participating in the interview at any point during our interview. If you decline to participate or answer some questions, this decision will not impact your future access to health care or other services for you or your family. There are no benefits from your participation in this study. However, the findings from this research may help improve ANC care services in your district/UP. If you withdraw mid-stream, only with your permission data collected up to the point will be used, otherwise entire data collected from you will be deleted.

इस स्‍टडी में आपकी भागीदारी से जुड़े कोई बड़े जोखिम नहीं हैं, कि आपकी भागीदारी के बारे में स्‍टडी के बाहर अन्य लोगों को पता चल सकता है। यदि आप किसी ऐसे प्रश्न का जवाब/उत्‍तर नहीं देना चाहती हैं जो आपको असहज महसूस कराता है - तो हम अगले प्रश्न पर जा सकती हैं। आप हमारे इंटरव्‍यू के दौरान किसी भी समय कुछ सवालों का जवाब देने से मना कर सकती हैं या इंटरव्‍यू में भागीदारी रोक सकती हैं। यदि आप भाग लेने या कुछ सवालों का जवाब देने से इनकार करती हैं, तो यह निर्णय आपके या आपके परिवार के लिए स्वास्थ्य देखभाल या अन्य सेवाओं तक भविष्य की पहुँच को प्रभावित नहीं करेगा। इस स्‍टडी में आपकी भागीदारी से कोई लाभ नहीं है। हालाँकि, इस रिसर्च के निष्कर्ष आपके जिले/यूपी में एएनसी देखभाल सेवाओं को बेहतर बनाने में मदद कर सकते हैं। यदि आप बीच में ही इस स्‍टडी से बाहर निकल जाती हैं, तो उस समय तक एकत्र किए गए डेटा का इस्‍तेमाल केवल आपकी अनुमति से ही किया जाएगा, अन्यथा आपसे लिया किया गया पूरा डेटा डिलीट कर दिया जाएगा।

**CONFIDENTIALITY**

**गोपनीयता**

Your answers will not be shared with anyone outside this project. The information you provide will be kept strictly confidential; your and your baby’s names, and any personal and professional details will be removed from the interview at the time of analysis. During the discussion, I will be noting your response on a tablet. However, the noted information will be securely stored and will not be shared with anyone outside the research team. We will destroy personal information once we complete the study.

आपके जवाबो को इस प्रोजेक्‍ट के बाहर किसी से साथ साझा नहीं किया जाएगा। आपके द्वारा दी गई जानकारी को पूरी तरह से गोपनीय रखा जाएगा, आपके और आपके बच्‍चे के नाम, और किसी भी व्‍यक्तिगत या प्रोफेशनल जानकारी को विश्‍लेषण के समय इंटरव्‍यू से हटा दिया जाएगा। इस चर्चा के दौरान, मैं आपके जवाबों को टैबलेट पर नोट करूंगा। हांलाकि, हालांकि नोट की गई जानकारी को सुरक्षित तरीके से स्‍टोर किया जाएगा और रिसर्च टीम के बाहर किसी के साथ साझा नहीं किया जाएगा। हम स्‍टडी को पूरा करने के बाद आपकी व्‍यक्तिगत जानकारी को नष्‍ट कर देंगे।

**Compensation:**

क्षतिपूति**:**

We will not pay you or your family for taking part in the study.

हम स्‍टडी में भाग लेने के लिए आपको या आपके परिवार को कोई भुगतान नहीं करेंगे।

**Voluntary Participation:**

स्‍वैच्छिक भागीदारी:

Taking part in this study is voluntary, meaning you decide if you want to take part in the study. There will be no consequence to you or to your family if you choose not to take part or if you want to stop taking part at any time. If you decide not to take part or stop at any time, we will respect it and not ask you why you do not want to take part.

इस स्‍टडी में भाग लेना स्वैच्छिक है, यानि कि आप तय करती हैं कि क्‍या आप स्‍टडी में भाग लेना चाहती हैं या नहीं। आप या आपके परिवार पर इसका कोई असर नहीं होगा, और यदि आप भाग नहीं लेना चुनती हैं या किसी भी समय भागीदारी को समाप्‍त करना चाहती हैं तो इसके लिए कोई पैनल्‍टी नहीं है। यदि आप किसी भी समय भाग नहीं लेने या भागीदारी समाप्‍त करने का निर्णय लेती हैं, तो हम इसका सम्मान करेंगे और आपसे यह नहीं पूछेंगे कि आप भाग क्यों नहीं लेना चाहती हैं।

**Contact information:** This study is being conducted by ARMMAN in partnership with Government of UP, and the study protocol has been reviewed by Sigma ethics committee, that works to protect your rights and welfare. If you have additional questions or concerns or in the case of an emergency, please contact:

**संपर्क की जानकारी:** यह स्‍टडी को अरमान द्वारा उत्तर प्रदेश सरकार के साथ साझेदारी में किया जा रहा है, और स्‍टडी प्रोटोकॉल की समीक्षा सिग्मा इथिकल कमेटी द्वारा की गई है, जो आपके अधिकारों और कल्याण की रक्षा के लिए काम करती है। यदि आपके पास कोई और प्रश्न या चिंताएँ हैं या किसी आपात स्थिति में, कृपया संपर्क करें:

Name: Dr Hanimi Reddy (Principal investigator)

Mobile: 9911822445

Email: [hanimi@armman.org](mailto:hanimi@armman.org)

नाम: डॉ. हनीमी रेड्डी (प्रमुख इन्‍वेटीगेटर)

मोबाइल: 9911822445

ईमेल: hanimi@armman.org

If you have any concerns regarding your rights as a participant, please contact Sigma Research and Consulting (IRB) at [irb.sigma@sigma-india.in](mailto:irb.sigma@sigma-india.in), Phone: 011- 41063450

**CONSENT**

**सहमति**

If you are not able to understand any question/topic during the interview, please feel free to ask me to repeat. The information we are collecting is important for the improvement of health programs for mothers and new-borns in India, so we ask that you answer the questions to the best of your knowledge and ability. We have already obtained consent for your interview from your parents/in-laws to talk to you regarding this study. We will provide you with all the information related to the study; so that you can make the decision and give your voluntary assent to participate in the study.

यदि आप इंटरव्‍यू के दौरान कोई प्रश्‍न/विषय को नहीं समझ पा रही हैं, तो कृपया मुझसे बेझिझक पूछें। हम जो जानकारी एकत्र कर रहे हैं वह भारत में माताओं और नवजात शिशुओं के लिए हेल्‍थ प्रोग्राम में सुधार करने के लिए महत्वपूर्ण है, इसलिए हम आपसे अनुरोध करते हैं कि आप अपने सर्वोत्तम ज्ञान और क्षमता के अनुसार सवालों के जवाब दें।

Now, can you tell me if you **agree** to participate in this survey? अब, क्या आप मुझे बता सकती हैं कि क्या आप इस सर्वे में भाग लेने के लिए सहमत हैं? यदि आप हाँ कहती हैं, तो इसका मतलब है कि आप स्‍टडी का हिस्सा बनने के लिए सहमत हैं।

**Participant’s signature_______________________________________ Date: ___________**

**प्रतिभागी के हस्‍ताक्षर तारीख**

***If respondent cannot provide written consent:***

***यदि रिस्‍पोंडेंट लिखित सहमति नहीं प्रदान कर सकता है:***

Do you have any questions? YES NO

क्‍या आपके पास कोई प्रश्‍न हैं हां नहीं

Do you consent to participate in this study? YES NO

क्‍या आप इस स्‍टडी में भाग लेने के लिए सहमति देती हैं? हां नहीं

Please let me know if you would like to keep a copy of this form so that you can review the information at a later date, contact someone about the study, or keep it for your records.

कृपया मुझे बताएं कि क्या आप इस फॉर्म की एक कॉपी रखना चाहेंगी ताकि आप बाद में इसकी जानकारी को देख सकें, स्‍टडी के बारे में किसी से संपर्क कर सकें, या इसे अपने रिकॉर्ड के लिए रख सकें।

May we begin the interview?: Yes🡪 Start interview No🡪 End interview

क्‍या हम इंटरव्‍यू शुरू कर सकते हैं?: हां🡪 इंटरव्‍यू शुरू करें नहीं🡪 इंटरव्‍यू समाप्‍त करें
